# Synergistic induction of apoptosis by combination of BTK and dual mTORC1/2 inhibitors in diffuse large B cell lymphoma

**DOI:** 10.18632/oncotarget.2071

**Published:** 2014-06-07

**Authors:** Scott A. Ezell, Michele Mayo, Teeru Bihani, Suprawee Tepsuporn, Suping Wang, Melissa Passino, Shaun E. Grosskurth, Mike Collins, Julie Parmentier, Corinne Reimer, Kate F. Byth

**Affiliations:** ^1^ AstraZeneca R&D Boston, Waltham, Massachusetts

**Keywords:** Ibrutinib, BTK, AZD2014, mTOR, DLBCL

## Abstract

Diffuse large B cell lymphoma is generally treated by chemotherapy and there is an unmet medical need for novel targeted therapies or combination therapies. Using in vitro screening, we have identified the combination of ibrutinib, an inhibitor of the tyrosine kinase BTK, and AZD2014, an mTOR catalytic inhibitor, as being highly synergistic in killing ABC-subtype DLBCL cell lines. Simultaneous inhibition of BTK and mTOR causes apoptosis both in vitro and in vivo and results in tumor regression in a xenograft model. We identify two parallel mechanisms that underlie apoptosis in this setting: cooperative inhibition of cap-dependent translation, and the inhibition of an NF-κB/IL10/STAT3 autocrine loop. Combined disruption of these pathways is required for apoptosis. These data represent a rational basis for the dual inhibition of BTK and mTOR as a potential treatment for ABC-subtype DLBCL.

## INTRODUCTION

DLBCL is an aggressive hematological malignancy and one of the most common types of non-Hodgkin lymphoma. DLBCL is often classified as one of two subtypes: Germinal Center B cell-like (GCB) and Activated B Cell-like (ABC)[1]. Of these, the ABC subtype is more aggressive and is characterized by activation of the B cell receptor (BCR) pathway and high NF-κB activity[2], frequently through mutations in regulators of these pathways[3]. Inhibitors of BCR and NF-κB signaling are selectively toxic to ABC-type tumors[4-5], in accordance with data demonstrating that GCB-type tumors do not show activation of the BCR pathway. The current standard of care for DLBCL is R-CHOP, the combination of a monoclonal antibody, rituximab, with a cocktail of chemotherapy agents, but while this drug combination is effective in the majority of cases, many tumors are insensitive or relapse after treatment [6-7]. Overall, approximately 40% of patients do not respond to current therapies, underscoring the need for novel treatment strategies[8].

Bruton's tyrosine kinase (BTK) is a critical mediator of BCR signaling which regulates the activation of diverse pathways including NF-κB, MAPK, PKC, and AKT. BTK is essential for activation of NF-κB by the BCR through degradation of IκBα[9-10]. The discovery of an irreversible inhibitor of BTK, known as ibrutinib, represents a major advance in the therapeutic inhibition of BCR signaling. Ibrutinib has shown promise in the treatment of several hematological malignancies, including CLL[11], AML[12], and mantle cell lymphoma[13]. Initial studies have found that ibrutinib shows therapeutic activity in some ABC-type DLBCL, but not GCB-type[14]. Other specific inhibitors have shown promise as targeted therapies in DLBCL, including those for BCL2[15] and mTOR[16]. mTOR is a clinically validated target on the PI3K-Akt signalling pathway, which is the most frequently deregulated pathway in human cancers. Rapamycin and its analogues, everolimus and temsirolimus, are allosteric inhibitors of the mTOR complex mTORC1 that have been evaluated for the treatment of haematological malignancies and temsirolimus is approved for the treatment of MCL. Everolimus, an mTORC1-specific inhibitor, is cytostatic in DLBCL but does not induce apoptosis as a single agent[16]. Everolimus demonstrated proof of principle for mTORC1 inhibitors in DLBCL patients but activity was modest and resistance was frequent, possibly through an escape mechanism that involved activation of AKT which in part is regulated through mTORC2[17]. Given that single agents have achieved only moderate efficacy, the use of combination therapies is a potentially valuable approach[18].

AZD2014 is an ATP-competitive mTOR inhibitor which can block the activity of both the mTORC1 (rapamycin-sensitive) and mTORC2 (rapamycin-insensitive) complexes and is highly selective against PI3K superfamily kinases[19]. Dual mTORC1/2 inhibition is anticipated to achieve greater clinical benefit than rapalogues by abrogating a resistance feedback mechanism activated when mTORC1 is inhibited. AZD2014 has been reported to have greater potency than rapamycin against mTORC1 and a superior pharmacokinetic profile compared to some other second generation mTOR inhibitors [20]. It has been shown to have antitumor activity in vivo, in models of ER+ breast cancer and glioblastoma, and has reached Phase II clinical trials in renal cancer and ER+ breast cancer[20-21].

Here we demonstrate that the combination of ibrutinib and AZD2014 can strongly induce apoptosis in ABC-type DLBCL. The mechanisms of action of these compounds converge on the regulation of 4EBP1 and cap-dependent translation (CDT) as well as JAK/STAT3 signaling and we reveal cross-talk between the NF-κB, STAT3, and mTOR pathways. We show that inhibition of both pathways is essential for efficient apoptosis. The combination of the dual mTORC1/2 inhibitor AZD2014 with a BTK inhibitor is therefore an attractive therapeutic approach for patients with ABC-type DLBCL.

## RESULTS

### Screening identifies combination of AZD2014 and ibrutinib

**Figure 1:**
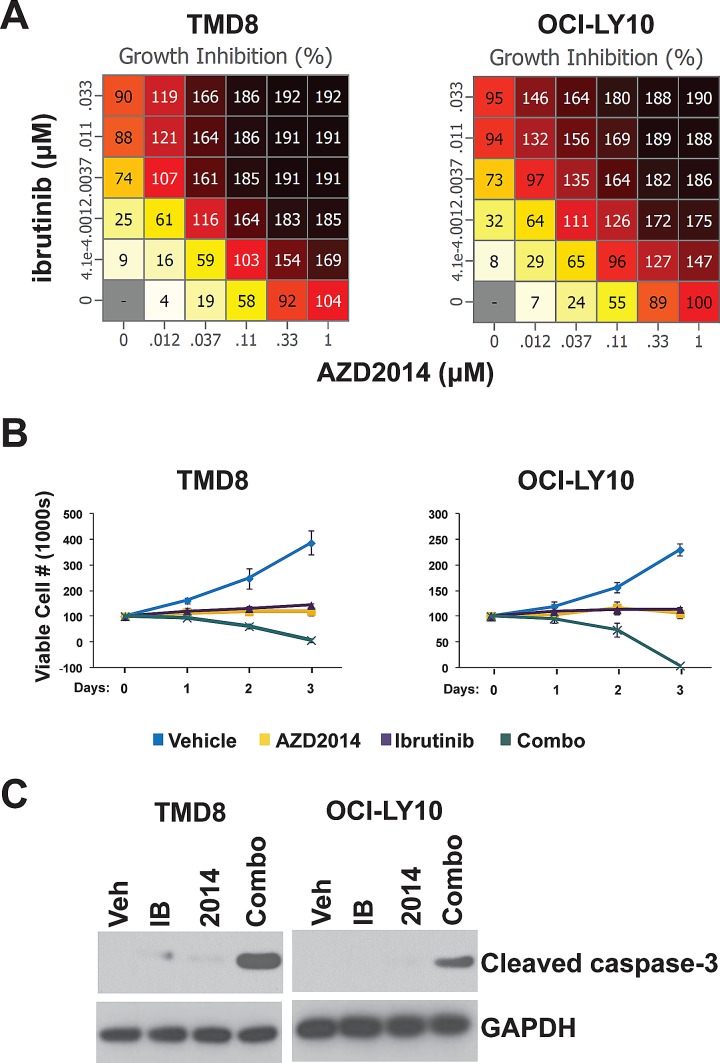
Ibrutinib and AZD2014 synergistically induce apoptosis (A) Cells were cultured with the indicated concentrations of compounds for 72 hours. The number of viable cells was measured by Alamar Blue. Numbers represent percent growth inhibition. 100 = no growth; 200 = total cell death. (B) and (C) Cells were treated with Ibrutinib (10nM) and/or AZD2014 (200nM). (B) Cells were stained with trypan blue and counted at the indicated time points. Error bars indicate standard deviation (n=3). (C) Cells were collected for Western blot analysis at 24 hours.

To identify synergistic drug combinations for the treatment of ABC-type DLBCL, we designed a screen using the ABC cell lines TMD8 and OCI-LY10. These cell lines were selected due to the presence of signature features of ABC-type DLBCL, including activating mutations in MyD88 and CD79B and high NF-κB activity[22-24]. We assembled a list of compounds targeting pathways with a known oncogenic function in lymphoma that possess single agent activity. These compounds were analyzed pairwise at a range of concentrations and cell viability was measured by Alamar Blue assay. The highest scoring combination was that of ibrutinib, an irreversible inhibitor of the tyrosine kinase BTK, and the mTORC1/2 inhibitor AZD2014 (Supplemental Table 1). As single agents, these compounds were effective in blocking proliferation but did not induce significant cell death even at high concentrations. However, in combination, AZD2014 and ibrutinib were able to cause almost complete cell death by 72 hours (Fig 1A and, and S1A). Western blotting for cleaved caspase-3 (CC3) showed that AZD2014 and ibrutinib synergistically induce apoptosis in both OCI-LY10 and TMD8 cells (Fig 1C). We also tested this combination in three GCB lines (HT, Karpas-422, and OCI-LY19) in a comparison with TMD8 to determine whether synergy is specific to the ABC subtype. None of the GCB lines showed significant synergy compared to TMD8 (Fig S1B). While AZD2014 was just as effective in blocking proliferation in GCB as in ABC lines, ibrutinib had little or no effect in GCB and the combination did not induce apoptosis in these lines. This is consistent with GCB lines being independent of BCR/NF-κB signaling.

### Gene expression signatures

**Figure 2:**
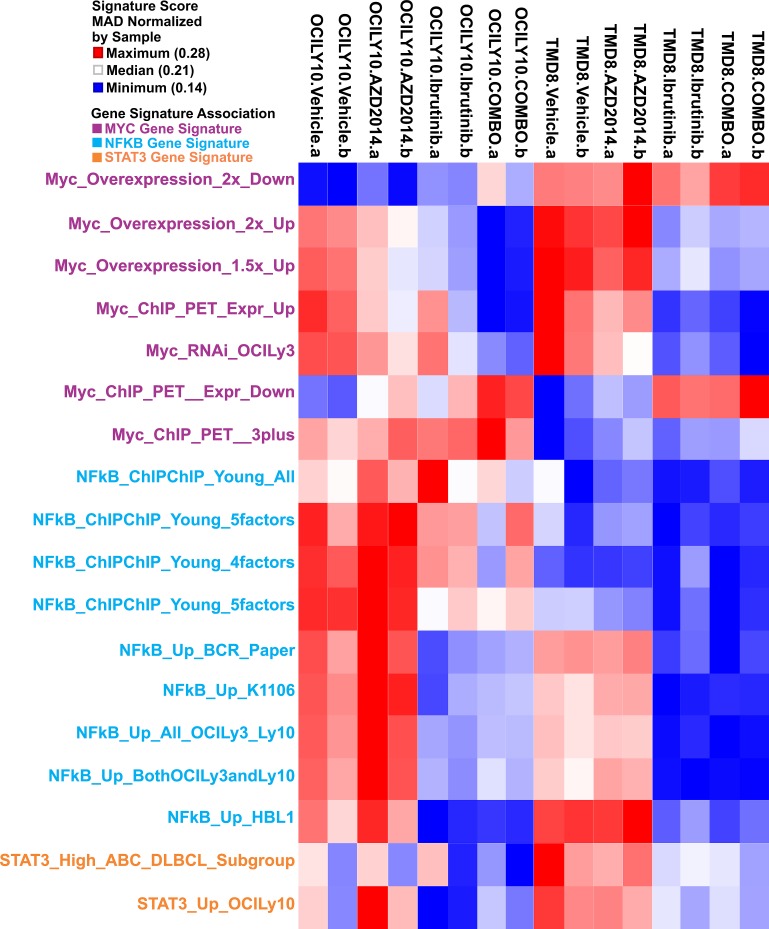
Gene expression analysis of BTK and mTOR inhibition TMD8 and OCI-LY10 cells were treated as in Figure 1 for 24 hours and RNA was collected for sequencing. Heat map represents gene signatures enriched in each condition.

We sought to understand the biological consequences of BTK and mTOR inhibition, singly and in combination, in DLBCL. Using RNA sequencing we profiled gene expression in TMD8 and LY10 at 2h, 6h, and 24h post-treatment. The 24h timepoint showed the most significant changes in gene expression and was selected for further analysis. The data was compared, using GSEA, to gene sets specifically associated with hematopoietic cells (Lymphochip) (Fig 2). Notably, both single agents showed significant downregulation of signatures relating to proliferation and the cell cycle, in accordance with our data demonstrating growth arrest (Supplemental Table 2). Signatures correlating with metabolic pathway activation were also downregulated, as expected. We also looked for gene signatures relating to known oncogenic pathways in DLBCL. We found that ibrutinib resulted in downregulation of NF-κB target genes, as expected. While AZD2014 had only modest effects on NF-κB signatures, notably among the genes most strongly repressed by AZD2014 were several known NF-κB target genes including CCL3 and CCL4 (Supplemental Table 3). Signatures associated with the proto-oncogene c-MYC were strikingly downregulated by ibrutinib and to a lesser extent by AZD2014, with the combination having an even more profound effect than ibrutinib alone. Finally, STAT3 target genes were dowregulated by treatment with Ibrutinib; AZD2014 had a lesser effect. High STAT3 activity has been shown to predict poor prognosis in DLBCL[25]. These gene signatures indicate that Ibrutinib and AZD2014 can modulate signaling pathways with known oncogenic function in DLBCL.

### Inhibition of STAT3 through an autocrine loop

**Figure 3:**
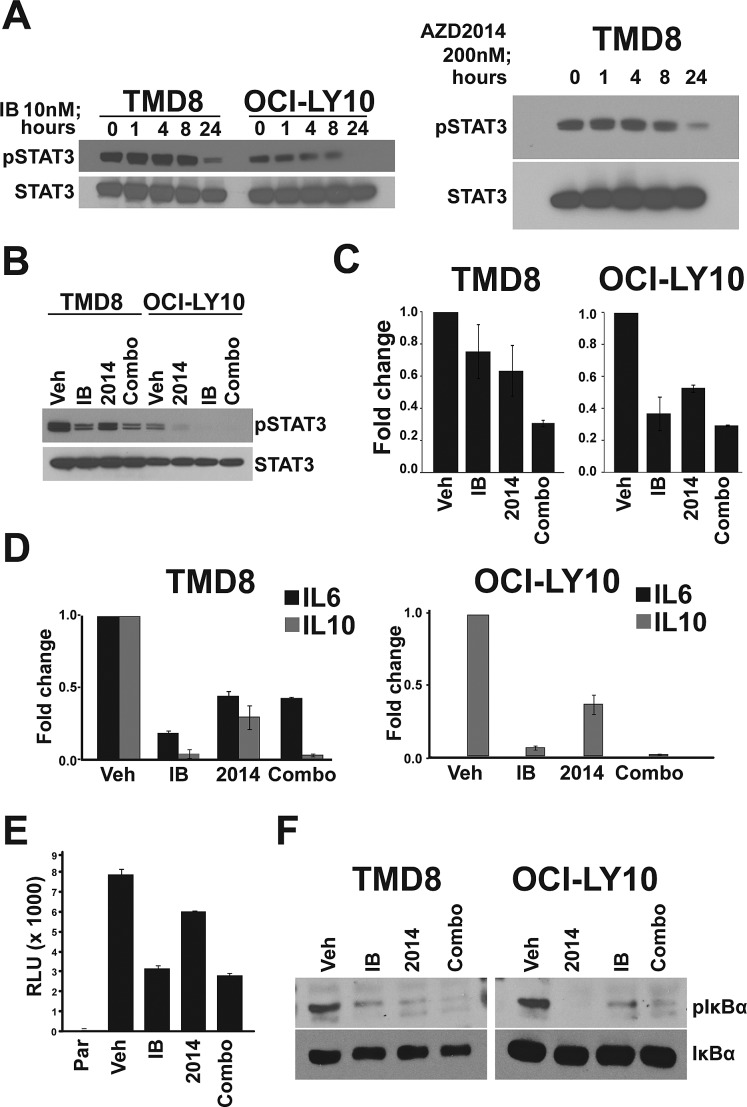
An autocrine loop controls STAT3 activation (A) and (B) Cells were treated for indicated times and harvested for Western blot. (C) Cells were treated for 24 hours and supernatant was collected for IL-10 ELISA assay. Error bars indicate standard deviation (n=3). (D) Cells were treated for 24 hours and RNA was collected for real-time PCR. Error bars indicate standard deviation (n=3). (E) TMD8 cells stably expressing an NF-kB luciferase reporter were treated for 18 hours before measurement. Error bars indicate standard deviation (n=3). (F) Cells were treated for 24 hours and harvested for Western blot.

Having identified the NF-κB and STAT3 pathways as being modulated by ibrutinib and perhaps also AZD2014, we assessed the kinetics of pathway inhibition by Western blot. We found that phosphorylation of STAT3 at Y705, which is mediated by JAK kinases and is critical for its activation, is inhibited at 24h by ibrutinib and AZD2014, but not at earlier timepoints (Fig 3A). NF-κB pathway activation, as measured by phosphorylation of IκBα, was reduced by 8h, preceding STAT3 inhibition (Fig S2). We selected the 24 hour timepoint for further analysis since it showed the greatest inhibition. We found that ibrutinib strongly reduces pSTAT3 and that AZD2014 does so to a lesser degree (Fig 3B). The combination of AZD2014 with ibrutinib did not inhibit pSTAT3 to a greater extent than ibrutinib alone. The kinetics of STAT3 inhibition suggest that it is not a direct target of either BTK or mTOR but might be regulated indirectly. JAK/STAT3 is regulated by a number of cytokines, including IL6 and IL10[26]. Cytokine profiling demonstrated that both TMD8 and LY10 cells strongly express IL10 but IL6 is only produced at low levels in TMD8 and is nearly undetectable in OCI-LY10 (Fig S3). The receptors for both IL6 and IL10 were expressed on the cell surface of both TMD8 and LY10, suggesting that an autocrine loop may be in effect in these cells (Fig S3). Measurement of IL10 in conditioned medium by ELISA demonstrated that treatment with either ibrutinib or AZD2014 reduced secreted IL10 (Fig 3C). We measured transcription of IL10 as well as IL6 by quantative PCR. Both ibrutinib and AZD2014 reduced mRNA expression of both genes (Fig 3D). IL6 and IL10 are known targets of NF-κB[27-28]. To directly measure the inhibition of NF-κB signaling, we created a stable NF-κB reporter line from TMD8 cells. Treatment of these cells with either ibrutinib or AZD2014 inhibited luciferase activity, confirming that both BTK and mTOR are upstream regulators of this pathway in DLBCL (Fig 3E). The same result was obtained in 293T cells transfected with a luciferase reporter construct (Fig S4). Western blot analysis showed that both ibrutinib and AZD2014 treatment reduce phosphorylation of IκBα at 24h and levels of pIκBα correlate with pSTAT3 (Fig 3F). Collectively, these data suggest that a reduction in secreted cytokines as a result of NF-κB inhibition leads to reduced STAT3 activation.

### Synergistic inhibition of cap-dependent translation

**Figure 4:**
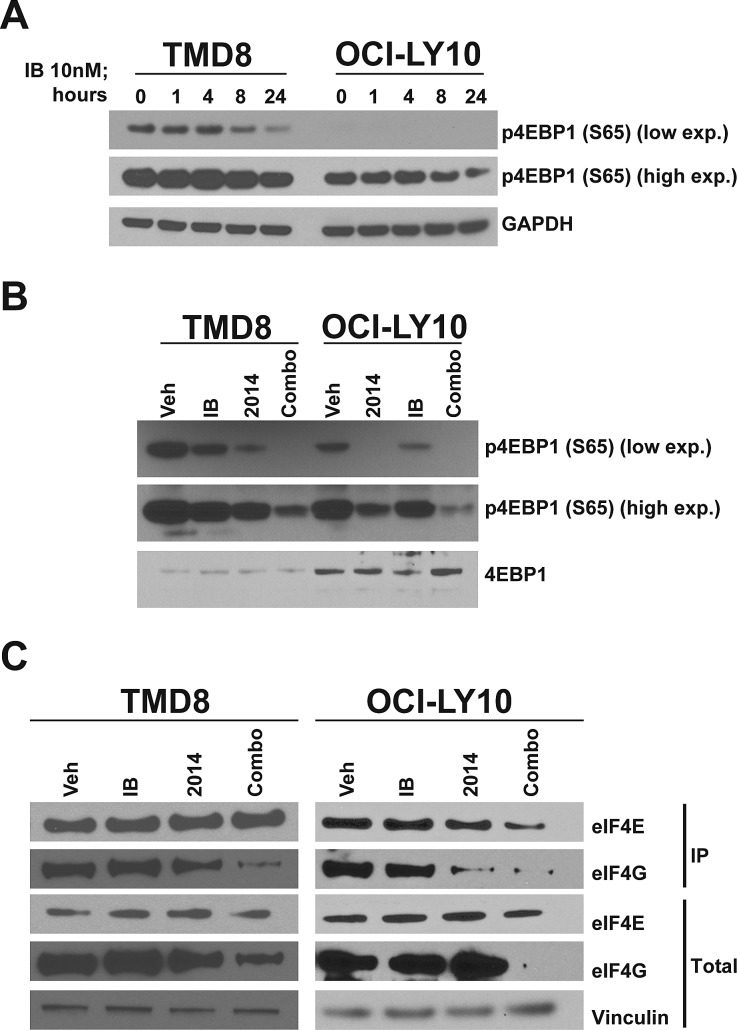
Synergistic inhibition of cap-dependent translation (A) Cells were treated for the indicated times and harvested for Western blot. (B) As in (A) but treated for 24 hours. (C) Cells were treated for 24 hours and lysates were collected for 7-methyl-GTP cap-binding assay.

The mTOR pathway is an extremely important regulator of tumorigenesis in a variety of models and controls both proliferation and apoptosis. As expected, treatment with AZD2014 rapidly reduced phosphorylation of the mTOR substrate 4EBP1 as well as PRAS40, an indirect target of mTORC2 (Fig S2A). Inhibition was sustained for at least 24 hours post-treatment. However, even treatment with a high dose did not fully inhibit 4EBP1 phosphorylation at S65 in these cell lines. We decided to determine whether ibrutinib could also affect the mTOR pathway. While ibrutinib did not inhibit 4EBP1 phosphorylation at early time points, by 24 hours it markedly reduced phosphorylation (Fig 4A). Surprisingly, in combination with AZD2014, ibrutinib was able to reduce 4EBP1 phosphorylation significantly compared to AZD2014 alone (Fig 4B). While ibrutinib treatment alone was able to inhibit phosphorylation of S6, another mTOR target, at 24 hours, combination treatment did not further reduce phosphorylation (Fig S5A). These data suggest that while ibrutinib can reduce total mTOR activity it may also regulate mTOR-independent mechanisms that control 4EBP1 phosphorylation.

4EBP1 is a regulator of cap-dependent translation through its interaction with eIF4E. Phosphorylation of 4EBP1 at S65 inhibits its interaction with eIF4E and permits the association of eIF4E with eIF4G and thus translational initiation[29-30]. Since AZD2014 and ibrutinib in combination synergistically inhibit S65 phosphorylation, we used 7-methyl-GTP beads to measure assembly of the initiation complex required for CDT. In agreement with our data on 4EBP1 S65 phosphorylation, we found that combination treatment inhibited binding of eIF4G to the cap to a much greater extent that treatment with either single agent (Fig 4C). The proto-oncogene c-MYC, a target of eIF4E, is translated through a cap-dependent mechanism[31]. Inhibition of CDT in DLBCL results in downregulation of c-MYC mRNA expression[32]. As discussed earlier, GSEA identified MYC as being strongly downregulated with ibrutinib/AZD2014 treatment. We measured c-MYC protein levels by Western blot and found that indeed c-MYC is synergistically downregulated by ibrutinib and AZD2014, in accordance with our hypothesis (Fig S5B). c-MYC is an important driver of ABC-type DLBCL[33]; therefore downregulation of c-MYC expression through activation of 4EBP1 may contribute to the effect of ibrutinib/AZD2014. In order to determine whether apoptosis was the result of c-MYC downregulation, we made use of the BRD4 inhibitor JQ1, which is known to downregulate c-MYC transcription[34]. Treatment of TMD8 with JQ1 resulted in very strong downregulation of c-MYC (Fig S5C) but little or no reduction in cell viability relative to the ibrutinib/AZD2014 combination (Fig S5D). This demonstrates that c-MYC downregulation is not sufficient to explain apoptosis in this context.

### Rescue of translation and STAT3 signaling prevents apoptosis

**Figure 5:**
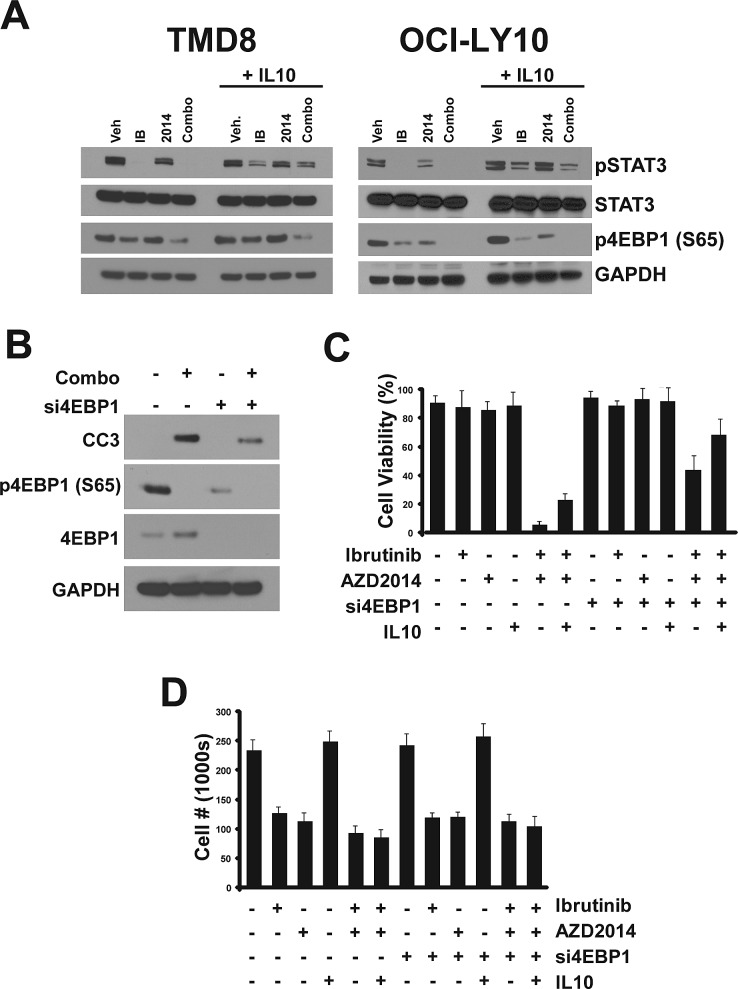
Determinants of apoptosis (A) Cells were treated with compounds for 24 hours. 90 minutes before collection for Western blot, cells were treated with recombinant human IL10 (50ng/ml). (B) Cells were transfected with 1uM siRNA pool targeting 4EBP1. 96 hours after transfection, cells were treated with compounds and collected 24 hours later for Western blot. (C) Cells were transfected and treated as in (B) except for 72 hours. IL10 treatment (50ng/ml) was performed twice, at 24 hours and 48 hours before trypan blue staining and counting. Error bars indicate standard deviation (n=3).

Having identified pathways perturbed by ibrutinib and AZD2014 treatment, we sought to understand how these compounds induce apoptosis. We tested the importance of IL10/JAK/STAT3 signaling in apoptosis by stimulating drug treated cells with exogenous IL10. IL10 stimulation partially restored pSTAT3 levels without affecting 4EBP1 S65 phosphorylation in cells treated with the inhibitors (Fig 5A). This suggests that inhibited STAT3 signaling is a consequence of reduced cytokine secretion downstream of NF-κB. However, IL10 alone did not have a major effect on cleaved caspase-3 (data not shown).

Given that inhibition of 4EBP1 phosphorylation and cap-dependent translation by the combination correlates with cleavage of caspase-3 and cell death, we conjectured that apoptosis induction by this combination occurs as a result of reduced translation. In order to test this hypothesis we used siRNAs targeting 4EBP1 which, by liberating eIF4E from the inhibitory interaction with 4EBP1, should result in constitutively high cap-dependent translation. We found that pooled siRNAs could almost completely knockdown 4EBP1 protein expression in OCI-LY10 cells. Indeed, when control and si4EBP1-treated cells were treated with the combination, we observed a significant reduction in cleaved caspase-3 with the knockdown (Fig 5B). This suggests that reducing cap-dependent translation, which requires both inhibitors, is critical for the activation of the apoptotic response.

These data led us to explore whether inhibition of translation and JAK/STAT3 act together to cause apoptosis. We tested combinations of treatments for their effect on cell viability. We confirmed that treatment with ibrutinib or AZD2014 alone did not result in reduced viability but effectively blocked proliferation (Fig 5C and). The combination, as expected, caused almost complete cell death within 72 hours. Addition of exogenous IL10 alone had only a modest protective effect on cells. Knockdown of 4EBP1 alone had a greater protective effect, although the majority of cells still underwent apoptosis. However, when si4EBP1 and IL10 were both administered, apoptosis as a result of ibrutinib/AZD2014 treatment was largely blocked. Interestingly, while these treatments prevented apoptosis, they did not restore proliferation, suggesting that the mechanisms through which ibrutinib and AZD2014 arrest the cell cycle are distinct from those that govern cell death.

### Combined inhibition of mTOR and BTK is effective *in vivo*

**Figure 6:**
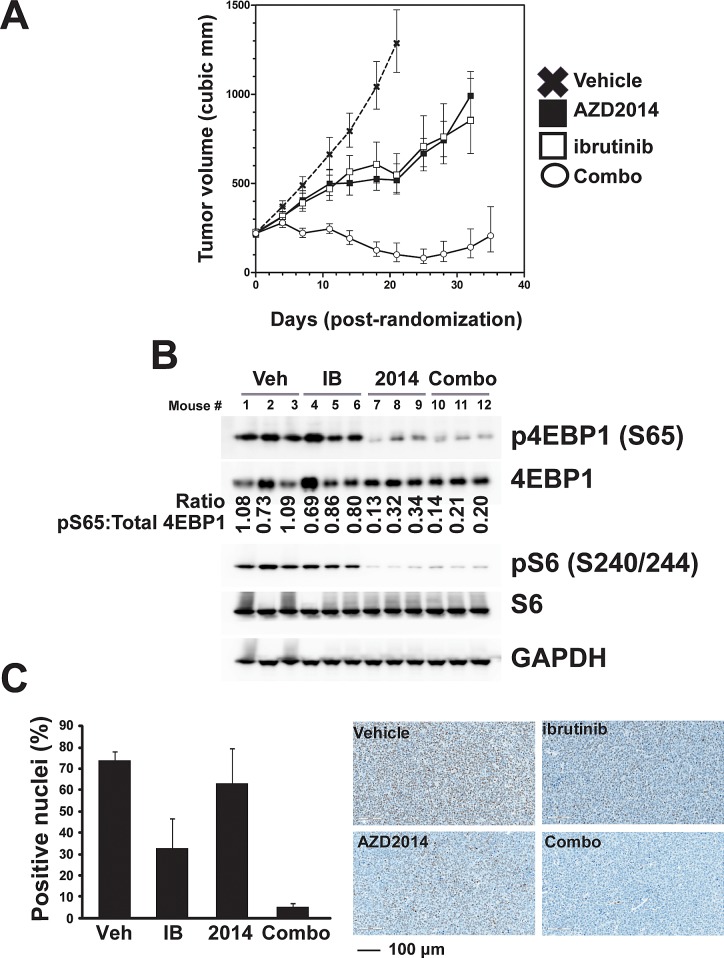
Combined inhibition of mTOR and BTK is effective *in vivo* (A) OCI-LY10 xenografts were established, randomized, and then dosed daily for 25 days (ibrutinib at 12 mg/kg and AZD2014 at 15 mg/kg). Error bars indicate SEM. (B) Western blot analysis of tumor lysates collected after 4 hour acute dosing. (C) pSTAT3 Y705 imunohistochemistry was performed on fixed tumor sections after 4 hour acute dosing.

The antitumor activity of AZD2014, Ibrutinib, and combination of AZD2014 and Ibrutinib were evaluated in female SCID mice bearing OCI-LY10 tumors. Twenty four days after tumor cell inoculation mice were randomized into treatment groups, vehicle control, AZD2014 (15 mg/kg), ibrutinib (12 mg/kg), and combination of AZD2014 (15 mg/kg) and ibrutinib (12 mg/kg). Measurements of tumor growth inhibition (TGI), and partial and complete regression are shown in Figure S6A. Daily oral treatments with AZD2014 and ibrutinib alone resulted in significant (p-value, <0.0001) tumor growth delay (72% and 79%, TGI, respectively) in OCI-LY10 xenografts. However, neither treatment caused any partial or complete regressions. Treatment with the combination of AZD2014 and ibrutinib demonstrated synergistic anti-tumor activity with >100% TGI and five of the nine animals displaying partial regressions and one animal resulting in complete regression (Fig 6A and Fig S6). All treatments were well tolerated with no body weight loss as with vehicle control animals, (data not shown). To confirm our in vitro mechanistic studies, we performed Western blot analysis of lysates collected from LY10 xenografts after treatment. These tumors showed synergistic inhibition of 4EBP1 phosphorylation as in vitro, while S6 phosphorylation showed no such effect (Fig 6B). We confirmed inhibition of pSTAT3 by immunohistochemical analysis of tumor sections, in order to exclude the possibility of stromal contamination. Combined treatment strongly reduced the percentage of tumor cells positive for pSTAT3 in the nucleus (Fig 6C). The striking synergistic inhibition of pSTAT3 found in vivo did not match our finding in vitro that combination treatment was not more effective than ibrutinib alone. We hypothesized that this may be the result of lower effective concentration of the inhibitors in vivo compared to in vitro. To test this, we performed a dose response in vitro. We saw that at low concentrations of Ibrutinib, AZD2014 treatment was able to further inhibit pSTAT3 (Fig S6B). These data demonstrate that the combination of ibrutinib with AZD2014 is highly effective in ABC-type DLBCL and acts through concerted inhibition of multiple pathways to induce apoptosis.

## DISCUSSION

Current targeted therapies have achieved only partial success in targeting ABC-type DLBCL. We have used in vitro screening technology to identify the novel drug combination, ibrutinib and AZD2014, as being highly and specifically cytotoxic to ABC DLBCL. While ibrutinib or AZD2014 alone are able to efficiently prevent cell proliferation, neither induces significant levels of apoptosis, as measured by two cell viability assays and caspase-3 cleavage. The combination of these two agents, however, induces nearly universal apoptosis in treated cells by 72 hours. We extended this finding in vivo by showing that in OCI-LY10 xenografts ibrutinib and AZD2014 alone are able to delay tumor growth, while the combination results in significant tumor regression. This synergistic combination effect appears to be specific to ABC-type DLBCL, as several GCG cell lines failed to respond similarly.

We have also investigated the mechanism through which this drug combination is able to induce apoptosis. By gene expression analysis and profiling of relevant pathways, we found that STAT3 phosphorylation is inhibited by both ibrutinib and AZD2014. Inhibition only takes place at later timepoints, suggesting an indirect mechanism. This could be explained by a reduction in the transcription of cytokines which may act in an autocrine loop to stimulate STAT3 phosphorylation through JAK. Downregulation of cytokine expression appears to be the result of NF-κB inhibition by these compounds, although we cannot rule out an additional effect on translation. While BTK is known to be an important regulator of NF-κB in DLBCL, little information exists linking mTOR to NF-κB regulation. While mTOR has been reported to associate with the IKK complex to stimulate NF-κB in prostate cancer cells[35], to our knowledge mTOR has not been demonstrated to be a driver of NF-κB activity in DLBCL.

We have also found that concomitant treatment of cells with ibrutinib and AZD2014 reduces phosphorylation of 4EBP1, an established mTOR target, much more efficiently than AZD2014 alone. This raises the question of how ibrutinib is inhibiting phosphorylation of this target. Combination treatment did not inhibit phosphorylation of S6, another downstream target of mTOR, to a greater extent than AZD2014 alone. This suggests that BTK regulates 4EBP1 phosphorylation through a pathway distinct from mTOR. Several additional kinases have been reported to regulate 4EBP1 including PIM1[36], p38α[37], and LRRK2[38]. It is possible that BTK regulates translation through the regulation of another kinase, making cap-dependent translation partially independent of mTOR inhibition. Our data support the intriguing possibility that mTOR inhibition is not sufficient in DLBCL to significantly reduce cap-dependent translation. Furthermore, we found that loss of 4EBP1 and restoration of autocrine IL10 signaling can mostly block the activation of apoptosis in cells treated with ibrutinib and AZD2014.

Collectively, our data support a rational basis for the co-inhibition of the kinases BTK and mTOR in ABC-type DLBCL. The efficacy of combined treatment with ibrutinib and PI3K/mTOR inhibitors in DLBCL has been demonstrated in vitro[39]. Our data extend these findings in vivo to show that AZD2014 and ibrutinib can robustly inhibit tumor growth and even cause tumor regression. Our mechanistic studies, supported by in vivo biomarkers, provide an explanation for the synergistic activation of the apoptotic response. We also identify significant crosstalk between the mTOR, NF-κB, and JAK/STAT3 pathways in DLBCL. In summary, simultaneous inhibition of BTK and mTOR may be of significant clinical benefit.

## MATERIALS AND METHODS

### Cell culture

TMD8 cells (Tokyo Medical and Dental University) were grown in MEMα supplemented with 10% FBS, penicillin/streptomycin, and glutamine. OCI-LY10 cells (Ontario Cancer Institute) were grown in IMDM supplemented with 20% FBS, penicillin/streptomycin, and glutamine. Compounds were dissolved in DMSO. Unless otherwise noted, all treatments with ibrutinib were performed at 10nM; 200nM for AZD2014. Accell siRNAs were purchased from ThermoFisher Scientific and were transfected at 1μM according to manufacturer's instructions. Recombinant IL10 was purchased from Cell Signaling Technology. Cells were counted and viability was assessed using a Cellometer Auto 2000 (Nexcelom). 0.4% Trypan blue solution was purchased from MP Biomedicals.

### Matrix-based screening

Cells were seeded onto 96-well plates (20,000 cells/well) 18 hours prior to treatment with AZD2014 and ibrutinib either singly or in combination in a matrix-based 6 dose × 6 dose design. Alamar blue (10 μL, 10% of volume per well) was added and the cells were further incubated at 37°C for 4 hours. Fluorescence with excitation wavelength at 530–560 nm and emission wavelength at 590 nm was measured. Cell growth was determined at day zero (V_0_) before compound dosing and after 72 h of treatment (T, V). Chalice Analyzer (Zalicus Inc. Cambridge, MA) was used to convert the data to 0-200% growth inhibition using the formula T<V_0_ ? 100*(1-(T-V_0_)/V_0_) : 100*(1-(T-V_0_)/(V-V_0_)). This supports interrogation of the data with cytostatic effects in the 0-100% range and cytotoxic effects in the 100-200% range. Synergy was measured by determination of combination index (CI) or synergy score by comparison to the Loewe additivity model.

### Viral Transduction

DOTAP (10 μg/ml; Roche) was added to viral supernatants and incubated at 4°C for 10 minutes. Cells were centrifuged, resuspended in viral supernatant/DOTAP mixture, and then spun at 2500 rpm for 90 minutes at 32°C. And equal volume of growth medium was added and cells were allowed to recover for 48 hours before puromycin selection (0.5 μg/ml escalating to 1.0 μg/ml after one week).

### Luciferase assays

Luciferase assays were performed using Dual-Glo (Promega). Virus containing the pGreenFire NF-κB reporter plasmid was purchased from System Biosciences and used to transduce TMD8 cells. Cells were treated for 18 hours before lysis. 293T cells were transfected with luciferase vector using Fugene 6. Twenty four hours after transfection, cells were treated with compounds for 4 hours before lysis.

### Western blotting

Cells were lysed in 1X lysis buffer (Cell Signaling) supplemented with protease/phosphatase inhibitor cocktail (Cell Signaling). Protein was quantified using the Pierce BCA assay kit. 4X Sample Buffer and 10X Reducing Agent (Life Technologies) were added and samples were boiled for 5 minutes before loading. 4-12% Bis-Tris NuPAGE gels were used with NuPAGE MES SDS Running Buffer. Protein was transferred to Novex Nitrocellulose membranes using NuPAGE Transfer Buffer. Protein was detected using SuperSignal West Dura and West Femto ECL (Pierce).

### Cap-binding Assay

Cells were lysed in IP buffer (Cell Signaling), quantified using the BCA protein assay kit (Pierce) and 200ug of total protein was incubated with 20ul of m^7^-GTP-sepharose beads (GE Healthcare) overnight at 4°C. Beads were centrifuged and washed three times in IP buffer. After the final wash, the beads were boiled in Laemmli Buffer, separated by SDS-PAGE, and transferred to nitrocellulose membranes. Western blot analyses were performed using the indicated antibodies from Cell Signaling Technologies: α-eIF4G (#2498) and α-eIF4E (#9742).

### qPCR

RNA was extracted using Qiagen RNeasy kits and cDNA was synthesized using High Capacity cDNA Reverse Transcription Kit (Applied Biosystems). qPCR analysis was performed using Fast SYBR Green Master Mix (Applied Biosystems) on a ABI Prism 7900HT. The following primers were used: IL6 (5’-ACTCACCTCTTCAGAACGAATTG-3’; 5’-CCATCTTTGGAAGGTTCAGGTTG-3’), IL10 (5’-TCAAGGCGCATGTGAACTCC-3’; 5’-GATGTCAAACTCACTCATGGCT-3’).

### ELISA and Luminex

Cell lines were plated out in 6 well dishes at 10^6^ cells/well in 5ml of culture media for 3 days. Supernatant was harvested and concentrations of IL-6 and IL-10 were determined using Luminex technology (Bio-Plex Suspension Array System, Bio-Rad), following the manufacturer's instructions. Samples were used neat and incubated for 30 minutes (room temperature, 300 rpm agitation) with capture antibody-coupled magnetic beads. Following three washes, samples were incubated for 30 minutes in the dark (room temperature, 300 rpm agitation) with biotinylated detection antibody. Each captured analyte was detected by the addition of streptavidin-phycoerythrin and quantified using a BioPlex 100 array reader. Analyte concentrations were calculated with Bio-Plex Manager software. For ELISA assays, supernatants were collected after centrifugation at indicated timepoints and then diluted 1:10 in plain medium. IL10 concentration was measured the HUMAN IL-10 ELISA Kit II from BD Biosciences.

### Flow Cytometry

Cells were washed once in PBS and resuspended in PBS + 2% human serum for 10 minutes on ice. Surface staining for IL-6R, IL-10R, and IgM (BD Pharmingen) was performed in the dark for 30 minutes at 4°C. Cells were washed twice in PBS + 2% FBS and fixed with 2% paraformaldehyde. Analysis was performed on a FACS Canto II (Becton Dickinson). FlowJo software (TreeStar, Inc.) was used to analyze flow cytometry data.

### Immunohistochemistry

OCI-LY10 tumors were harvested, trimmed and immediately fixed in 10% neutral buffered formalin for 24 hours. Fixed tumors were subjected to routine vacuum processing through graded ethanol, xylene and paraffin. Tumors were then embedded into paraffin blocks and routinely sectioned at a thickness of 3μm. IHC was run with an optimized protocol on the Ventana Discovery XT using anti-Phospho-STAT3 primary antibody (Cell Signaling Technologies CST9145), biotinylated anti-rabbit secondary antibody (Vector Labs PK-6101) and the DABMap detection kit (Ventana Medical 760-124). Once coverslipped, digital slide images were acquired with the Aperio Scanscope XT using a 20X objective. Viable tumor areas were manually selected in Aperio Imagescope (version 11), and percent positive nuclei were quantified using a modified Nuclear Algorithm (version 9).

### *In vivo* experiments

Female CB.17 SCID mice, five to six weeks old, were obtained from Charles River Laboratories (Wilmington, MA). Upon receipt, the mice were quarantined for minimum of three days prior to study initiation. The mice showed no signs of disease or illness upon arrival or prior to study initiation. The mice were maintained in accordance with the *Guide for the Care and Use of Laboratory Animals* (National Research Council) and food and water were available *ad libitum*. OCI-LY10 tumor cells (5.0 × 10^6^) in serum-free medium with matrigel (1:1 ratio) were injected subcutaneously into the area under the right flank of each mouse. Tumors were allowed to reach a volume of approximately 200 mm^3^ prior to randomization into four treatment groups (n = 9 per group) by tumor volume. Treatments were administered daily orally. A mouse was considered to have a partial regression (PR) when tumor volume was reduced by 50% or greater, complete tumor regression (CR) when no palpable tumor could be detected. AZD2014 was prepared at 3 mg/ml in 20% captisol. Ibrutinib was prepared at 2.4 mg/ml in 0.5% methyl cellulose.

## SUPPLEMENTARY METHODS, MATERIAL, FIGURES AND TABLES


