# Supplementary material for: Synergistic induction of apoptosis by combination of BTK and dual mTORC1/2 inhibitors in diffuse large B cell lymphoma
Source: Oncotarget. 2014 Jun 7;5(13):4990–5001. doi: 10.18632/oncotarget.2071 (PMC4148116; doi:10.18632/oncotarget.2071)
Supplement: Supplementary file 1 [file oncotarget-05-4990-s001.pdf]

Synergistic induction of apoptosis by combination of BTK and dual mTORC1/2 inhibitors in diffuse large B cell lymphoma

Supplementary Methods and Material

RNA sequencing

After treatment, cell pellets were sent to Expression Analysis (http://www.expressionanalysis.com) who performed RNA isolation, cDNA library generation, Illumina HiSeq RNAseq at 12 million read depths, and generation of the read FASTQ files. Bcbio-nextgen (https://github.com/chapmanb/bcbio-nextgen) was used for automated high throughput sequencing analysis which included read quality control, alignment to the human genome hg19, and quantification of the raw sequence data to Ensembl gene annotation.

For BROAD GSEA, the GUI provided at http://www.broadinstitute.org/gsea/index.jsp was downloaded and used to perform analysis. A tab delimited .gct and .cls file was generated separately for the TMM normalized log2 gene count+1 data for the 24 hour cell line (OCI-Ly10 or TMD8) treated (ibrutinib, AZD2014, or combination) versus the vehicle respectively. For each analysis, the .gct, .cls, and .gmt (c2.all.v4.0.symbols.gmt from GeneSigDB [3] at or Staudt lab lymphoid signatures [4]) files were loaded into GSEA. When running GSEA, the following parameters were used: 1000 permutations, treated versus control phenotype labels, false for collapse to gene symbol, gene_set for permutation type, 500 for max size exclusion, 15 for min size exclusion, chip platforms RefSeq_human.chip, and Diff_of_Classes when performing metric for ranking genes. Gene sets with normalized enrichment scores (NES) >1 or <-1 were considered significant. Results were transferred to Excel for parsing. For the gene signature scoring, the average log2 expression for the genes in each signature was generated for each cell. Then the signature scores were MAD normalized by sample. If the signature had a significant enrichment score and associated with MYC, NFKB, or STAT3 signaling; the MAD normalized scores were used for generating the MAD signature complete linkage correlation hierarchical clusters.

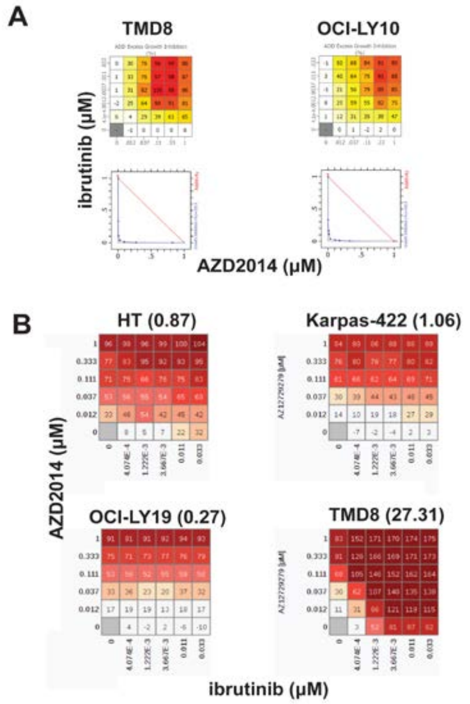

Supplemental Figure 1: Ibrutinib and AZD2014 synergistically induce apoptosis. (A) Excess growth inhibition and isobologram analysis for Figure 1A. (B) Synergy scores for the indicated cells lines. Analysis was peformed as in Fig 1A. AZ13566216 = ibrutinib; AZ12729279 = AZD2014.

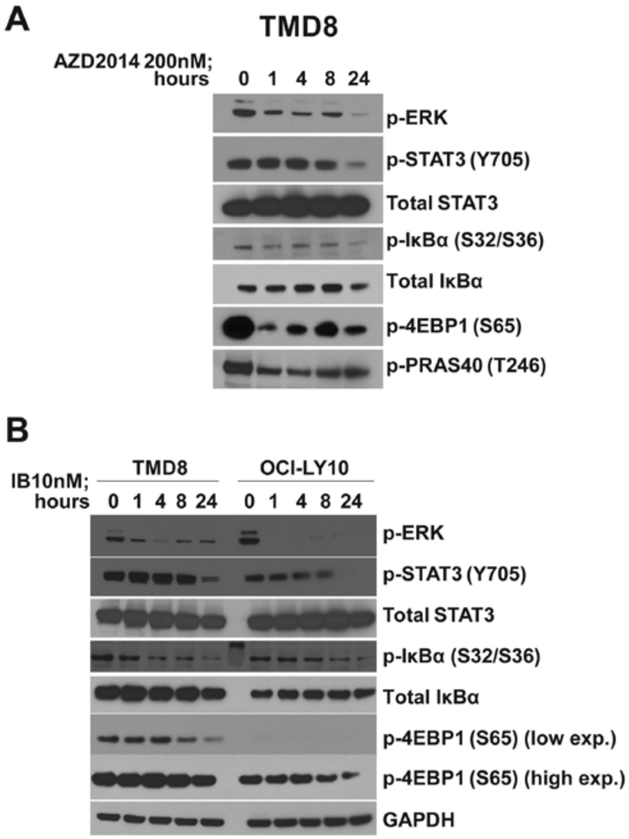

Supplemental Figure 2: Timecourse analysis. (A) and (B) Cells were treated for the indicated times and collected for Western blot analysis.

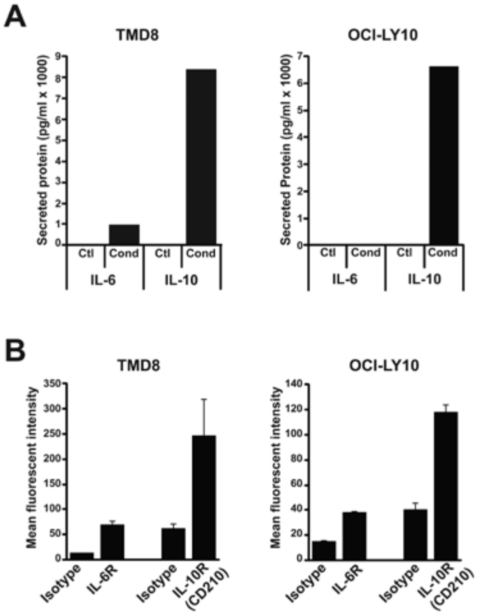

Supplemental Figure 3: Cytokine and receptor profiling. (A) Conditioned media was collected from cells after 3 days and cytokine levels were measured by Luminex. (B) Surface receptor expression was measured by flow cytometry using fixed cells.

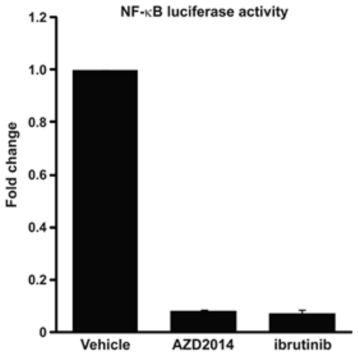

Supplemental Figure 4: Ibrutinib and AZD2014 inhibit NF-kB. 293T cells were transfected with luciferase reporter; 24 hours after transfection, cells were treated with either Ibrutinib (10nM) or AZD2014 (200nM) for 24 hours. Error bars indicate standard deviation (n=3).

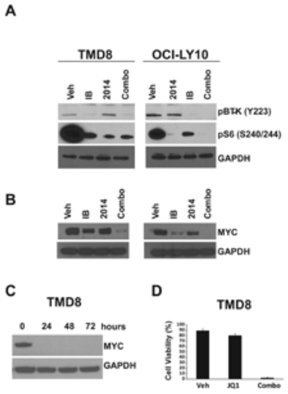

Supplemental Figure 5: Additional Western blot analysis. (A) and (B) Cells were treated for 24 hours with the indicated compounds and collected for Western blot analysis. (C) Cells were treated with 500nM JQ1 for the indicated time points and collected for Western blot analysis. (D) Cell viability was assessed by trypan blue staining 72h after treatment with 500nM JQ1 or 10nM ibrutinib/200nM AZD2014.

A

Treatment	Dose (mg/kg)	Schedule	Tumor Growth Inhibition (Day 21)	p-value (Day 21)	PR	CR	n
Vehicle	-	qd (po)	-	-	0/9	0/9	9
AZD2014	15	qd (po)	72%	<0.0001	0/9	0/9	9
Ibrutinib	12	qd (po)	70%	<0.0001	0/9	0/9	9
AZD2014 & Ibrutinib	15<br>12	qd(po)<br>qd(po)	>154%	<0.0001	5/9	1/9	9

PR, partial regression
CR, complete regression

B

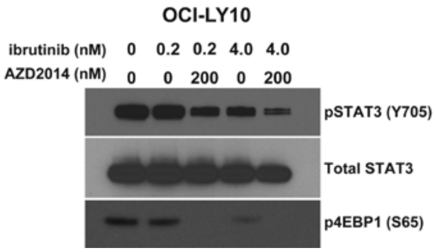

Supplemental Figure 6: Additional in vivo data. (A) Additional data collected from in vivo experiment in Figure 6. (B) Cells were treated for 24 hours with the indicated concentrations of compounds and collected for Western blot analysis.

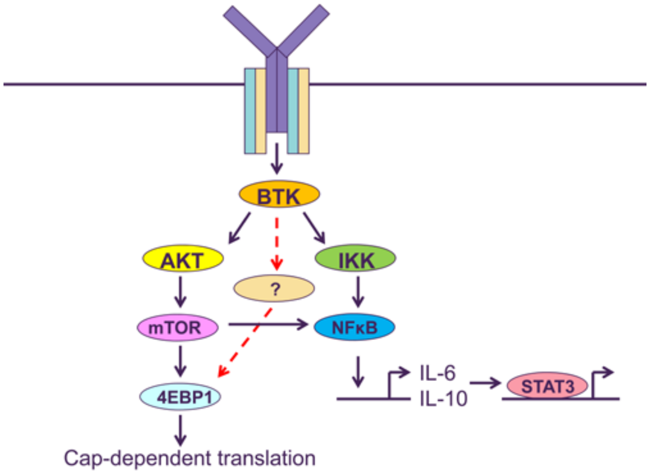

Supplemental Figure 7: Pathways regulated by ibrutinib and AZD2014. AZD2014 affect cap-dependent translation through effects on 4EBP1 phosphorylation. This may involve regulators other than mTOR. In addition, both can affect STAT3 activation through NF-kB inhibition.

Supplemental Table 1: Additional synergy scores. Synergy scores for drug combinations in TMD8 and OCI-LY10, derived as in Figure 1A.

**Combinations with Ibrutinib**	**OCI-LY10**		**TMD8**	
**Class**	**Synergy score**	**best CI**	**Synergy score**	**best CI**
mTORC1/2 inhibitor (AZD2014)	27	0.0905	35	0.063
BCL2 inhibitor	13	0.18	10	0.31
JAK2 inhibitor	13	0.215	11	0.535
PIM inhibitor	8	0.4	13	0.49
JAK1 inhibitor	8	0.125	17	0.092
JAK1/3 inhibitor	8	0.42	16	0.4
BCL2/XL inhibitor	8	0.25	10	0.4
AKT inhibitor	4	0.42	12	0.21
**Combinations with AZD2014**	**OCI-LY10**		**TMD8**	
**Class**	**Synergy score**	**best CI**	**Synergy score**	**best CI**
BTK inhibitor (Ibrutinib)	29	0.071	35	0.063
PI3kδ inhibitor	16	0.15	33	0.059
PI3kβδ inhibitor	16	0.33	22	0.21
BCL2/XL inhibitor	7.1	0.29	10	0.23
BCL2 inhibitor	6.8	0.41	8	0.32
JAK2 inhibitor	5.4	0.39	4.7	0.88
PIM inhibitor	5.3	0.64	3.7	0.75
JAK1/3 inhibitor	5	0.38	5.7	0.73
JAK2 inhibitor	3.8	0.69	4.3	0.49
JAK1 inhibitor	3.7	0.73	1.4	0.73
AKT inhibitor	3.3	0.57	2.7	0.87
ATR inhibitor	1	0.93	5.1	0.74

Supplemental Table 2: Identification of affected pathways through GSEA. Staudt gene signatures enrichment analysis using BROAD GSEA and the differential gene expression profiling. Below are the GSEA results comparing the AZD2014 signle agent, Ibrutinib single agent, combination to the vehicle for the OCI-Ly10 cells alone, TMD8 cells alone, and both lines together. A NES score greater than 1 was considered significantly enriched.

OCILY10 AZD2014 Versus Vehicle Top 15 Enriched Gene Sets with Up-Regulated Genes	SIZE	ES	NES	NOM p-val	FDR q-val	FWER p-val	RANK AT MAX
RIBOSOMAL_PROTEIN	19	0.71753	1.09051	0.34109	1	0.505	8155
PMBL_HL_HIGH	68	0.32757	0.9833	0.34731	1	1	8779
STROMAL-2_DLBCL_SURVIVAL_PREDICTOR	60	0.39744	0.8984	0.65669	1	1	4969
MYC_OVEREXPRESSION_2X_DOWN	17	0.41246	0.89196	0.66667	1	1	5788
P53_UP_XRAY	16	0.36482	0.7842	0.65669	1	1	8299
NFKB_UP_K1106	17	0.38696	0.72393	0.6705	1	1	8779
MYC_CHIP_PET_EXPR_DOWN	254	0.31197	0.69567	0.6705	1	1	10030
STAT3_UP_OCILY10	41	0.28994	0.65223	0.6705	1	1	9728
BLIMP_BCELL_REPRESSED	62	0.31245	0.649	0.6705	1	1	9589
NFKB_UP_BOTHOCILY3ANDLY10	37	0.35806	0.63684	0.6705	0.9904	1	10043
NFKB_UP_ALL_OCILY3_LY10	63	0.28881	0.62892	0.6705	0.916	1	10126
**OCILY10 AZD2014 Versus Vehicle Top 15 Enriched Gene Sets with Down-Regulated Genes**	**SIZE**	**ES**	**NES**	**NOM p-val**	**FDR q-val**	**FWER p-val**	**RANK AT MAX**
LEUCINE_STARVE_DOWN	174	-0.7722	-1.3075	0	1	0.323	10693
MYC_OVEREXPRESSION_1.5X_UP	83	-0.7534	-1.2713	0	0.90086	0.495	10867
GLUTAMINE_STARVE_DOWN	301	-0.7629	-1.2624	0	0.70578	0.495	6730
MYC_OVEREXPRESSION_2X_UP	33	-0.7158	-1.2485	0	0.60823	0.495	10867
IL6_LY10_UP_GROUP1	24	-0.7784	-1.2367	0	0.52099	0.495	5857
IL6_LY10_UP_ALL	38	-0.7124	-1.2322	0	0.46282	0.495	5857
IRF4_TARGET_MM_B_CELL_ACTIVATION	77	-0.7274	-1.2276	0	0.42128	0.495	6833
CELL_CYCLE_CHO_WHITFIELD	92	-0.7218	-1.2211	0	0.40807	0.495	11155
PROLIFERATION_NODE1618	57	-0.837	-1.2164	0	0.3978	0.495	6772
CELL_CYCLE_LIU	227	-0.7097	-1.2083	0	0.37522	0.495	11862
B_CELL_UP_ANERGY	17	-0.6589	-1.1916	0	0.39647	0.495	5880
HIF1ALPHA_2X_UP	64	-0.5171	-1.1874	0	0.37776	0.495	9094
HIF1ALPHA_2X_DOWN	40	-0.7435	-1.1856	0	0.37322	0.495	4821
MYC_CHIP_PET_EXPR_UP	382	-0.7448	-1.1833	0	0.35885	0.495	11406
NFKB_UP_OCILY10_ONLY	16	-0.5557	-1.1512	0	0.40466	0.495	5266

OCILY10 Ibrutinib Versus Vehicle Top 15 Enriched Gene Sets with Up-Regulated Genes	SIZE	ES	NES	NOM p-val	FDR q-val	FWER p-val	RANK AT MAX
RIBOSOMAL_PROTEIN	19	0.51035	0.84998	0.34531	1	0.69	7570
MYC_OVEREXPRESSION_2X_DOWN	17	0.35868	0.72115	0.69556	1	0.841	1232
CD40_DOWNREGULATED_BURKITT_LYMPOMA	69	0.32267	0.69871	0.69556	0.97706	0.841	3111
	**SIZE**	**ES**	**NES**	**NOM p-val**	**FDR q-val**	**FWER p-val**	**RANK AT MAX**
NFKB_UP_BOTHOCILY3ANDLY10	37	-0.8457	-1.3351	0	1	0.327	5454
IL6_LY10_UP_ALL	38	-0.8179	-1.3284	0	1	0.478	3746
PROLIFERATION_NODE1618	57	-0.8125	-1.3169	0	1	0.478	9493
NFKB_UP_ALL_OCILY3_LY10	63	-0.8069	-1.3028	0	1	0.478	5753
NFKB_UP_BCR_PAPER	63	-0.8117	-1.2789	0	1	0.478	4243
IL6_LY10_UP_GROUP1	24	-0.8497	-1.2756	0	0.93663	0.478	3746
STAT3HIGH_ABC_DLBCL_SUBGROUP	92	-0.7121	-1.2704	0	0.82904	0.478	13058
NFKB_UP_K1106	17	-0.8846	-1.2528	0	0.74835	0.478	3899
GLUCOSE_STARVE_UP	40	-0.6544	-1.2472	0	0.68558	0.478	13078
LEUCINE_STARVE_DOWN	174	-0.7366	-1.2368	0	0.63537	0.478	13269
MYC_OVEREXPRESSION_1.5X_UP	83	-0.7795	-1.2351	0	0.59429	0.478	8718
DC_TLR4_TLR8_SYNERGY	31	-0.631	-1.2277	0	0.56005	0.478	6651
GLUTAMINE_STARVE_DOWN	301	-0.7367	-1.2252	0	0.53109	0.478	10145
MYC_CHIP_PET_EXPR_UP	382	-0.743	-1.213	0	0.50626	0.478	12300
JAK_IL10_LY10_UP	38	-0.7224	-1.2	0	0.48474	0.478	4093

OCILY10 Combination Versus Vehicle Top 15 Enriched Gene Sets with Up-Regulated Genes	SIZE	ES	NES	NOM p-val	FDR q-val	FWER p-val	RANK AT MAX
MYC_OVEREXPRESSION_2X_DOWN	17	0.7147	1.4028	0	0.29913	0.162	1554
CD40_DOWNREGULATED_BURKITT_LYMPOMA	69	0.6388	1.2516	0	0.75648	0.508	6709
BLIMP_BCELL_REPRESSED	62	0.5621	1.0823	0.33595	1	0.835	11278
MYC_CHIP_PET_EXPR_DOWN	254	0.4478	0.9951	0.33529	1	0.835	12573
PAX5_REPRESSED	67	0.3939	0.9565	0.33529	1	0.835	11155

RIBOSOMAL_PROTEIN	19	0.6673	0.9175	0.32024	1	0.835	11063
STROMAL-2_DLBCL_SURVIVAL_PREDICTOR	60	0.3836	0.8745	0.65686	0.98154	0.835	10676
P53_UP_XRAY	16	0.5057	0.8674	0.65686	0.88504	0.835	5386
GLUTAMINE_STARVE_UP	283	0.3751	0.8649	0.33529	0.80999	0.835	11843
HRAS_OVEREXPRESSION_2X_DOWN	68	0.4242	0.8538	0.65686	0.75443	0.835	10959
STROMAL-1_DLBCL_SURVIVAL_PREDICTOR	242	0.3807	0.8462	0.65686	0.71388	0.835	8191
HIF1ALPHA_2X_UP	64	0.3652	0.7558	0.65686	0.77061	0.835	6586
**OCILY10 Combination Versus Vehicle Top 15 Enriched Gene Sets with Down-Regulated Genes**	**SIZE**	**ES**	**NES**	**NOM p-val**	**FDR q-val**	**FWER p-val**	**RANK AT MAX**
HIF1ALPHA_2X_DOWN	40	-0.8116	-1.4310	0	0.2111	0	4204
LEUCINE_STARVE_DOWN	174	-0.7956	-1.4153	0	0.2111	0.175	5153
NFKB_UP_K1106	17	-0.8010	-1.3849	0	0.49337	0.337	5631
IL6_LY10_UP_ALL	38	-0.8408	-1.3742	0	0.4228	0.337	3386
IRF4_TARGET_MM_B_CELL_ACTIVATION	77	-0.7617	-1.3667	0	0.38046	0.337	7225
GLUTAMINE_STARVE_DOWN	301	-0.7842	-1.3583	0	0.35223	0.337	7249
NFKB_UP_ALL_OCILY3_LY10	63	-0.8192	-1.3422	0	0.33207	0.337	6091
NFKB_UP_OCILY10_ONLY	16	-0.8481	-1.3412	0	0.31695	0.337	2659
MYC_OVEREXPRESSION_1.5X_UP	83	-0.8512	-1.3407	0	0.30519	0.337	5962
MYC_RNAI_OCILY3	54	-0.8100	-1.3345	0	0.32812	0.502	4495
MYC_CHIP_PET_EXPR_UP	382	-0.7915	-1.3342	0	0.31748	0.502	7261
NFKB_UP_HBL1	200	-0.6825	-1.3296	0	0.30862	0.502	6986
CELL_CYCLE_CHO_WHITFIELD	92	-0.7358	-1.3229	0	0.30112	0.502	6493
PROLIFERATION_NODE1618	57	-0.8477	-1.3216	0	0.29469	0.502	5770
CELL_CYCLE_LIU	227	-0.7119	-1.3187	0	0.28911	0.502	7213
**OCILY10 Combination Versus Vehicle Top Enriched STAT Gene Sets with Down-Regulated Genes**	**SIZE**	**ES**	**NES**	**NOM p-val**	**FDR q-val**	**FWER p-val**	**RANK AT MAX**
STAT3HIGH_ABC_DLBCL_SUBGROUP	92	-0.6604	-1.1459	0	0.42445	0.829	7266
STAT3_UP_OCILY10	41	-0.5489	-1.0557	0.32738	0.48189	0.829	4305
**OCILY10 Combination Versus Vehicle Top Enriched NFKB Gene Sets with Down-Regulated Genes**	**SIZE**	**ES**	**NES**	**NOM p-val**	**FDR q-val**	**FWER p-val**	**RANK AT MAX**
NFKB_UP_K1106	17	-0.8010	-1.3849	0	0.49337	0.337	5631
NFKB_UP_ALL_OCILY3_LY10	63	-0.8192	-1.3422	0	0.33207	0.337	6091
NFKB_UP_OCILY10_ONLY	16	-0.8481	-1.3412	0	0.31695	0.337	2659
NFKB_UP_HBL1	200	-0.6825	-1.3296	0	0.30862	0.502	6986
NFKB_UP_BCR_PAPER	63	-0.7196	-1.3048	0	0.27611	0.502	6209
NFKB_UP_BOTHOCILY3ANDLY10	37	-0.8263	-1.2393	0	0.32692	0.502	6091
NFKB_CHIPCHIP_YOUNG_4FACTORS	35	-0.6275	-1.1098	0	0.44573	0.829	10905
NFKB_CHIPCHIP_YOUNG_5FACTORS	15	-0.6263	-1.0823	0.32803	0.48365	0.829	10624
NFKB_CHIPCHIP_YOUNG_ALL	328	-0.4523	-0.9975	0.32803	0.54557	0.829	7043
**OCILY10 Combination Versus Vehicle Top Enriched MYC Gene Sets with Down-Regulated Genes**	**SIZE**	**ES**	**NES**	**NOM p-val**	**FDR q-val**	**FWER p-val**	**RANK AT MAX**
MYC_OVEREXPRESSION_1.5X_UP	83	-0.8512	-1.3407	0	0.30519	0.337	5962
MYC_RNAI_OCILY3	54	-0.8100	-1.3345	0	0.32812	0.502	4495
MYC_CHIP_PET_EXPR_UP	382	-0.7915	-1.3342	0	0.31748	0.502	7261
MYC_OVEREXPRESSION_2X_UP	33	-0.8407	-1.3076	0	0.27993	0.502	6531
MYC_CHIP_PET_3PLUS	423	-0.3956	-0.9609	0.32803	0.5486	0.829	7213

TMD8 AZD2014 Versus Vehicle Top 15 Enriched Gene Sets with Up-Regulated Genes	SIZE	ES	NES	NOM p-val	FDR q-val	FWER p-val	RANK AT MAX
NFKB_UP_BOTHOCILY3ANDLY10	37	0.7351	1.1548	0	1	0.321	13294
B_CELL_UP_ANERGY	17	0.6576	1.1404	0.3419	1	0.506	14159
PROLIFERATION_NODE1542	30	0.7030	1.1315	0	1	0.506	15624
CD40_UPREGULATED_BURKITT_LYMPHOMA	99	0.6487	1.1202	0	1	0.506	9042
BLIMP_BCELL_REPRESSED	62	0.6851	1.1158	0	1	0.506	11624
E2F3_OVEREXPRESSION_2X_UP	128	0.6366	1.1079	0	1	0.506	13866
MYC_OVEREXPRESSION_2X_DOWN	17	0.5937	1.1025	0.31801	1	0.506	13299
E2F3_OVEREXPRESSION_4X_UP	40	0.6471	1.0992	0	1	0.506	11695
PROLIFERATION_NODE1606	37	0.7053	1.0891	0	1	0.692	13438
GLUCOSE_STARVE_UP	40	0.6139	1.0725	0.3419	1	0.692	13924
JAK_IL10_LY10_UP	38	0.6276	1.0665	0.3419	1	0.692	13440
PAX5_REPRESSED	67	0.5013	1.0621	0.31801	1	0.692	13440
GLUTAMINE_STARVE_UP	283	0.6687	1.0616	0.3419	1	0.692	13555

RIBOSOMAL_PROTEIN	19	0.8196	1.0594	0.34191	1	0.692	6689
MYC_OVEREXPRESSION_2X_UP	33	0.6537	1.0506	0.3419	1	0.692	15431
**TMD8 AZD2014 Versus Vehicle Top 15 Enriched Gene Sets with Down-Regulated Genes**	**SIZE**	**ES**	**NES**	**NOM p-val**	**FDR q-val**	**FWER p-val**	**RANK AT MAX**
STROMAL-2_DLBCL_SURVIVAL_PREDICTOR	60	-0.5380	-1.0081	0.36328	1	0.642	5217
IL6_LY10_UP_GROUP1	24	-0.3632	-0.7883	0.63867	1	0.827	4660
IL6_LY10_UP_ALL	38	-0.3525	-0.7443	0.63867	1	0.827	4660
NFKB_UP_OCILY10_ONLY	16	-0.3768	-0.7253	0.63867	0.92586	0.827	5625

TMD8 Ibrutinib Versus Vehicle Top 15 Enriched Gene Sets with Up-Regulated Genes	SIZE	ES	NES	NOM p-val	FDR q-val	FWER p-val	RANK AT MAX
CD40_DOWNREGULATED_BURKITT_LYMPOMA	69	0.6884	1.2295	0	1	0.329	8192
BLIMP_BCELL_REPRESSED	62	0.7102	1.2036	0	1	0.651	12384
PAX5_REPRESSED	67	0.6126	1.1992	0	1	0.651	13658
GLUTAMINE_STARVE_UP	283	0.6660	1.0821	0.3556	1	0.834	12267
MYC_CHIP_PET_EXPR_DOWN	254	0.7039	1.0567	0	1	0.834	12177
LEUCINE_STARVE_UP	123	0.5974	1.0544	0.3556	1	0.834	10146
HRAS_OVEREXPRESSION_2X_DOWN	68	0.6825	1.0481	0	1	0.834	12384
P53_UP_XRAY	16	0.6360	1.0093	0.3556	1	0.834	8584
XBP1_TARGET_ALL	75	0.5777	0.9984	0.3556	1	0.834	16511
HIF1ALPHA_2X_UP	64	0.5625	0.9865	0.3556	1	0.834	9666
BCL6_CHIP-CHIP_3CELLTYPES_BCL6_MOTIF_CI	374	0.5388	0.9763	0.3556	1	0.834	12408
BCL6_TARGETS_CHIPCHIP	426	0.5706	0.9660	0.3556	1	0.834	12365
NFKB_CHIPCHIP_YOUNG_5FACTORS	15	0.5313	0.9633	0.3556	1	0.834	8926
MYC_OVEREXPRESSION_2X_DOWN	17	0.4690	0.9478	0.3556	1	0.834	8030
STROMAL-1_DLBCL_SURVIVAL_PREDICTOR	242	0.5182	0.9462	0.71513	1	0.834	12539
**TMD8 Ibrutinib Versus Vehicle Top 15 Enriched Gene Sets with Down-Regulated Genes**	**SIZE**	**ES**	**NES**	**NOM p-val**	**FDR q-val**	**FWER p-val**	**RANK AT MAX**
NFKB_UP_K1106	17	-0.7108	-1.2210	0	1	0.349	981
PROLIFERATION_NODE1618	57	-0.8339	-1.2074	0	1	0.494	2708
IL6_LY10_UP_GROUP1	24	-0.7118	-1.2007	0	1	0.494	2525
NFKB_UP_BOTHOCILY3ANDLY10	37	-0.7130	-1.1853	0	1	0.819	981
IL6_LY10_UP_ALL	38	-0.6672	-1.1083	0	1	0.819	2525
NFKB_UP_ALL_OCILY3_LY10	63	-0.6561	-1.1082	0	1	0.819	981
NFKB_UP_BCR_PAPER	63	-0.6137	-1.0339	0	1	0.819	1063
MYC_OVEREXPRESSION_1.5X_UP	83	-0.6321	-1.0161	0	1	0.819	6669
MYC_OVEREXPRESSION_2X_UP	33	-0.6193	-0.9761	0.6841	1	0.819	6301
CELL_CYCLE_CHO_WHITFIELD	92	-0.5644	-0.9336	0.6841	1	0.819	2612
HIF1ALPHA_2X_DOWN	40	-0.5912	-0.8915	0.6841	1	0.819	2742
NFKB_UP_OCILY10_ONLY	16	-0.5175	-0.8828	0.6841	1	0.819	795
BCL6_REPRESSED	19	-0.3826	-0.8184	0.6841	1	0.819	795
MYC_CHIP_PET_EXPR_UP	382	-0.4478	-0.8147	0.6841	1	0.819	5934
STROMAL-2_DLBCL_SURVIVAL_PREDICTOR	60	-0.3673	-0.8006	0.6841	1	0.819	5888

TMD8 Combination Versus Vehicle Top 15 Enriched Gene Sets with Up-Regulated Genes	SIZE	ES	NES	NOM p-val	FDR q-val	FWER p-val	RANK AT MAX
BLIMP_BCELL_REPRESSED	62	0.7251	1.1295	0	1	0.674	10865
CD40_DOWNREGULATED_BURKITT_LYMPOMA	69	0.6263	1.0991	0	1	0.833	10420
RIBOSOMAL_PROTEIN	19	0.7070	1.0589	0.34746	1	0.833	16066
PAX5_REPRESSED	67	0.5433	1.0532	0	1	0.833	9549
GLUTAMINE_STARVE_UP	283	0.6797	1.0345	0	1	0.833	11595
HRAS_OVEREXPRESSION_2X_DOWN	68	0.6732	1.0328	0.38028	1	0.833	11451
MYC_CHIP_PET_EXPR_DOWN	254	0.6829	1.0148	0	1	0.833	11641
LEUCINE_STARVE_UP	123	0.5982	0.9889	0.38028	1	0.833	10987
MYC_OVEREXPRESSION_2X_DOWN	17	0.5219	0.9719	0.38028	1	0.833	5960
HIF1ALPHA_2X_UP	64	0.5162	0.9483	0.38028	1	0.833	7091
XBP1_TARGET_ALL	75	0.5599	0.9152	0.68008	1	0.833	16492
P53_UP_XRAY	16	0.5993	0.9057	0.68008	1	0.833	10076
KRAS_UP	82	0.5211	0.8962	0.68008	1	1	8430
STROMAL-1_DLBCL_SURVIVAL_PREDICTOR	242	0.4311	0.8884	0.38028	1	1	13165
BCL6_TARGETS_CHIPCHIP	426	0.5270	0.8737	0.68008	1	1	11631
**TMD8 Combination Versus Vehicle Top Enriched NFKB Gene Sets with Up-Regulated Genes**	**SIZE**	**ES**	**NES**	**NOM p-val**	**FDR q-val**	**FWER p-val**	**RANK AT MAX**
NFKB_CHIPCHIP_YOUNG_4FACTORS	35	0.4304	0.7673	0.68008	1	1	8156
NFKB_CHIPCHIP_YOUNG_5FACTORS	15	0.4391	0.7660	0.68008	1	1	8156
NFKB_CHIPCHIP_YOUNG_ALL	328	0.4071	0.7631	0.68008	1	1	11608

TMD8 Combination Versus Vehicle Top Enriched MYC Gene Sets with Up-Regulated Genes	SIZE	ES	NES	NOM p-val	FDR q-val	FWER p-val	RANK AT MAX
MYC_CHIP_PET_EXPR_DOWN	254	0.6829	1.0148	0	1	0.833	11641
MYC_OVEREXPRESSION_2X_DOWN	17	0.5219	0.9719	0.38028	1	0.833	5960
MYC_CHIP_PET_3PLUS	423	0.4693	0.8319	0.68008	1	1	11679
**TMD8 Combination Versus Vehicle Top 15 Enriched Gene Sets with Down-Regulated Genes**	**SIZE**	**ES**	**NES**	**NOM p-val**	**FDR q-val**	**FWER p-val**	**RANK AT MAX**
PROLIFERATION_NODE1618	57	-0.8847	-1.2100	0	1	0.331	3399
IL6_LY10_UP_GROUP1	24	-0.7462	-1.1754	0	0.89963	0.49	1189
NFKB_UP_K1106	17	-0.6860	-1.1435	0	1	0.679	1983
NFKB_UP_ALL_OCILY3_LY10	63	-0.6885	-1.1404	0	1	0.679	4831
NFKB_UP_BCR_PAPER	63	-0.6972	-1.1228	0	1	0.679	3496
IL6_LY10_UP_ALL	38	-0.7312	-1.1193	0	1	0.851	1189
NFKB_UP_BOTHOCILY3ANDLY10	37	-0.7083	-1.1102	0	1	0.851	3412
MYC_OVEREXPRESSION_1.5X_UP	83	-0.7365	-1.1036	0	1	0.851	6465
NFKB_UP_OCILY10_ONLY	16	-0.6300	-1.0416	0.33469	1	0.851	4757
CELL_CYCLE_CHO_WHITFIELD	92	-0.6446	-1.0087	0.34082	1	0.851	3334
HIF1ALPHA_2X_DOWN	40	-0.6802	-0.9956	0.34082	1	0.851	5104
MYC_OVEREXPRESSION_2X_UP	33	-0.6359	-0.9808	0.67551	1	0.851	5026
B_CELL_UP_ANERGY	17	-0.4862	-0.9804	0.34746	1	0.851	2529
LEUCINE_STARVE_DOWN	174	-0.5965	-0.9526	0.34082	1	0.851	6667
STROMAL-2_DLBCL_SURVIVAL_PREDICTOR	60	-0.4572	-0.9473	0.33469	1	0.851	6672
**TMD8 Combination Versus Vehicle Top Enriched STAT Gene Sets with Down-Regulated Genes**	**SIZE**	**ES**	**NES**	**NOM p-val**	**FDR q-val**	**FWER p-val**	**RANK AT MAX**
STAT3HIGH_ABC_DLBCL_SUBGROUP	92	-0.4681	-0.8253	0.67551	1	1	3410
STAT3_UP_OCILY10	41	-0.4528	-0.8250	0.67551	1	1	1702
**TMD8 Combination Versus Vehicle Top Enriched NFKB Gene Sets with Down-Regulated Genes**	**SIZE**	**ES**	**NES**	**NOM p-val**	**FDR q-val**	**FWER p-val**	**RANK AT MAX**
NFKB_UP_K1106	17	-0.6860	-1.1435	0	1	0.679	1983
NFKB_UP_ALL_OCILY3_LY10	63	-0.6885	-1.1404	0	1	0.679	4831
NFKB_UP_BCR_PAPER	63	-0.6972	-1.1228	0	1	0.679	3496
NFKB_UP_BOTHOCILY3ANDLY10	37	-0.7083	-1.1102	0	1	0.851	3412
NFKB_UP_OCILY10_ONLY	16	-0.6300	-1.0416	0.33469	1	0.851	4757
NFKB_UP_HBL1	200	-0.5100	-0.8835	0.67551	1	1	3505
**TMD8 Combination Versus Vehicle Top Enriched MYC Gene Sets with Down-Regulated Genes**	**SIZE**	**ES**	**NES**	**NOM p-val**	**FDR q-val**	**FWER p-val**	**RANK AT MAX**
MYC_OVEREXPRESSION_1.5X_UP	83	-0.7365	-1.1036	0	1	0.851	6465
MYC_OVEREXPRESSION_2X_UP	33	-0.6359	-0.9808	0.67551	1	0.851	5026
MYC_CHIP_PET_EXPR_UP	382	-0.5742	-0.9260	0.67551	1	0.851	7152
MYC_RNAI_OCILY3	54	-0.6063	-0.9121	0.67551	1	0.851	7040

Both Lines Combination Versus Vehicle Top 15 Enriched Gene Sets with Up-Regulated Genes	SIZE	ES	NES	NOM p-val	FDR q-val	FWER p-val	RANK AT MAX
BLIMP_BCELL_REPRESSED	62	0.7043	1.4063	0.01815	0.33623	0.146	8920
CD40_DOWNREGULATED_BURKITT_LYMPOMA	69	0.6613	1.3807	0.03734	0.23267	0.257	8152
RIBOSOMAL_PROTEIN	19	0.6908	1.2085	0.26465	0.74597	0.653	15434
PAX5_REPRESSED	67	0.4922	1.1984	0.11042	0.59363	0.666	7669
HRAS_OVEREXPRESSION_2X_DOWN	68	0.5916	1.1911	0.15114	0.49817	0.702	11338
MYC_CHIP_PET_EXPR_DOWN	254	0.6299	1.1760	0.14669	0.45088	0.717	10601
MYC_OVEREXPRESSION_2X_DOWN	17	0.6088	1.1387	0.26305	0.4903	0.771	8663
GLUTAMINE_STARVE_UP	283	0.5769	1.1256	0.25826	0.4554	0.771	10667
P53_UP_XRAY	16	0.5655	1.0043	0.52311	0.71479	0.867	8249
STROMAL-1_DLBCL_SURVIVAL_PREDICTOR	242	0.4230	0.9653	0.62603	0.74727	0.885	11145
KRAS_UP	82	0.4296	0.9529	0.47718	0.69199	0.885	5593
HIF1ALPHA_2X_UP	64	0.4421	0.9508	0.56729	0.64071	0.885	7745
LEUCINE_STARVE_UP	123	0.4580	0.9423	0.64256	0.60375	0.885	9022
XBP1_TARGET_ALL	75	0.3966	0.7519	0.81497	0.8569	0.944	13192
BCL6_TARGETS_CHIPCHIP	426	0.3454	0.7195	0.80372	0.84521	0.944	11334

Both Lines Combination Versus Vehicle Top Enriched MYC Gene Sets with Up-Regulated Genes	SIZE	ES	NES	NOM p-val	FDR q-val	FWER p-val	RANK AT MAX
MYC_CHIP_PET_EXPR_DOWN	254	0.6299	1.1760	0.14669	0.45088	0.717	10601
MYC_OVEREXPRESSION_2X_DOWN	17	0.6088	1.1387	0.26305	0.4903	0.771	8663
MYC_CHIP_PET_3PLUS	423	0.3185	0.6815	0.86515	0.8385	0.944	10716

Both Lines Combination Versus Vehicle Top 15 Enriched Gene Sets with Down-Regulated Genes	SIZE	ES	NES	NOM p-val	FDR q-val	FWER p-val	RANK AT MAX
MYC_OVEREXPRESSION_1.5X_UP	83	-0.8511	-1.4221	0	1	0.144	4049
MYC_CHIP_PET_EXPR_UP	382	-0.7598	-1.4134	0.02677	0.60725	0.174	7084
MYC_OVEREXPRESSION_2X_UP	33	-0.8000	-1.4070	0.02672	0.40962	0.174	4049
NFKB_UP_BCR_PAPER	63	-0.7433	-1.3870	0.01734	0.34982	0.199	7050
IRF4_TARGET_MM_B_CELL_ACTIVATION	77	-0.7252	-1.3774	0.02697	0.30719	0.245	5966
GLUTAMINE_STARVE_DOWN	301	-0.7420	-1.3744	0.04449	0.27045	0.258	5528
IL6_LY10_UP_ALL	38	-0.8338	-1.3693	0.02724	0.24754	0.258	1332
LEUCINE_STARVE_DOWN	174	-0.7528	-1.3662	0.02672	0.21839	0.258	5281
PROLIFERATION_NODE1618	57	-0.8942	-1.3637	0	0.20476	0.298	3037
MYC_RNAI_OCILY3	54	-0.7720	-1.3542	0.06049	0.21036	0.349	4787
NFKB_UP_ALL_OCILY3_LY10	63	-0.7792	-1.3500	0.02708	0.19764	0.368	3090
BLIMP_PROLIFERATION_REPRESSED	87	-0.6847	-1.3457	0.04406	0.18827	0.398	5897
NFKB_UP_HBL1	200	-0.6289	-1.3280	0.04406	0.21942	0.451	7116
STAT3HIGH_ABC_DLBCL_SUBGROUP	92	-0.6070	-1.3204	0.09867	0.21705	0.465	4771
HIF1ALPHA_2X_DOWN	40	-0.8034	-1.3199	0.06049	0.20422	0.465	2941

Both Lines Combination Versus Vehicle Top Enriched STAT Gene Sets with Down-Regulated Genes	SIZE	ES	NES	NOM p-val	FDR q-val	FWER p-val	RANK AT MAX
STAT3HIGH_ABC_DLBCL_SUBGROUP	92	-0.6070	-1.3204	0.09867	0.21705	0.465	4771
STAT3_UP_OCILY10	41	-0.5501	-1.1768	0.24953	0.36295	0.713	1121

Both Lines Combination Versus Vehicle Top Enriched NFKB Gene Sets with Down-Regulated Genes	SIZE	ES	NES	NOM p-val	FDR q-val	FWER p-val	RANK AT MAX
NFKB_UP_BCR_PAPER	63	-0.7433	-1.3870	0.01734	0.34982	0.199	7050
NFKB_UP_ALL_OCILY3_LY10	63	-0.7792	-1.3500	0.02708	0.19764	0.368	3090
NFKB_UP_HBL1	200	-0.6289	-1.3280	0.04406	0.21942	0.451	7116
NFKB_UP_K1106	17	-0.7856	-1.2987	0.09478	0.19955	0.492	3957
NFKB_UP_BOTHOCILY3ANDLY10	37	-0.7828	-1.2706	0.09515	0.22674	0.569	6242
NFKB_UP_OCILY10_ONLY	16	-0.8119	-1.2066	0.08946	0.32916	0.698	2667
NFKB_CHIPCHIP_YOUNG_4FACTORS	35	-0.4458	-0.8336	0.74757	0.80206	0.952	4857
NFKB_CHIPCHIP_YOUNG_5FACTORS	15	-0.4271	-0.7540	0.81563	0.83847	0.952	4167
NFKB_CHIPCHIP_YOUNG_ALL	328	-0.3152	-0.6876	0.93064	0.86977	0.952	4550

Both Lines Combination Versus Vehicle Top Enriched MYC Gene Sets with Down-Regulated Genes	SIZE	ES	NES	NOM p-val	FDR q-val	FWER p-val	RANK AT MAX
MYC_OVEREXPRESSION_1.5X_UP	83	-0.8511	-1.4221	0	1	0.144	4049
MYC_CHIP_PET_EXPR_UP	382	-0.7598	-1.4134	0.02677	0.60725	0.174	7084
MYC_OVEREXPRESSION_2X_UP	33	-0.8000	-1.4070	0.02672	0.40962	0.174	4049
MYC_RNAI_OCILY3	54	-0.7720	-1.3542	0.06049	0.21036	0.349	4787

Supplemental Table 3: NF-kB target genes regulated by treatments. Overlapping genes from the Staudt NFKB gene signatures and their expression from OCI-LY10 and TMD8 DLBCL cell lines 24 hours post-treatment with either AZD2014, Ibrutinib, or combination. The count summarized RNAseq data was first normalized using trimmed mean of M-values (TMM) using the R package edgeR. Colum heading abbreviations include the following: LY10 for OCI-Ly10, A for AZD2014, C for combination, I for Ibrutinib, V for vehicle, and a or b for identifying the experimental replicates.

Ensembl Gene ID	Gene Symbol	LY10 24HR A a Log2 (TMM Counts+ 1)	LY10 24HR A b Log2 (TMM Counts+ 1)	LY10 24HR C a Log2 (TMM Counts+ 1)	LY10 24HR C b Log2 (TMM Counts+ 1)	LY10 24HR I a Log2 (TMM Counts+ 1)	LY10 24HR I b Log2 (TMM Counts+ 1)	LY10 24HR V a Log2 (TMM Counts+ 1)	LY10 24HR V b Log2 (TMM Counts+ 1)	TMD8 24HR A a Log2 (TMM Counts+ 1)	TMD8 24HR A b Log2 (TMM Counts+ 1)	TMD8 24HR C a Log2 (TMM Counts+ 1)	TMD8 24HR C b Log2 (TMM Counts+ 1)	TMD8 24HR I a Log2 (TMM Counts+ 1)	TMD8 24HR I b Log2 (TMM Counts+ 1)	TMD8 24HR V a Log2 (TMM Counts+ 1)	TMD8 24HR V b Log2 (TMM Counts+ 1)
ENSG00000127220	ABHD8	5.2731	4.7192	5.5429	4.9782	4.9841	4.0909	5.8664	4.1922	6.1767	7.1842	6.3764	6.9557	7.1116	6.4211	5.5895	6.805
ENSG00000166016	ABTB2	6.4077	6.9572	4.1506	6.5141	2.9373	6.3438	6.8775	6.5859	6.992	7.585	5.0725	6.04	5.7104	5.1239	6.0566	7.7986
ENSG00000115091	ACTR3	12.8906	12.7094	12.691	13.0518	11.4907	13.0284	13.2364	13.0078	12.7226	13.0485	12.9885	13.0188	13.5633	12.7014	12.1502	13.2417
ENSG00000140955	ADAD2	0	0	0	0	0	1.1043	0	0	0.9709	0	0	0	0	0	0	1.6462
ENSG00000151651	ADAM8	11.5329	10.7691	11.1406	10.744	9.2886	9.9762	11.1293	9.9409	9.1122	9.7596	9.0186	9.679	9.0933	8.3938	7.3815	8.3202
ENSG00000119844	AFTPH	11.3058	11.4025	11.0755	11.805	9.639	11.5089	11.4383	11.2943	10.6197	10.9048	10.4523	10.8253	11.2842	10.3906	9.5109	10.9141
ENSG00000106546	AHR	1.8359	0	0.9486	0	0.7398	0	2.5311	0	10.074	10.1646	8.1806	8.6305	9.2791	8.5056	9.7244	11.5325
ENSG00000163568	AIM2	11.0573	10.0193	11.1062	10.7558	9.6653	10.4084	10.6496	9.8082	10.4659	10.6035	11.6524	11.6233	11.7874	10.7554	9.6404	10.0457
ENSG00000023330	ALAS1	9.1491	9.3837	8.9276	9.7089	7.9002	9.7625	9.9218	9.8425	9.3861	9.9976	9.6001	10.0997	10.472	9.7693	8.7523	10.1387
ENSG00000139223	ANP32D	0	1.7225	0.9486	0	0	0	0	1.6599	1.9598	0	0	1.6599	0.9928	1.0909	1.3045	0
ENSG00000182287	AP1S2	9.3705	9.5679	9.4341	9.9676	8.5755	9.6037	9.3817	9.3732	9.2327	9.815	10.0745	10.1595	10.5699	9.6927	8.9064	9.46
ENSG00000077420	APBB1IP	10.7844	10.556	10.9568	11.2086	10.0314	10.825	10.6928	9.8909	9.7384	10.1406	10.967	11.3218	11.7232	10.7778	8.5259	9.2
ENSG00000163697	APBB2	7.6161	7.8542	7.3812	7.6052	6.5993	7.8962	8.5893	8.5664	5.855	6.4694	4.8099	4.7506	5.4419	4.6915	5.756	6.8323
ENSG00000165269	AQP7	0	0	0	0	0	1.1043	0	0	0	0	0	0	0	0	0	0
ENSG00000103569	AQP9	1.4383	3.3533	0	2.7803	1.585	3.1731	1.9523	1.6599	0	0	0	0	0	0	0	0
ENSG00000134287	ARF3	11.2415	11.0943	10.8157	11.3293	10.2484	11.3558	11.7674	11.4556	10.703	11.3258	10.5799	11.0337	11.5694	10.8876	10.0045	11.2552
ENSG00000089820	ARHGAP4	10.4466	9.5854	10.4301	9.8422	9.2793	9.5951	10.4834	9.2197	9.4708	10.2172	9.8668	10.0983	10.1114	9.5413	7.905	9.3251
ENSG00000116584	ARHGEF2	10.8101	11.0456	11.0686	11.637	9.8906	11.4356	11.047	10.9962	10.2893	10.9226	10.7607	11.5546	11.5922	11.0424	9.0274	10.7444
ENSG00000120805	ARL1	10.4572	10.0145	9.9316	10.2811	9.2112	10.2773	10.9268	10.4732	9.4767	9.753	9.6512	9.6482	10.1024	9.2166	9.1855	9.8762
ENSG00000144746	ARL6IP5	9.7633	9.2427	9.9535	10.0353	9.2575	9.9941	10.0754	9.2249	9.7849	10.255	10.8854	10.5979	11.167	10.2757	9.6811	10.3746
ENSG00000139352	ASCL1	0	1.1043	0	0	0	0	0	0	0	0	0	0	0	0	0	0
ENSG00000161944	ASGR2	0	0	0	1.1177	0	0	0	0	0	0	0	0	0	0	0	0
ENSG00000116539	ASH1L	8.8152	9.8056	8.9897	10.4062	6.3859	9.986	9.2292	9.4709	8.8751	8.5359	8.9977	10.5928	10.472	10.0315	6.5632	9.6111
ENSG00000118217	ATF6	8.9004	9.0833	8.7203	9.3808	7.445	9.2159	9.3755	9.2456	9.207	9.3629	9.5363	9.8659	10.2025	9.3965	8.2538	9.5632
ENSG00000159199	ATP5G1	10.0946	9.351	8.4555	8.7544	10.2836	9.7395	11.2871	10.589	9.9971	11.8373	9.607	9.7781	10.3197	9.7842	11.0581	11.029
ENSG00000156966	B3GNT7	7.6595	7.8398	6.0481	7.6225	4.1961	7.102	8.2566	7.4325	6.755	7.5428	5.1502	6.5857	6.0909	6.15	5.4009	6.2401
ENSG00000176788	BASP1	11.6805	11.0369	10.9582	11.1078	10.1537	10.5319	11.7306	10.6485	10.6337	11.6629	10.5887	10.5918	10.7765	10.1205	10.353	10.7227
ENSG00000156127	BATF	11.9598	10.6948	9.6383	9.5257	9.4052	9.2325	12.246	11.174	10.4179	11.6855	8.5863	8.3444	8.783	7.9227	10.695	10.8564
ENSG00000123685	BATF3	6.755	5.9821	3.7115	4.9782	5.3568	5.0483	7.7677	6.9931	6.0976	7.4152	3.8135	3.269	3.306	2.1342	6.7325	6.4481
ENSG00000087088	BAX	11.5362	11.4012	11.256	11.9174	11.2435	11.8508	11.8745	11.7149	10.7253	12.3181	10.9589	11.0919	11.3846	10.762	10.675	11.1084
ENSG00000009954	BAZ1B	11.3595	11.599	10.8575	11.6675	9.0591	11.6829	12.1684	12.2332	10.4026	10.4327	9.8869	10.815	11.1651	10.5249	9.0577	11.5262
ENSG00000105327	BBC3	10.1726	9.6004	10.6542	10.708	8.8056	9.7375	9.6828	8.8184	8.0921	9.1846	9.7102	9.9502	9.1571	8.2244	7.0697	7.261
ENSG00000163093	BBS5	5.6381	5.5214	5.8053	6.0858	3.0583	5.3614	6.2392	5.7816	5.9704	6.4694	6.53	6.8784	6.8152	6.195	5.0622	6.8852
ENSG00000140379	BCL2A1	13.1346	11.6701	11.1937	10.454	11.0182	10.0134	12.5878	11.2868	11.5071	12.4972	10.8646	10.2506	10.8928	9.9416	11.6397	11.2765
ENSG00000171552	BCL2L1	10.3656	9.7136	8.6249	8.7661	6.7498	8.3407	9.7516	9.021	9.4006	9.6218	8.0188	8.3104	9.0536	8.0874	7.7158	9.0687
ENSG00000113916	BCL6	10.5985	10.7833	10.5675	11.1591	8.8911	10.2985	10.4502	9.8527	8.4027	9.4172	8.4467	9.787	9.0428	8.9264	7.3097	9.5591
ENSG00000023445	BIRC3	11.9225	11.5049	12.296	12.0008	10.0396	11.3852	11.9569	11.0756	9.3694	9.4189	10.2091	10.8466	10.8393	9.9299	7.9994	10.0774
ENSG00000113734	BNIP1	7.7426	7.0353	7.0674	7.3173	7.2313	7.5965	8.4064	7.6499	7.2421	8.2852	7.3348	7.3488	7.757	7.4221	7.234	7.8856
ENSG00000101425	BPI	0	0	0	0	0	0	0	0	0	0	0	0	0	0	0	0
ENSG00000133639	BTG1	13.6305	13.6606	13.4599	14.0254	12.5357	13.3489	12.7277	12.6193	12.0668	13.1272	12.7985	12.8758	12.8655	12.0152	11.3079	11.3959
ENSG00000165507	C10orf10	7.0254	6.9705	6.8517	7.4874	6.7136	6.8531	7.889	7.32	6.7806	7.4994	7.3513	7.5028	7.5596	6.9682	6.4338	7.7566
ENSG00000123395	C12orf44	8.1559	7.6459	7.5266	7.8814	5.044	7.8178	8.7062	8.0057	6.9811	6.8608	7.167	7.3296	7.6546	7.1397	5.7947	7.6225
ENSG00000169758	C15orf27	4.1102	3.7729	3.366	3.0072	2.6622	4.8334	3.5248	4.1922	4.1977	4.6253	3.6076	4.4983	4.2311	3.3262	2.2265	3.251
ENSG00000070761	C16orf80	8.6281	8.192	8.1545	8.445	7.7609	8.3458	9.2833	8.912	8.5472	9.9489	8.3917	8.41	8.7635	8.0137	8.2677	8.9369
ENSG00000212719	C17orf51	6.188	7.0851	5.0695	6.7047	3.969	6.8387	7.0847	7.3585	5.9923	6.0032	4.4276	5.6938	5.9823	5.6952	4.435	6.55

Ensembl Gene ID	Gene Symbol	LY10 24HR A a Log2 (TMM Counts+ 1)	LY10 24HR A b Log2 (TMM Counts+ 1)	LY10 24HR C a Log2 (TMM Counts+ 1)	LY10 24HR C b Log2 (TMM Counts+ 1)	LY10 24HR I a Log2 (TMM Counts+ 1)	LY10 24HR I b Log2 (TMM Counts+ 1)	LY10 24HR V a Log2 (TMM Counts+ 1)	LY10 24HR V b Log2 (TMM Counts+ 1)	TMD8 24HR A a Log2 (TMM Counts+ 1)	TMD8 24HR A b Log2 (TMM Counts+ 1)	TMD8 24HR C a Log2 (TMM Counts+ 1)	TMD8 24HR C b Log2 (TMM Counts+ 1)	TMD8 24HR I a Log2 (TMM Counts+ 1)	TMD8 24HR I b Log2 (TMM Counts+ 1)	TMD8 24HR V a Log2 (TMM Counts+ 1)	TMD8 24HR V b Log2 (TMM Counts+ 1)
ENSG00000159079	C21orf59	6.4786	6.2456	6.3243	6.5141	6.2153	6.0007	6.3947	5.7816	6.3572	7.3187	6.8311	7.3958	7.3659	6.5308	6.2645	6.749
ENSG00000162972	C2orf47	7.7135	7.8252	7.5046	8.2362	7.2817	8.0663	8.7895	8.4148	7.709	8.9118	8.0446	7.8249	8.3353	7.4596	8.1074	8.4707
ENSG00000125730	C3	5.3045	5.2858	5.3586	6.035	3.5435	5.7474	4.8909	5.0148	1.5509	2.7049	2.5008	4.0108	4.3052	4.8125	2.2265	4.7317
ENSG00000184530	C6orf58	0.8953	1.1043	0	1.1177	0	1.1043	0	1.6599	0	0	0	0	0	0	0	0
ENSG00000175600	C7orf10	0	1.7225	0	2.1763	1.585	2.4803	0	1.0566	0	0	0	1.6599	0.9928	0	0	0
ENSG00000122783	C7orf49	8.3838	8.4419	8.3768	8.9656	7.225	8.9207	8.9487	8.839	7.6479	8.1871	7.24	8.3539	8.9651	8.1689	6.911	8.5792
ENSG00000146540	C7orf50	6.6234	6.3913	6.6921	7.3796	6.2662	6.5696	6.9684	6.2998	7.6339	8.8952	7.7948	7.4855	8.0156	7.5229	7.6218	8.3628
ENSG00000063180	CA11	7.0627	6.3512	6.7681	7.241	6.7675	6.7646	7.7421	6.9177	6.2517	7.474	6.4856	6.754	6.8152	6.2813	5.8875	5.7623
ENSG00000042493	CAPG	9.1707	8.2202	9.9261	9.5142	9.3008	9.3763	9.0168	8.1984	10.2893	10.8261	11.0081	10.8305	11.6559	10.761	10.0712	10.5744
ENSG00000116489	CAPZA1	12.1805	11.7509	11.9048	12.2596	11.2264	12.1606	12.3725	12.0553	12.028	12.6913	12.4699	12.3773	12.9013	12.0704	11.8884	12.3381
ENSG00000166734	CASC4	8.8952	9.2563	9.1305	9.7934	7.6165	9.534	9.6281	9.1664	8.8602	9.3664	9.5489	9.9296	10.4251	9.5391	8.5056	9.5591
ENSG00000137812	CASC5	9.4731	9.3586	9.1684	9.3859	7.4228	9.4034	10.4244	9.9645	8.7739	8.7226	7.5865	8.2807	9.059	8.3193	7.7005	9.7062
ENSG00000121691	CAT	10.1801	10.1376	10.4705	10.7261	8.9699	10.6924	10.4433	9.9938	10.462	10.7515	10.9969	11.23	11.5266	10.5982	9.0007	10.4056
ENSG00000160050	CCDC28B	7.9162	7.0098	5.8985	6.5686	5.2087	6.0264	7.2924	5.9921	6.4072	6.9184	5.328	5.6005	6.0047	5.1699	5.8138	6.1564
ENSG00000115355	CCDC88A	11.5528	12.1315	10.7462	11.9576	9.1751	11.9918	11.9915	11.9828	10.4199	10.2661	10.1368	11.0862	11.5261	10.8996	8.8975	11.0414
ENSG00000108702	CCL1	0	0	0	0	0	0	0	0	0	0	0	0	0	0	0	0
ENSG00000102962	CCL22	10.7119	9.9918	5.1089	5.6185	4.2972	4.5801	10.1082	8.9313	7.3139	8.3874	1.5211	2.4114	2.3074	0	6.8662	5.8714
ENSG00000006075	CCL3	7.4281	7.5867	3.366	6.1348	4.7892	6.3234	9.8609	9.2327	0.9709	0	0	0	0	1.7049	1.9819	2.0704
ENSG00000129277	CCL4	6.3633	5.8728	2.4983	4.218	4.4803	4.0909	8.6036	8.8759	0	2.0566	0	2.0841	0	1.7049	2.6229	2.8856
ENSG00000161570	CCL5	3.121	5.5214	2.2388	5.5838	1.2203	4.8334	4.5223	4.8079	0.9709	0.84	0	0	0	1.7049	2.6229	2.0704
ENSG00000118971	CCND2	12.2795	11.7266	10.7265	10.6085	9.7015	10.3752	12.5738	11.9497	11.6108	12.5827	11.2124	11.8072	12.3364	11.6884	11.5634	12.0979
ENSG00000163660	CCNL1	9.7519	10.0911	9.7186	10.7391	7.8386	10.4633	10.0127	9.9831	10.4138	10.4269	10.6645	11.4697	11.5968	11.0049	9.0676	10.792
ENSG00000163823	CCR1	2.4005	3.7729	4.073	5.5113	3.969	5.8369	1.9523	4.275	2.281	0	4.153	4.6915	5.3402	4.9241	2.7845	4.4796
ENSG00000126353	CCR7	12.4518	12.3976	11.8996	12.5826	10.1487	12.0156	12.5611	12.0677	9.8676	10.2018	10.493	11.2242	10.6421	9.6033	7.234	8.0455
ENSG00000163468	CCT3	12.2206	11.8456	11.2416	11.6085	10.944	11.8499	13.1397	12.7253	12.4227	13.0524	11.8939	12.2678	12.7439	12.253	11.987	13.3078
ENSG00000012124	CD22	11.158	10.6541	11.0389	10.7361	9.543	10.4205	11.1705	9.8082	11.5779	12.4485	11.6207	13.0738	12.4287	12.2912	9.8438	11.5121
ENSG00000135218	CD36	10.9872	10.417	9.261	9.1823	7.2377	8.7119	9.843	9.9676	10.7618	11.1374	9.5668	9.3707	10.5671	9.2808	10.8969	12.0451
ENSG00000104894	CD37	13.1274	12.8965	13.7747	13.9637	12.0393	13.3775	13.0218	12.1961	13.2911	13.7801	14.1975	14.6961	14.4905	13.7938	11.5532	12.3767
ENSG00000101017	CD40	11.0894	10.5247	9.3967	9.8587	8.6005	9.5583	11.0906	10.3627	9.9641	11.0665	9.2594	10.0883	9.9736	9.6867	9.209	10.0044
ENSG00000026508	CD44	13.6767	13.4741	13.1269	13.5224	11.8887	12.5779	13.172	12.5954	13.0884	13.8715	13.0072	13.103	13.0438	12.01	12.4131	12.9748
ENSG00000196352	CD55	9.2958	8.9916	8.9359	9.4184	7.8259	9.1032	9.5289	8.9691	8.2328	8.2407	7.9056	8.2757	8.4457	7.487	7.2826	8.0513
ENSG00000116815	CD58	7.5843	7.1094	5.9208	6.5141	5.7274	6.8387	7.8296	7.2086	8.6684	9.6305	6.5871	7.041	7.4168	6.7735	8.4182	8.7135
ENSG00000110848	CD69	10.3287	9.982	9.4146	9.5371	7.5818	8.9842	9.5748	9.609	7.3313	7.1037	7.2489	7.041	7.1012	5.9269	5.4259	5.7336
ENSG00000121594	CD80	8.24	7.1214	5.0695	6.1821	3.9059	6.0007	8.0485	6.5859	8.6854	8.7441	7.0398	7.818	7.8526	7.334	7.5385	8.3767
ENSG00000112149	CD83	13.3475	13.2777	11.9869	12.5831	10.6399	12.2434	13.0904	13.1351	11.182	12.0693	9.1116	9.9596	9.582	9.5522	10.3457	11.2292
ENSG00000164649	CDCA7L	11.5941	11.7653	11.9048	12.4	8.8992	11.8373	11.8996	11.3323	10.7457	9.861	11.0664	11.7147	11.7109	10.7374	7.9138	10.2388
ENSG00000129910	CDH15	0	0	0	1.1177	0	0	0	0	0	0	0	1.0566	0.9928	0	0	1.0496
ENSG00000124762	CDKN1A	9.0336	9.5053	8.733	10.2494	8.8891	10.1894	10.3733	10.3268	9.5304	10.5136	9.4173	9.8558	10.9551	10.4535	10.2256	11.1583
ENSG00000243649	CFB	0	0	0	0	0	0	0	0	0	0	0	0	0	0	0	0
ENSG00000244255	CFB	0	0	0	0	0	0	0	0	0	0	0	0	0	0	0	0
ENSG00000003402	CFLAR	13.2638	12.2012	10.8748	11.2107	9.534	10.7559	12.9605	11.8165	11.182	11.238	10.0633	10.8525	10.8747	9.9743	10.2659	11.0494
ENSG00000064886	CHI3L2	8.1075	7.7881	8.9193	8.5971	8.4436	9.0228	9.3588	8.6541	5.4718	5.9303	7.3264	6.9557	8.3131	7.4125	6.3839	6.7201
ENSG00000188037	CLCN1	0	0	0	0	0	1.1043	0.9709	1.0566	0	0	0	1.0566	0	0	0	0
ENSG00000132514	CLEC10A	0	1.1043	0	0	0	0	0	0	0	0	0	0	0	0	0	0
ENSG00000131143	COX4I1	12.1948	11.6182	12.0601	12.1834	12.5498	11.8761	12.4881	11.7065	11.8226	13.4063	12.2153	12.1959	12.3896	11.6264	12.1495	12.0345
ENSG00000117322	CR2	6.9375	7.8325	6.5125	8.2135	4.8575	7.803	8.1587	7.6026	3.9505	4.3176	4.2965	4.6915	5.1526	4.4886	3.0652	4.1739
ENSG00000118260	CREB1	10.1413	10.3524	9.8641	10.5532	7.9937	10.3206	10.4679	10.3146	9.5076	9.6896	9.848	10.4045	10.5319	9.8243	8.4459	9.9181
ENSG00000108255	CRYBA1	0	1.7225	0	1.1177	1.2203	0	1.541	1.0566	0.9709	0	2.2418	1.0566	1.9855	0	1.3045	1.0496
ENSG00000164400	CSF2	1.4383	0	0	0	0	0	0.9709	0	0	0	0	0	0	0	0	0
ENSG00000108342	CSF3	0	0	0	0	0	0	0	0	0	0	0	1.0566	0	0	0	0
ENSG00000101266	CSNK2A1	10.2446	10.494	9.8655	10.6289	9.093	10.6682	11.1633	11.0197	10.5123	11.2361	10.5569	11.0602	11.4313	10.7694	10.0189	11.3324
ENSG00000175183	CSRP2	2.8053	2.9837	2.7203	4.3896	3.841	4.9973	4.1859	4.1922	3.275	3.85	1.5211	3.9107	4.5065	4.0712	3.4033	4.673
ENSG00000077984	CST7	0	2.157	0	0	0	2.4803	0.9709	2.4114	0	0.84	0	1.0566	0	0	1.9819	2.0704
ENSG00000109861	CTSC	10.954	11.4702	10.5903	11.8069	10.1308	12.2061	11.8927	12.3226	12.0742	12.5092	12.3755	12.1129	13.0719	11.8374	11.643	12.3243
ENSG00000085733	CTTN	6.393	5.1981	4.4861	4.218	2.8074	3.7666	6.0016	4.6299	7.8663	9.1549	7.6616	8.7256	7.6757	6.9016	6.2365	7.6836

Ensembl Gene ID	Gene Symbol	LY10 24HR A a Log2 (TMM Counts+ 1)	LY10 24HR A b Log2 (TMM Counts+ 1)	LY10 24HR C a Log2 (TMM Counts+ 1)	LY10 24HR C b Log2 (TMM Counts+ 1)	LY10 24HR I a Log2 (TMM Counts+ 1)	LY10 24HR I b Log2 (TMM Counts+ 1)	LY10 24HR V a Log2 (TMM Counts+ 1)	LY10 24HR V b Log2 (TMM Counts+ 1)	TMD8 24HR A a Log2 (TMM Counts+ 1)	TMD8 24HR A b Log2 (TMM Counts+ 1)	TMD8 24HR C a Log2 (TMM Counts+ 1)	TMD8 24HR C b Log2 (TMM Counts+ 1)	TMD8 24HR I a Log2 (TMM Counts+ 1)	TMD8 24HR I b Log2 (TMM Counts+ 1)	TMD8 24HR V a Log2 (TMM Counts+ 1)	TMD8 24HR V b Log2 (TMM Counts+ 1)
ENSG00000142544	CTU1	6.8757	6.3714	6.0067	6.3782	6.619	6.384	7.0435	6.4676	6.6757	8.2218	6.8546	6.3386	6.9248	6.3021	6.6503	6.9364
ENSG00000169245	CXCL10	13.0217	11.4232	7.632	7.8957	6.8025	7.6468	12.917	11.0159	8.5285	7.959	3.0807	2.4114	4.5065	3.7463	7.9658	7.7916
ENSG00000156234	CXCL13	3.6005	5.9554	0	3.5323	0	1.1043	0.9709	3.0976	3.6519	5.4205	0	1.6599	2.9837	2.4647	5.1843	5.4469
ENSG00000138755	CXCL9	7.4705	6.9165	2.9107	2.7803	2.1144	3.7666	6.935	6.9808	4.4094	3.85	0	0	1.5753	2.1342	2.4383	4.1739
ENSG00000008283	CYB561	6.2544	5.557	5.9208	5.3954	4.0866	5.2787	5.7936	5.8097	3.7581	4.7986	2.9126	3.0976	4.0704	2.7334	3.2987	4.673
ENSG00000166347	CYB5A	0	0	0	1.1177	0	0	0	0	8.5323	9.7074	8.2263	8.1551	8.2013	7.8255	8.5824	8.7061
ENSG00000134716	CYP2J2	0	0	0	0	0	0	0	0	2.541	5.0938	3.2311	4.1922	3.9837	4.1651	2.9317	3.4046
ENSG00000001630	CYP51A1	6.027	5.0557	6.1835	5.7503	1.2203	6.2815	7.5047	6.2998	6.2517	0	5.6294	6.2797	7.7371	6.2813	2.4383	7.2809
ENSG00000155368	DBI	11.6639	10.3216	10.9521	10.4504	11.853	10.8168	12.2995	11.3189	11.3206	12.2529	11.6507	10.8668	11.9662	11.0962	12.285	11.8369
ENSG00000105516	DBP	8.6187	8.4981	8.5445	9.0764	8.5905	8.8437	9.1269	8.3825	7.2513	8.384	7.9388	8.0591	8.3086	7.1163	6.7818	6.9857
ENSG00000165732	DDX21	11.7734	11.7763	9.9962	10.8284	8.968	11.4528	12.9447	12.7135	11.9602	11.8623	10.5747	11.2885	11.9268	11.4755	11.1139	13.0026
ENSG00000125485	DDX31	9.3761	9.8904	8.5835	9.8587	7.5818	9.4979	9.6402	9.7712	9.0295	9.6603	8.7231	9.6008	9.3506	8.9364	7.804	9.6384
ENSG00000213782	DDX47	5.9086	6.2236	5.5135	6.7369	0	6.2382	6.0851	6.2596	4.903	4.3736	5.0317	5.9165	6.1721	5.8445	2.9317	6.0902
ENSG00000174485	DENND4A	9.9745	10.1033	8.7796	9.8086	6.7227	9.5429	10.2976	9.8237	8.415	7.7892	8.1899	8.9053	9.1916	8.3142	6.4583	8.5671
ENSG00000062282	DGAT2	6.4786	6.4305	6.4371	6.3983	5.8153	6.3234	6.5351	6.1757	7.4944	7.7527	6.5152	7.5534	7.5746	6.9939	6.3452	7.4039
ENSG00000128185	DGCR6L	8.4941	7.3217	8.1639	7.7848	7.4992	7.5354	8.4265	7.6576	7.4473	8.6982	7.8829	7.2084	7.7033	6.9016	7.1467	7.5669
ENSG00000114956	DGUOK	10.2293	10.0447	9.8231	10.312	10.1854	10.2693	10.6087	10.2533	9.5803	11.113	9.7514	9.6579	10.1595	9.4086	9.9016	9.8514
ENSG00000102796	DHRS12	6.4923	6.0834	6.4678	6.6212	5.0144	6.384	5.8897	5.8642	5.7296	6.2475	5.9885	6.239	6.0268	5.6952	5.0303	5.5817
ENSG00000178028	DMAP1	8.7334	8.7271	9.2278	9.7149	7.0473	9.2651	8.4543	8.4641	9.061	9.4222	9.3529	10.8609	10.2157	9.8737	7.2689	8.9953
ENSG00000254986	DPP3	8.9209	8.6001	8.3889	8.8747	7.467	9.165	9.7241	9.3303	9.1713	9.7006	8.5182	9.5033	9.8815	9.1973	7.8736	9.719
ENSG00000117505	DR1	9.7747	10.0681	9.6163	10.4564	8.1941	10.2693	10.6936	10.4928	9.8631	10.1794	10.1248	10.1825	10.9114	9.872	9.1946	10.37
ENSG00000136048	DRAM1	10.6715	9.0771	9.3126	8.7893	8.1269	8.5581	11.1663	9.3638	7.6128	8.5017	6.2758	6.7966	6.913	6.4584	7.2689	7.9241
ENSG00000158050	DUSP2	10.8488	10.4012	7.6454	8.6101	7.9937	9.0512	11.4077	11.0523	9.8037	11.2793	7.0296	7.6955	7.474	7.2825	9.9625	10.2514
ENSG00000105246	EBI3	9.5006	6.8169	4.8074	4.6135	4.0866	2.9782	9.4621	6.6648	7.8116	8.9675	2.5008	3.4222	4.153	2.4647	7.6851	8.1192
ENSG00000117298	ECE1	11.5703	11.3752	10.9357	10.8993	8.7818	10.5759	11.5734	11.1309	10.8639	11.0537	10.2659	11.3187	11.4042	10.819	9.0577	11.4666
ENSG00000172638	EFEMP2	5.1039	3.6462	4.9001	4.218	3.272	3.8826	3.5248	2.903	3.6519	4.7148	4.6582	5.4303	4.5065	3.1538	2.7845	3.5435
ENSG00000090776	EFNB1	6.1363	5.557	5.9857	6.3782	4.3916	5.8369	6.4431	6.2182	5.4718	6.2018	4.7613	5.4303	6.2304	5.5975	4.2795	5.9971
ENSG00000130741	EIF2S3	12.1744	12.2525	11.855	12.5329	11.091	12.3104	12.412	12.2971	11.2692	11.8598	11.0055	11.7007	12.0782	11.4524	10.7205	11.9796
ENSG00000132507	EIF5A	12.4032	11.9985	11.1026	11.5248	11.9626	12.0224	13.3427	12.8793	11.9584	13.3754	11.0676	11.4442	11.7265	11.256	12.1944	12.7485
ENSG00000253626	EIF5AL1	6.5196	6.7098	5.6267	5.6859	6.6091	6.6703	7.2478	7.5702	6.5176	8.3213	5.0725	5.4303	5.7104	5.5635	7.0933	7.339
ENSG00000163435	ELF3	6.6483	5.8728	4.5447	5.3954	4.7892	5.3614	7.4267	6.5024	1.5509	3.9269	0.9486	2.0841	1.5753	2.1342	0.7991	3.669
ENSG00000126767	ELK1	8.3875	8.3583	8.0264	8.7465	6.6575	8.7197	9.2061	8.9691	8.2095	8.9583	8.1667	8.6693	8.7337	8.1288	7.169	8.7849
ENSG00000118985	ELL2	11.6977	11.545	10.1903	10.7191	8.6587	10.3475	12.1166	11.6668	9.2647	9.1449	8.2173	8.3348	8.9906	8.0814	8.7249	9.6988
ENSG00000134759	ELP2	10.3392	10.4435	9.5461	10.603	8.5137	10.2786	10.6131	10.3121	10.3923	10.8833	10.4319	10.9605	10.9912	10.3261	9.413	10.8118
ENSG00000142227	EMP3	7.1524	6.3714	7.7737	7.8814	7.8174	7.4993	7.9954	7.1658	7.5474	8.7173	8.3551	8.2706	9.3787	8.5799	9.1296	9.2233
ENSG00000174837	EMR1	1.4383	1.1043	0	0	0	1.7181	2.269	1.0566	0	2.3074	0	1.0566	0	0	0	1.0496
ENSG00000074800	ENO1	13.4552	12.9162	11.8996	12.2129	12.3236	12.9347	14.6917	14.0761	13.7698	14.528	12.6935	13.1166	13.6349	13.0956	13.8428	14.6281
ENSG00000154928	EPHB1	10.6254	9.977	10.3283	9.8403	8.4519	9.1534	10.3172	9.3182	9.0585	9.5673	7.9056	8.6107	8.6964	7.5318	7.7752	8.7674
ENSG00000133106	EPSTI1	7.6349	8.2368	7.7364	8.8233	6.6952	8.5537	7.1149	7.8657	6.6482	7.474	7.301	7.1978	8.6042	7.8955	6.8009	7.5505
ENSG00000164307	ERAP1	10.67	10.8406	10.3786	11.2257	8.0346	11.1229	11.2001	10.8712	10.1628	10.1456	10.2159	10.8576	11.1369	10.3882	8.6816	10.7318
ENSG00000112851	ERBB2IP	11.0054	11.1955	10.7368	11.5219	8.6372	11.1303	10.9891	10.8169	10.1358	10.11	10.9212	11.5023	11.5894	10.7806	8.8079	10.6557
ENSG00000012061	ERCC1	10.1843	10.1123	10.2063	10.6311	9.9067	10.4842	10.4463	10.0091	9.6232	10.764	9.9738	10.3132	10.3408	9.718	9.1541	9.617
ENSG00000162694	EXTL2	8.5246	8.8654	8.6685	9.3782	6.785	9.2047	8.3735	8.3494	7.6479	7.7684	8.4696	8.7655	9.1344	8.4461	6.6291	7.7278
ENSG00000158161	EYA3	8.9487	9.4278	9.0693	9.9385	8.2136	9.7776	9.6995	9.5562	7.2237	8.2256	7.3596	8.6226	8.6439	8.02	6.4461	8.0856
ENSG00000151327	FAM177A1	7.6098	7.9243	6.9088	7.992	7.0832	7.6135	8.0589	8.1333	7.3226	8.2705	7.301	7.4503	7.6332	7.0318	7.1764	7.4574
ENSG00000111913	FAM65B	12.2297	12.0527	11.776	11.8971	10.4217	11.7937	12.2677	11.7784	11.2987	11.3569	11.2664	12.074	12.4057	11.5021	10.1833	11.8933
ENSG00000230701	FBXW4P1	1.4383	0	2.4983	2.1763	0	2.7506	0	1.6599	0.9709	0	0	1.0566	2.3074	1.0909	0	1.0496
ENSG00000162897	FCAMR	0.8953	2.488	1.9222	2.1763	2.3219	2.1506	2.943	2.903	0	0	0	1.0566	0	0	0	2.0704
ENSG00000186431	FCAR	0	0	0	0	0	0	0	0	0	0	0	0	1.5753	0	0	0
ENSG00000119616	FCF1	9.1183	9.1433	8.6181	9.0607	7.5045	9.1329	9.6402	9.5013	9.1592	9.2346	8.9109	9.0738	9.6826	8.9027	8.2643	9.4157
ENSG00000132704	FCRL2	11.868	11.6319	12.1717	12.069	9.9752	11.3957	11.1525	10.3401	11.3379	10.9202	11.736	11.8916	12.632	11.3038	9.9931	10.8169
ENSG00000163518	FCRL4	4.7835	5.4845	2.4983	4.0233	1.2203	4.774	4.8435	5.4306	5.5632	6.2325	2.2418	5.0618	5.3045	5.6633	4.1052	6.805
ENSG00000168496	FEN1	10.7907	10.0665	9.4856	9.3025	9.4985	9.921	11.9079	11.4405	10.1845	10.8639	8.6345	8.8182	9.3894	8.8223	9.9432	10.8564
ENSG00000149781	FERMT3	9.68	9.3484	10.0093	10.3	9.0982	10.2119	10.0273	9.4797	9.1809	9.8708	9.9576	10.0329	10.2217	9.1861	7.8736	8.6031
ENSG00000149557	FEZ1	8.9661	7.8325	7.0674	6.6885	6.3152	6.7024	9.769	8.5373	8.8391	9.8023	6.9004	7.8454	7.9878	8.2884	8.599	9.8892

Ensembl Gene ID	Gene Symbol	LY10 24HR A a Log2 (TMM Counts+ 1)	LY10 24HR A b Log2 (TMM Counts+ 1)	LY10 24HR C a Log2 (TMM Counts+ 1)	LY10 24HR C b Log2 (TMM Counts+ 1)	LY10 24HR I a Log2 (TMM Counts+ 1)	LY10 24HR I b Log2 (TMM Counts+ 1)	LY10 24HR V a Log2 (TMM Counts+ 1)	LY10 24HR V b Log2 (TMM Counts+ 1)	TMD8 24HR A a Log2 (TMM Counts+ 1)	TMD8 24HR A b Log2 (TMM Counts+ 1)	TMD8 24HR C a Log2 (TMM Counts+ 1)	TMD8 24HR C b Log2 (TMM Counts+ 1)	TMD8 24HR I a Log2 (TMM Counts+ 1)	TMD8 24HR I b Log2 (TMM Counts+ 1)	TMD8 24HR V a Log2 (TMM Counts+ 1)	TMD8 24HR V b Log2 (TMM Counts+ 1)
ENSG00000183386	FHL3	7.4209	7.5158	6.5702	7.7538	6.1491	7.5084	8.0222	7.6185	5.1965	6.851	4.2965	5.7814	4.9369	5.1699	5.1546	6.3759
ENSG00000172500	FIBP	9.8081	9.6426	9.5425	9.8947	9.6317	9.8093	10.3301	9.8151	9.1809	10.5887	9.3112	9.6345	9.4998	8.979	9.1689	9.4578
ENSG00000119321	FKBP15	9.2253	9.1462	8.1592	9.0318	6.1218	9.0196	9.3651	9.3543	7.6199	8.2103	7.8425	9.1553	8.7766	8.1914	6.1791	8.5385
ENSG00000137312	FLOT1	8.0758	7.5248	8.2721	8.5391	6.811	8.2512	8.979	7.7618	8.881	9.3525	8.6037	9.0328	9.6278	8.8366	8.3252	9.7515
ENSG00000162769	FLVCR1	8.7305	8.911	8.0919	8.6902	7.0473	8.6349	9.5548	9.1223	7.8663	9.0189	7.5148	8.5244	8.4209	7.8609	7.4979	8.7244
ENSG00000129654	FOXJ1	0	0	0	1.1177	0	1.7181	0.9709	0	0	0	0	0	0	0	0	1.6462
ENSG00000151474	FRMD4A	9.1116	8.8936	9.1472	8.9925	7.9122	8.8757	9.4042	8.7941	9.3819	10.036	8.7295	9.347	9.4271	8.6666	7.9994	9.619
ENSG00000089280	FUS	12.6994	12.734	12.0082	12.6346	11.2423	12.593	13.2735	13.1569	12.3206	13.1028	12.0097	12.964	12.7087	12.388	11.3229	12.8061
ENSG00000196371	FUT4	7.0254	7.3112	5.6536	7.0108	4.4803	6.9364	7.474	7.6653	1.5509	2.0566	0	2.0841	2.7929	1.0909	0.7991	1.0496
ENSG00000143458	GABPB2	6.8111	7.9106	6.8976	7.8451	5.7452	7.6135	7.2658	7.5201	6.0141	6.6042	5.4867	6.7394	6.2491	5.9001	3.757	5.9481
ENSG00000099860	GADD45B	9.7619	9.2563	8.9248	9.731	8.6153	9.0941	10.1207	9.4621	8.8053	9.9291	8.8742	9.1689	9.3723	8.6424	8.7888	9.2813
ENSG00000183087	GAS6	5.9086	6.2236	5.7811	5.8417	4.8237	6.384	6.5201	6.0164	0	1.3674	0	1.0566	0	1.0909	0.7991	0
ENSG00000125965	GDF5	0	1.1043	0	1.7442	0	0	0	0	0	0	0	0	0.9928	0	0	0
ENSG00000101003	GINS1	8.9784	8.6465	7.4595	7.7848	7.2313	8.4055	10.0638	9.7942	8.521	9.0428	6.4552	6.8244	7.5969	7.3033	8.3482	9.4945
ENSG00000178445	GLDC	0	1.1043	0.9486	2.1763	0	2.1506	4.1859	2.0841	0.9709	0	0	0	0	1.0909	0.7991	2.3951
ENSG00000060558	GNA15	0.8953	1.1043	1.9222	0	0	0	0	0	4.4731	5.5954	4.4276	3.6871	5.1526	4.3363	4.8938	5.6747
ENSG00000242616	GNG10	4.4296	2.488	3.4906	1.7442	0	2.9782	4.9809	3.8032	5.306	3.4931	4.7104	4.4276	4.6264	4.1651	2.9317	4.9955
ENSG00000174021	GNG5	9.8701	9.3862	9.0693	9.7329	10.6732	9.7062	10.6384	10.2211	9.625	11.5685	9.6908	9.4374	9.9202	9.1027	10.822	10.1763
ENSG00000204590	GNL1	8.3001	8.2423	8.2546	8.3074	6.1491	8.1617	8.9899	8.5204	7.89	7.8742	7.5439	8.3822	8.3915	7.6821	6.2645	8.1627
ENSG00000143457	GOLPH3L	7.9162	8.4129	8.6718	9.1676	7.3481	8.8437	8.3859	8.3964	8.3354	8.6761	8.5687	8.9279	9.4168	8.5841	7.5724	8.6987
ENSG00000183484	GPR132	2.6182	3.6462	3.366	5.0312	2.1144	4.8334	2.7506	3.8032	0.9709	1.7527	0	2.4114	3.1538	2.9579	1.9819	3.8924
ENSG00000169508	GPR183	10.783	8.1162	10.0792	8.5572	8.8312	9.0574	10.4601	8.4056	9.8406	10.0914	9.8125	9.2428	10.529	9.411	9.6991	10.3107
ENSG00000139572	GPR84	0	0	0	0	0	0	0	0	0	0	0	1.0566	0.9928	2.1342	0	1.6462
ENSG00000233276	GPX1	9.9309	9.1521	9.9909	9.7547	10.6691	9.8493	10.6886	9.8203	10.2544	11.5606	11.1254	10.353	11.0735	10.0221	10.8424	10.3746
ENSG00000023171	GRAMD1B	9.1685	8.6214	8.1355	8.1553	6.1079	8.1501	9.2292	8.7184	7.8603	8.47	6.0507	8.3539	8.0854	8.1858	6.3054	9.2361
ENSG00000109519	GRPEL1	9.1771	9.2094	8.0213	8.7969	8.1096	9.3256	10.28	10.1638	8.3267	8.9652	7.5074	7.9436	8.322	7.9092	8.5824	9.2488
ENSG00000125484	GTF3C4	9.215	9.7565	8.58	9.976	7.8758	9.72	10.0247	10.1773	9.0241	9.342	8.7006	9.5097	9.6947	9.0544	8.1641	9.9726
ENSG00000107937	GTPBP4	10.4324	10.4231	8.9276	9.7703	8.5111	10.2585	11.4183	11.2412	10.4321	10.6238	9.6293	10.1389	10.635	10.1466	9.8667	11.3004
ENSG00000136732	GYPC	9.8754	9.628	9.4126	9.9676	9.0501	9.8582	10.5167	9.851	8.9217	9.815	8.5828	8.8456	9.1268	8.2779	9.1708	9.5571
ENSG00000084754	HADHA	11.427	11.2463	11.4827	11.7739	10.1655	11.6333	11.8331	11.4179	11.0995	11.8068	11.1965	11.6757	11.9891	11.387	10.6321	12.0621
ENSG00000101336	HCK	11.3599	10.8577	11.2912	10.9532	10.1144	10.4047	11.4576	10.4241	11.7554	12.9127	12.4133	12.9387	12.9733	12.3616	10.8897	11.9472
ENSG00000126264	HCST	7.7598	6.8315	7.9028	7.8886	8.7531	7.4049	8.1826	7.2191	4.0383	5.1268	4.99	5.1953	5.6264	4.1651	4.6188	3.9928
ENSG00000119285	HEATR1	10.0844	10.6186	9.1185	10.0076	5.7981	10.3789	11.1219	11.1974	9.1222	8.0733	8.1853	9.1498	9.851	9.3399	7.0458	10.3143
ENSG00000198339	HIST1H4I	3.8777	4.6548	5.4535	5.5481	3.841	5.4396	4.26	3.9107	5.2705	6.5073	6.6285	6.7394	6.7371	5.9796	5.756	6.1564
ENSG00000197914	HIST1H4K	2.6182	2.157	3.366	4.4679	3.1699	3.3462	1.541	2.903	2.281	4.6253	4.2965	5.7814	5.7897	4.4886	2.2265	3.8924
ENSG00000234745	HLA-B	8.0198	7.9447	8.2369	8.6274	6.351	8.1559	8.1245	7.5702	9.5133	10.4717	9.8982	9.8592	10.4869	9.5347	9.4889	9.6307
ENSG00000204642	HLA-F	5.5084	5.0045	5.4535	5.0312	5.044	4.89	5.4594	3.5607	4.1977	5.8924	4.0755	5.0144	5.2304	4.627	3.757	4.1739
ENSG00000204632	HLA-G	2.144	1.1043	0.9486	1.1177	0	1.7181	0.9709	1.0566	1.5509	0	2.2418	2.0841	0	0	0.7991	0
ENSG00000108924	HLF	5.4529	6.0336	3.366	4.7458	3.3688	5.0976	5.4279	5.1081	1.9598	0	0	1.6599	2.3074	1.0909	0	0
ENSG00000182952	HMGN4	9.9916	9.8531	9.7329	10.1471	7.4506	10.0867	10.6245	9.8272	9.1543	9.1529	9.2284	9.556	9.7668	8.9822	8.0762	9.3884
ENSG00000150540	HNMT	0.8953	1.1043	0	1.1177	0	1.7181	1.541	0	0	0	0	0	0	0	0	0
ENSG00000122592	HOXA7	1.4383	3.1795	1.516	1.1177	0	1.7181	2.269	2.4114	0	0	0	1.0566	0	0	0	1.6462
ENSG00000176387	HSD11B2	0.8953	0	0	1.1177	0	1.7181	3.5248	1.6599	4.7565	5.8537	0	0	2.9837	2.9579	4.0418	4.8958
ENSG00000096384	HSP90AB1	14.0839	14.1888	12.6022	13.755	12.6436	14.2311	15.3579	15.1115	14.0515	14.6421	12.888	13.5686	14.0667	13.7221	13.4939	15.0824
ENSG00000166598	HSP90B1	14.5349	14.5381	15.1618	15.5714	13.5863	15.2305	15.0669	14.8468	12.4996	12.6797	13.0138	13.208	13.7939	12.9148	12.0859	13.4119
ENSG00000204388	HSPA1B	0	0	0	0	0	0	0	0	0	0	0	0	0	0	0	0
ENSG00000164070	HSPA4L	5.6129	5.785	3.079	5.4738	4.5229	5.3208	7.4025	6.6648	6.7421	6.851	4.4886	5.7236	6.1522	6.0052	6.4338	8.1574
ENSG00000090339	ICAM1	7.9566	8.2641	6.9088	8.1374	2.5008	8.0097	8.5383	8.5076	9.0663	8.5542	7.3348	8.5538	8.7733	8.3792	7.3362	9.6518
ENSG00000105371	ICAM4	0	0	0	0	0	0	0	0	0	0	0	0	0	0	0	0
ENSG00000115738	ID2	10.7074	9.3175	8.8218	9.1823	7.8301	8.6349	10.8429	9.3543	9.6494	10.2047	7.5935	7.3002	7.8951	7.514	9.7883	10.2362
ENSG00000137331	IER3	1.4383	1.1043	1.516	1.1177	0	1.1043	0.9709	0	1.5509	2.5211	0	1.0566	0.9928	0	0.7991	3.251
ENSG00000068079	IFI35	4.9919	6.1552	6.3243	6.1821	3.5435	6.3438	4.3298	6.132	7.5622	8.6193	8.2836	8.4417	9.0563	8.0694	7.0618	7.905
ENSG00000137959	IFI44L	6.4077	8.7114	4.9001	9.0479	0	8.6266	5.1843	8.6267	4.903	3.3882	4.0755	4.862	6.948	6.4584	3.9069	6.0208
ENSG00000115267	IFIH1	8.7847	9.2009	7.373	9.2054	5.7808	8.8437	8.7548	9.0089	8.318	8.4043	7.9443	8.6226	9.0401	8.4966	6.9715	8.853
ENSG00000185745	IFIT1	5.1739	7.0973	5.5714	8.1374	3.7719	7.8682	4.3298	7.1217	5.5333	5.4205	5.1502	5.3561	8.4899	7.9159	5.9914	7.5989

Ensembl Gene ID	Gene Symbol	LY10 24HR A a Log2 (TMM Counts+ 1)	LY10 24HR A b Log2 (TMM Counts+ 1)	LY10 24HR C a Log2 (TMM Counts+ 1)	LY10 24HR C b Log2 (TMM Counts+ 1)	LY10 24HR I a Log2 (TMM Counts+ 1)	LY10 24HR I b Log2 (TMM Counts+ 1)	LY10 24HR V a Log2 (TMM Counts+ 1)	LY10 24HR V b Log2 (TMM Counts+ 1)	TMD8 24HR A a Log2 (TMM Counts+ 1)	TMD8 24HR A b Log2 (TMM Counts+ 1)	TMD8 24HR C a Log2 (TMM Counts+ 1)	TMD8 24HR C b Log2 (TMM Counts+ 1)	TMD8 24HR I a Log2 (TMM Counts+ 1)	TMD8 24HR I b Log2 (TMM Counts+ 1)	TMD8 24HR V a Log2 (TMM Counts+ 1)	TMD8 24HR V b Log2 (TMM Counts+ 1)
ENSG00000119917	IFIT3	5.9885	8.5344	5.8758	9.5598	5.554	9.0911	6.4271	8.486	4.9491	6.0383	4.7104	5.4303	7.6617	7.344	5.2133	7.0567
ENSG00000027697	IFNGR1	8.5773	7.7805	8.3107	7.9719	6.2909	7.8823	8.716	7.8724	8.8238	8.7253	9.0469	8.889	9.5633	8.6865	7.8781	9.0398
ENSG00000096872	IFT74	8.0666	7.6707	8.0418	8.4205	6.2662	7.8611	7.774	7.32	5.6488	5.7087	6.0708	5.9919	6.6966	5.6308	5.2415	5.7336
ENSG00000136634	IL10	10.361	10.4847	5.1851	6.009	3.6241	6.7182	10.5829	10.1168	6.0566	6.5198	0.9486	1.6599	0.9928	3.6194	6.2919	7.5174
ENSG00000168811	IL12A	7.7426	7.2471	6.9528	6.7526	5.4912	7.0778	8.3651	7.5201	5.4403	5.8537	4.2965	5.3176	4.9828	4.6915	5.6328	6.6757
ENSG00000113302	IL12B	6.6234	5.0045	0	1.7442	0	1.1043	6.8775	5.0148	4.4094	3.1522	0	3.0976	1.9855	1.7049	4.3847	5.5491
ENSG00000125538	IL1B	0.8953	0	0	0	0	1.1043	1.541	1.0566	0	0	0	0	0	0	0	0
ENSG00000147168	IL2RG	10.081	10.0835	9.7502	10.2256	9.5507	10.0622	9.9762	9.7801	9.8572	11.2328	10.6653	10.9384	11.1249	10.4686	9.7444	10.0252
ENSG00000104951	IL4I1	4.1102	4.1922	2.2388	2.1763	1.8758	2.4803	6.0851	5.394	4.5342	5.5232	2.7225	4.1922	3.8904	3.9709	6.7425	7.3672
ENSG00000136244	IL6	4.0365	5.0045	2.4983	4.306	1.585	3.1731	3.846	4.5656	0.9709	3.7687	3.4931	1.0566	0	2.1342	3.8339	3.7856
ENSG00000134352	IL6ST	10.4171	10.5984	10.14	11.0053	8.4298	10.4842	10.2722	10.2761	5.1965	5.6188	5.5741	5.7236	5.9131	5.2578	4.2795	6.1346
ENSG00000169429	IL8	1.8359	1.1043	4.3611	2.1763	0	0	2.269	1.0566	0	2.0566	0	0	0	0	0	0
ENSG00000166333	ILK	5.9491	5.9009	6.1077	6.6885	0.7398	6.1479	6.4744	5.2784	4.856	4	5.8081	6.0866	6.1117	5.4189	1.6826	5.4469
ENSG00000139269	INHBE	3.121	3.9982	4.5447	4.8669	4.3448	4.9449	4.8909	4.3533	0.9709	1.7527	1.5211	3.5607	1.9855	2.7334	0	3.669
ENSG00000186480	INSIG1	12.4828	11.7417	12.3157	12.1709	11.0085	11.9702	13.3678	12.076	11.5879	11.9175	10.9697	11.2798	12.1066	11.3115	11.1052	12.0902
ENSG00000125347	IRF1	9.9245	9.8621	9.8926	10.381	8.933	10.2866	9.7403	9.6308	6.7931	7.3883	6.7708	8.0414	7.6332	7.0066	5.3498	6.749
ENSG00000137265	IRF4	13.9837	13.8347	12.1917	12.9906	10.727	12.8452	14.0993	13.7985	13.8348	14.3573	11.6198	12.3206	12.5244	12.0156	12.7695	14.2472
ENSG00000066583	ISOC1	8.5009	8.3223	7.8572	8.3804	7.0106	8.1732	9.3588	9.0884	7.8178	8.5359	7.4775	7.6728	8.0537	7.5921	7.6954	8.4178
ENSG00000161638	ITGA5	2.8053	2.488	0	1.1177	0	1.1043	2.5311	1.6599	0	1.7527	0	1.0566	2.3074	2.9579	0	2.0704
ENSG00000140678	ITGAX	10.0097	9.9369	8.8038	9.406	6.6286	9.2187	9.3075	9.1083	7.8054	7.4804	6.2758	7.1327	6.3747	5.7568	5.7162	7.492
ENSG00000198399	ITSN2	10.7082	11.1531	10.4418	11.4751	8.6706	11.2674	10.9645	10.8678	11.0668	11.019	11.1157	12.4689	12.3907	11.8795	9.0873	11.3695
ENSG00000116679	IVNS1ABP	12.3721	12.5121	11.66	12.754	10.1788	12.507	12.6977	12.1868	12.5495	12.9078	12.0884	12.9126	13.1051	12.6189	11.2923	12.906
ENSG00000161999	JMJD8	7.6595	7.0851	7.1263	7.706	4.6041	7.8178	8.327	7.8724	7.1374	7.1684	7.167	7.7105	7.757	7.0066	5.0303	7.4834
ENSG00000177606	JUN	9.4661	8.2313	6.8749	8.1553	5.1285	7.5619	9.2922	8.1877	7.7548	7.6879	6.5444	6.5022	7.2583	6.4951	6.9715	8.4445
ENSG00000171223	JUNB	9.0981	8.0859	7.7427	8.1906	7.225	7.8611	9.6505	8.3204	7.1275	8.486	7.0088	6.7248	7.0591	5.9269	7.005	7.492
ENSG00000104783	KCNN4	9.7845	8.7074	9.6008	8.9348	8.6492	8.8544	10.2107	9.0566	8.1323	9.7357	8.5255	8.6845	8.77	8.2025	8.2984	9.486
ENSG00000137261	KIAA0319	0.8953	1.1043	0.9486	3.3771	1.585	2.9782	0	3.0976	0.9709	0	0	2.0841	2.5705	1.7049	0	0
ENSG00000162849	KIF26B	9.2233	9.2344	8.6078	9.0638	6.5993	8.3509	8.0692	8.1877	8.7122	9.1982	6.0708	6.754	6.8402	6.5483	7.0377	8.3767
ENSG00000163884	KLF15	0	0	0	0	0	0	0	0	0	0	0	0	0	0	0	0
ENSG00000115919	KYNU	9.0846	8.4178	9.0339	8.8891	8.2233	8.8897	9.2855	8.7799	8.9697	9.3101	9.4154	9.0268	9.8525	9.0046	8.5114	9.3203
ENSG00000103642	LACTB	8.8611	8.6087	7.4672	8.6359	6.5385	8.1267	9.261	8.5331	7.9132	8.3908	7.8541	7.8855	8.4042	7.6662	7.6163	8.3487
ENSG00000196976	LAGE3	7.7992	7.2795	7.373	7.3695	8.5955	7.6955	8.3399	7.5201	6.9699	8.8759	7.6347	7.3101	7.211	6.649	7.6325	6.9364
ENSG00000078081	LAMP3	0	0	0	1.7442	0	1.1043	0	0	0	0	0	1.0566	0	0	0	0
ENSG00000086730	LAT2	11.1629	10.0415	10.4911	9.9488	9.5546	9.7141	11.7515	10.4928	7.6549	8.8613	7.9167	8.4327	9.3745	9.0788	8.39	9.4622
ENSG00000100097	LGALS1	7.2771	6.6938	7.7364	8.2306	8.6299	8.2458	8.0116	7.3585	9.9331	10.76	10.5106	9.6579	11.1223	9.8858	11.2099	10.3216
ENSG00000105370	LIM2	0	0	0	0	0	0	0	0	0	0	0	0	0	0	0	0
ENSG00000106683	LIMK1	10.0798	10.1317	10.851	11.2043	9.2809	11.0514	10.5446	10.1787	10.0649	10.5828	10.7967	11.4915	11.7959	11.1787	8.843	10.7929
ENSG00000144182	LIPT1	7.1348	7.4316	6.931	7.6564	6.1355	7.3349	7.6001	7.0174	6.2698	7.1037	6.2583	6.71	6.6546	6.2602	5.3234	6.2802
ENSG00000189067	LITAF	10.6798	10.6281	10.5134	10.9309	9.5196	10.4217	11.1219	10.4787	10.2499	11.1364	10.7333	11.0711	11.1651	10.4063	9.5958	10.4269
ENSG00000160789	LMNA	4.6421	4.2802	4.4249	5.0312	2.1144	4.6479	4.4607	4.3533	6.7027	7.2752	7.485	7.7326	7.4979	6.5827	5.851	7.0567
ENSG00000135363	LMO2	1.8359	2.757	0	2.7803	1.2203	2.7506	3.4005	3.0976	8.7547	10.6748	6.9447	8.1496	8.0046	8.4876	9.3862	10.6404
ENSG00000111684	LPCAT3	8.0666	8.1806	7.7737	8.4547	5.2847	8.1617	8.7127	8.4239	8.0349	8.2668	8.4155	8.7511	8.898	8.4921	6.7622	8.8295
ENSG00000171236	LRG1	5.757	5.1048	5.4535	6.3782	5.3334	5.4012	6.3277	5.4663	2.281	3.1522	4.153	3.8032	3.4436	2.4647	3.757	4.3349
ENSG00000184434	LRRC19	1.4383	2.157	0	2.511	1.585	0	0	1.6599	1.9598	0	2.7225	0	0	1.0909	0	0
ENSG00000137507	LRRC32	7.3394	5.4469	2.4983	4.3896	0	1.1043	6.935	4.0108	2.281	0	0	0	0	1.0909	2.9317	2.3951
ENSG00000148356	LRSAM1	7.9264	7.9581	8.2721	8.7661	6.9418	8.5885	8.2701	7.9118	8.152	9.0557	8.6277	9.3987	9.1744	8.6944	6.3839	8.5549
ENSG00000226979	LTA	3.7908	3.7729	2.2388	1.1177	0	2.7506	4.5807	4.0108	3.7581	4	3.4931	4.0108	3.4436	3.3262	1.3045	4.8958
ENSG00000227507	LTB	5.5877	5.0045	5.6536	6.009	3.0583	4.5091	4.0259	3.0976	7.2421	8.026	9.4446	9.4903	9.2348	8.3645	5.3234	5.7623
ENSG00000213903	LTB4R	7.3007	7.6707	7.2283	8.3074	5.26	7.7348	7.4427	7.2191	8.1762	8.0563	8.8305	10.0043	9.0293	9.0109	4.8575	8.1893
ENSG00000254087	LYN	11.7392	11.7913	11.2444	11.949	9.698	11.4552	11.7346	11.5265	12.885	13.3317	12.6954	13.2494	13.2899	12.6297	11.7931	13.04
ENSG00000011009	LYPLA2	9.3705	8.7271	8.9165	9.0351	8.6202	9.1678	9.8126	9.1582	8.0189	9.0748	8.4234	8.5579	8.9305	8.1802	7.8276	8.6071
ENSG00000140280	LYSMD2	7.1524	6.6282	5.4226	5.8417	4.3448	5.3208	7.1733	6.7396	6.6482	7.3043	5.6562	5.5348	5.7637	5.2998	6.3581	6.5336
ENSG00000104774	MAN2B1	10.7668	10.5604	10.3377	10.6182	6.5176	10.5252	10.9898	10.4399	11.1377	10.7692	11.0263	12.1185	11.9479	11.5535	8.0484	11.3431
ENSG00000107968	MAP3K8	10.219	10.0712	7.8514	8.9825	6.5281	8.5927	9.9762	9.5788	8.6408	8.8416	6.9447	7.9562	8.0537	7.4596	7.4193	8.5343
ENSG00000069956	MAPK6	9.3134	9.3044	8.0919	9.3448	6.934	9.2944	10.4688	10.1638	8.7707	8.5111	7.9112	8.2452	9.1114	8.219	8.0801	9.6821

Ensembl Gene ID	Gene Symbol	LY10 24HR A a Log2 (TMM Counts+ 1)	LY10 24HR A b Log2 (TMM Counts+ 1)	LY10 24HR C a Log2 (TMM Counts+ 1)	LY10 24HR C b Log2 (TMM Counts+ 1)	LY10 24HR I a Log2 (TMM Counts+ 1)	LY10 24HR I b Log2 (TMM Counts+ 1)	LY10 24HR V a Log2 (TMM Counts+ 1)	LY10 24HR V b Log2 (TMM Counts+ 1)	TMD8 24HR A a Log2 (TMM Counts+ 1)	TMD8 24HR A b Log2 (TMM Counts+ 1)	TMD8 24HR C a Log2 (TMM Counts+ 1)	TMD8 24HR C b Log2 (TMM Counts+ 1)	TMD8 24HR I a Log2 (TMM Counts+ 1)	TMD8 24HR I b Log2 (TMM Counts+ 1)	TMD8 24HR V a Log2 (TMM Counts+ 1)	TMD8 24HR V b Log2 (TMM Counts+ 1)
ENSG00000155130	MARCKS	11.9055	11.7178	11.1232	11.5594	10.3299	11.1005	11.4092	11.039	11.4757	12.4891	11.7125	11.7445	11.9077	11.0401	10.9425	11.2099
ENSG00000120539	MASTL	8.7247	8.7923	7.9525	8.4786	6.9181	8.4104	9.5289	9.3351	8.181	8.4571	6.7584	7.5451	7.8648	7.441	7.86	9.1736
ENSG00000131844	MCCC2	10.9104	11.2948	9.9494	11.1314	9.6558	11.3895	11.6831	11.7251	11.2453	11.955	10.7372	11.3315	11.7083	11.1409	10.8857	11.9845
ENSG00000166508	MCM7	12.647	12.0499	11.4154	11.367	10.5682	11.3329	13.1044	12.573	11.2401	11.9312	9.9481	10.6009	10.482	10.0516	10.2693	11.5849
ENSG00000014641	MDH1	12.2113	11.3889	11.5492	11.5916	11.2459	11.7674	12.7279	12.029	11.3135	11.9558	11.5782	11.1866	11.8505	11.0393	11.5968	11.9925
ENSG00000213999	MEF2B	5.9291	6.5416	6.5416	7.1485	3.7719	6.9229	6.5498	5.8372	6.9588	7.9077	7.966	8.5496	8.4498	7.9624	6.0725	7.5423
ENSG00000146834	MEPCE	10.3944	10.3739	9.7455	10.5236	9.1346	10.3639	10.5963	10.6915	9.7417	10.5513	9.1828	9.6267	9.7668	9.3524	8.5317	9.7692
ENSG00000165175	MID1IP1	8.8717	8.0675	9.4686	9.0253	8.8728	9.0036	9.7306	8.8826	8.2828	8.4538	9.3488	8.972	9.5876	8.4739	7.5212	8.0856
ENSG00000234883	MIR155HG	13.0276	11.4555	9.0568	9.1348	8.588	8.7197	12.5134	10.9661	10.9508	11.5348	7.86	7.7472	8.478	7.7442	10.7833	10.8556
ENSG00000078403	MLLT10	9.3441	9.7372	8.8247	9.9523	7.1113	9.8529	9.8829	9.8047	8.4352	8.6423	8.4116	9.3612	9.2882	8.7969	7.0377	8.9556
ENSG00000139428	MMAB	8.6375	7.7577	8.2414	7.8814	8.4979	8.376	9.3734	8.4685	7.9418	9.1368	7.8483	7.8788	8.7437	8.0874	8.1189	8.4707
ENSG00000123342	MMP19	2.144	0	0	0	0	0	2.269	1.6599	0	0	0	0	0.9928	0	0	0
ENSG00000137673	MMP7	7.9616	3.6462	5.2917	4.218	4.2472	4.0909	8.2476	4.6915	0	0	0	0	0	0	0	1.0496
ENSG00000118113	MMP8	0	0	0	1.1177	0	0	0	0	0	0	0	0	0	0	0	0
ENSG00000100985	MMP9	0	0	0	0	0	1.1043	0	0	0.9709	0.84	1.5211	1.0566	0	0	0	1.0496
ENSG00000118242	MREG	6.7086	6.3714	5.7565	6.9298	3.4594	7.1839	7.5931	7.0412	5.1583	4.839	4.9026	5.2373	6.1522	4.8694	4.1052	5.2987
ENSG00000185414	MRPL30	1.4383	1.7225	1.516	1.1177	0	1.1043	0.9709	0	0.9709	0	0	1.0566	0.9928	1.7049	0	1.6462
ENSG00000130312	MRPL34	8.8451	7.9581	8.2979	8.264	9.0465	8.2239	9.6368	8.4374	9.1616	10.6166	9.2103	8.7108	9.2061	8.208	9.6694	9.4202
ENSG00000048544	MRPS10	9.6709	9.6026	9.5869	10.045	9.114	10.1004	10.2932	10.0181	9.5583	10.1456	10.1582	10.2196	10.7445	9.7562	9.0715	10.037
ENSG00000125445	MRPS7	9.7157	9.1521	8.4478	9.1348	8.5372	9.4204	10.599	10.1056	9.4922	10.2428	9.1547	9.2834	10.0197	9.2965	9.7345	10.2702
ENSG00000053372	MRTO4	9.9194	9.4915	7.8572	8.7267	8.3318	9.4443	10.9966	10.6051	9.8161	10.5769	8.614	8.915	9.5614	8.8683	9.6243	10.5994
ENSG00000143033	MTF2	9.692	9.7775	9.4086	10.126	8.4574	9.7908	10.3215	9.9862	9.2488	9.5553	9.4349	9.5867	9.9779	9.3145	8.4428	9.7497
ENSG00000110921	MVK	8.5043	7.7188	8.3107	8.0249	8.3613	8.7469	9.5676	8.5664	8.6478	9.3937	8.3917	9.1553	9.7404	9.3623	8.4997	9.7136
ENSG00000157601	MX1	10.1303	11.1791	9.7282	11.7167	8.5216	11.654	10.213	11.5138	9.6598	10.2559	9.5791	10.1403	11.7725	11.1066	9.575	11.1332
ENSG00000183486	MX2	9.904	10.3987	9.9856	10.7607	9.208	10.8277	10.5204	10.5078	10.5133	11.2266	10.889	11.7138	11.7967	11.0585	9.4453	10.8631
ENSG00000136997	MYC	11.6869	11.6552	10.1578	10.7341	9.1982	10.4831	11.9864	11.5901	11.0932	11.7892	8.9739	9.8609	9.3809	9.4038	10.2668	11.1063
ENSG00000137474	MYO7A	9.1249	8.5872	6.5125	7.6896	4.6433	6.7182	9.2223	7.2704	5.4403	6.4434	0	3.5607	0	2.1342	2.4383	3.9928
ENSG00000103174	NAGPA	7.3546	7.3423	7.0061	8.0885	6.7764	7.6386	7.9291	7.5619	7.3484	8.545	7.5795	7.9872	7.8339	7.344	6.4822	7.583
ENSG00000105835	NAMPT	11.1901	11.0589	9.599	10.6586	8.6226	10.6672	12.1126	11.6911	10.4351	10.6005	9.8726	10.0477	10.7935	10.0546	10.2693	11.5084
ENSG00000187109	NAP1L1	15.0878	14.7149	14.7962	14.9602	13.8216	14.4222	14.9257	14.2959	13.3323	14.0469	13.1372	13.1533	13.5708	12.766	12.8526	13.4969
ENSG00000105402	NAPA	10.7089	10.4637	10.8968	11.3503	10.6797	11.1177	11.2508	10.9266	10.0505	11.421	10.1746	10.7016	10.7577	10.1737	9.8181	10.5744
ENSG00000116701	NCF2	11.7294	10.9935	10.4243	10.7479	8.8354	10.0607	11.4438	10.2749	9.1689	9.1267	8.0598	8.1876	8.886	7.8468	7.8087	8.611
ENSG00000115053	NCL	13.2563	13.3137	11.9422	12.7048	11.1181	13.2049	14.4836	14.2868	13.3387	13.5302	12.2972	12.9932	13.4006	13.0603	12.5228	14.4126
ENSG00000189430	NCR1	3.4944	4.5166	4.7586	4.9782	3.4594	5.5141	4.6375	4.1922	0	1.3674	0	0	0	0	0	0
ENSG00000072864	NDE1	8.8261	9.1896	8.5694	9.4085	6.9181	9.1211	9.6811	9.2404	8.0664	8.0304	7.6074	8.5371	8.4538	8.0262	6.4583	8.4312
ENSG00000123545	NDUFAF4	7.7936	7.3626	6.7178	7.2184	7.6882	7.5965	8.6727	8.3964	7.3313	8.1555	5.9234	6.6646	7.4416	6.7281	7.9869	8.1627
ENSG00000157191	NECAP2	9.4344	9.3149	9.2368	9.7527	8.7509	9.4395	9.6861	9.3448	10.2384	10.8392	9.823	10.4285	10.7015	10.1523	9.2392	9.9772
ENSG00000101096	NFATC2	8.538	9.027	8.145	9.3395	6.7498	8.8932	8.8713	8.4947	7.3399	7.512	7.3095	8.4551	8.3873	7.7442	6.3452	8.1411
ENSG00000050344	NFE2L3	9.7901	9.4181	8.6147	9.2506	7.1726	9.0101	9.9061	9.3182	8.1906	8.4603	7.1856	7.9687	8.2676	7.7743	7.2481	8.0969
ENSG00000109320	NFKB1	11.6104	11.6171	10.1554	10.7617	7.3888	10.8039	11.6436	11.5254	10.5884	10.5895	9.5827	10.5363	10.6169	10.0158	8.9262	10.6567
ENSG00000077150	NFKB2	10.6578	10.3987	8.6418	9.5188	7.3422	9.1062	10.7027	9.9473	10.1383	10.6129	8.7199	9.9659	9.6599	9.1777	8.4879	9.9339
ENSG00000100906	NFKBIA	11.0108	10.5449	9.173	9.8292	8.3525	9.449	10.8697	10.2961	9.8192	10.8627	8.0648	8.1768	8.3658	7.8183	9.4933	9.9557
ENSG00000104825	NFKBIB	9.0383	8.6256	7.8338	8.4156	8.1809	8.4999	9.7611	8.9937	8.521	9.5293	8.0084	8.1275	8.5213	7.8326	8.39	8.8826
ENSG00000146232	NFKBIE	11.6249	11.3789	9.4591	10.6343	8.9526	10.3962	12.2386	11.4545	9.4291	10.1234	8.4506	8.7145	9.1471	8.5146	8.7983	9.393
ENSG00000131669	NINJ1	9.0313	8.8725	9.3645	9.6553	9.2947	9.5115	9.582	9.281	5.7554	7.0445	6.2583	6.2797	6.7237	6.0551	6.3452	6.7912
ENSG00000105374	NKG7	3.9588	4.2802	4.9873	4.6135	0	4.0909	4.8435	4.0108	0	0	0	3.0976	2.5705	2.1342	0	2.8856
ENSG00000239672	NME1	10.2878	9.4846	8.1259	8.107	10.947	9.3638	11.3624	10.7232	10.1518	12.0773	8.9977	8.7655	9.8087	9.4901	11.5592	11.1414
ENSG00000141101	NOB1	9.6058	9.4822	8.8453	9.411	8.252	9.3359	10.1622	9.7092	9.2556	9.8301	8.614	9.2479	9.3979	8.9693	8.5629	9.9085
ENSG00000173145	NOC3L	10.4634	10.3229	9.6617	10.0125	8.3167	10.0167	10.952	10.4024	10.1046	10.1285	9.9206	9.9087	10.475	9.7025	9.3179	10.4392
ENSG00000167207	NOD2	4.5398	3.7729	3.366	3.6713	1.2203	3.3462	5.4279	4.0108	0.9709	0	0	2.4114	1.9855	1.0909	0.7991	1.0496
ENSG00000166197	NOLC1	11.4368	11.4819	9.7626	10.5666	9.3024	11.1065	12.4984	12.3326	11.4806	11.7821	9.9164	10.8965	11.1325	10.828	10.5657	12.3622
ENSG00000142546	NOSIP	10.3181	9.6111	10.5362	10.4024	10.7196	10.1448	10.919	9.8134	10.1122	11.0462	10.7317	10.3423	10.7887	9.9547	9.9389	10.2413
ENSG00000139910	NOVA1	0	0	0	0	0	0	0	0	0	0	0	0	0	0	0	0
ENSG00000126368	NR1D1	6.6234	6.487	5.5714	6.6382	4.9841	6.4981	7.3186	6.6648	6.1959	6.6502	5.1502	6.2595	5.9598	5.4931	4.435	6.4124
ENSG00000174738	NR1D2	8.1944	8.9418	8.2054	9.2944	5.4256	8.0224	7.5714	7.477	7.6057	7.4018	8.3877	8.4639	8.211	6.803	4.8938	6.7054

Ensembl Gene ID	Gene Symbol	LY10 24HR A a Log2 (TMM Counts+ 1)	LY10 24HR A b Log2 (TMM Counts+ 1)	LY10 24HR C a Log2 (TMM Counts+ 1)	LY10 24HR C b Log2 (TMM Counts+ 1)	LY10 24HR I a Log2 (TMM Counts+ 1)	LY10 24HR I b Log2 (TMM Counts+ 1)	LY10 24HR V a Log2 (TMM Counts+ 1)	LY10 24HR V b Log2 (TMM Counts+ 1)	TMD8 24HR A a Log2 (TMM Counts+ 1)	TMD8 24HR A b Log2 (TMM Counts+ 1)	TMD8 24HR C a Log2 (TMM Counts+ 1)	TMD8 24HR C b Log2 (TMM Counts+ 1)	TMD8 24HR I a Log2 (TMM Counts+ 1)	TMD8 24HR I b Log2 (TMM Counts+ 1)	TMD8 24HR V a Log2 (TMM Counts+ 1)	TMD8 24HR V b Log2 (TMM Counts+ 1)
ENSG00000213281	NRAS	10.1478	10.3203	9.6265	10.4792	8.6893	10.5375	10.8414	10.6299	10.4471	10.7355	10.6495	10.8635	11.3979	10.5127	9.7714	11.0127
ENSG00000021645	NRXN3	0	1.1043	1.516	0	0	1.7181	0	0	0	0	0	0	0	0	0	0
ENSG00000143228	NUF2	8.9361	8.5209	8.7076	8.8419	7.467	8.8651	9.8989	9.2985	8.7089	8.8784	8.0648	7.7399	8.478	7.9558	8.4641	9.5933
ENSG00000111581	NUP107	10.7703	10.65	9.9643	10.4037	8.5603	10.4703	11.6171	11.2178	9.8068	9.9291	9.4427	10.4741	10.1818	9.6966	8.6261	10.2924
ENSG00000111331	OAS3	9.1813	10.51	9.0288	11.0053	8.1026	11.374	10.107	10.8943	7.6057	7.7368	7.384	8.7219	9.2253	8.9027	6.8842	8.5992
ENSG00000104904	OAZ1	12.0922	11.6182	12.2147	12.3639	12.7898	12.2973	12.9285	12.2422	13.3538	14.8443	13.5789	13.5537	13.913	13.172	13.9281	13.8549
ENSG00000180304	OAZ2	8.8822	9.1695	9.1161	9.6885	8.0455	9.4009	9.3482	9.15	8.8452	9.77	9.1805	9.8726	10.162	9.4323	8.2677	9.4066
ENSG00000128699	ORMDL1	10.0024	10.1711	9.9508	10.8533	8.8248	10.4373	10.102	9.8134	9.461	10.3053	10.3015	10.6198	10.6133	9.9952	8.7547	9.667
ENSG00000099985	OSM	2.144	2.488	2.4983	2.7803	1.2203	2.7506	4.26	2.903	0.9709	0	0	0	0	1.7049	2.2265	2.0704
ENSG00000154814	OXNAD1	8.3577	8.2802	7.9525	8.2585	7.0832	8.5044	9.3503	8.7035	7.4944	8.1067	7.0398	7.6262	8.2629	7.7668	7.4561	8.5135
ENSG00000122884	P4HA1	9.692	9.4938	9.3665	9.954	8.3347	9.8294	10.186	9.8323	8.344	8.486	8.4312	8.5579	9.2205	8.4921	8.4182	8.6651
ENSG00000170515	PA2G4	11.9424	11.4778	10.4733	10.8993	10.9184	11.3889	13.1254	12.6363	11.0707	12.0987	10.2011	10.4796	10.6946	10.4628	11.179	12.0231
ENSG00000158006	PAFAH2	6.755	6.7719	5.9857	7.3066	4.9222	6.9229	6.3447	6.432	5.9923	5.6868	4.9472	6.1755	6.7371	5.6952	4.3327	5.7623
ENSG00000130669	PAK4	7.4565	7.5605	7.7301	7.9308	6.7675	7.7348	8.1587	7.7832	7.1275	8.069	7.447	7.7472	7.948	6.9811	6.4941	7.7494
ENSG00000099864	PALM	0	0	0	0	0	0	0	0	0	0	0	0	0	1.7049	0	0
ENSG00000135473	PAN2	10.3015	10.4507	10.4214	11.2313	8.4102	10.6207	10.3226	10.003	8.978	9.3011	9.6973	10.9675	10.423	9.9711	7.3879	9.3155
ENSG00000185813	PCYT2	8.9311	7.847	8.7827	8.3181	7.6882	8.4629	10.0339	8.5414	9.5341	10.0554	9.4388	9.7992	10.2001	9.6159	8.7816	10.2438
ENSG00000156973	PDE6D	8.7247	8.1977	8.4932	8.3444	8.5346	8.3201	9.2107	8.3155	6.3572	7.7737	7.3264	6.8516	6.8402	6.5996	6.332	6.5664
ENSG00000104213	PDGFRL	0	0	0	0	0	0	0	0	0	0	0	0	0	0	0	0
ENSG00000100029	PES1	10.6933	10.3216	9.4667	9.8947	8.7774	10.234	11.5312	11.0589	10.5085	10.9125	9.5489	10.104	10.5671	10.1849	9.582	11.2432
ENSG00000162928	PEX13	7.832	7.8178	7.4672	8.2859	6.2282	8.315	8.5309	8.2351	7.0973	7.1998	7.204	7.1764	8.1114	7.3639	6.4338	7.7494
ENSG00000139289	PHLDA1	12.3024	10.8595	11.4609	10.2256	10.123	9.7414	12.6774	10.9513	8.6786	9.4903	6.8311	6.9304	8.0907	6.6163	8.7324	9.4838
ENSG00000083535	PIBF1	9.4073	8.7734	8.9468	9.1735	6.988	8.7546	9.654	9.1083	8.0611	7.6143	8.0343	8.2857	8.4939	7.6821	6.9021	7.9304
ENSG00000060642	PIGV	7.9665	8.2202	7.7238	8.5022	6.811	7.944	7.5422	7.4235	6.1377	7.1605	6.573	7.2601	7.2769	6.4584	5.4506	6.1777
ENSG00000171608	PIK3CD	10.9174	10.5692	10.5648	10.8162	9.0947	10.6713	11.1367	10.3084	11.17	11.3789	10.7676	11.8365	11.8864	11.3943	9.6013	11.7693
ENSG00000085514	PILRA	1.8359	3.3533	3.8104	4.7458	4.2972	3.7666	4.1859	4.1043	1.9598	4	3.2311	2.6781	3.1538	3.4803	2.7845	2.6622
ENSG00000121716	PILRB	10.4117	10.251	10.221	10.5166	8.7487	10.3714	10.2531	10.0639	9.2304	9.974	9.3895	10.7044	9.9905	9.8615	7.5269	9.363
ENSG00000137193	PIM1	11.9978	11.911	11.7737	12.4044	10.4564	11.6499	11.8418	11.4298	11.3465	12.1812	11.1463	11.7441	10.9807	10.4171	9.8286	10.7154
ENSG00000118495	PLAGL1	8.6124	8.9948	6.8045	7.7538	4.5229	6.7947	8.3483	7.9996	7.8543	8.1555	5.8081	7.0645	7.0266	6.5308	6.7226	8.3721
ENSG00000115956	PLEK	13.6174	13.1541	11.7613	12.1676	10.765	12.241	13.3535	12.9201	12.9254	12.9764	11.2153	11.4268	12.1992	11.6212	11.7787	12.7998
ENSG00000166851	PLK1	9.136	9.3886	9.2849	9.8587	8.8911	10.0684	10.7824	10.4644	10.2476	10.7995	8.851	9.373	9.5968	9.3721	10.0066	11.2949
ENSG00000173846	PLK3	8.9004	8.663	7.8915	8.5345	6.3859	8.2888	8.9176	9.3206	7.8603	8.6452	6.6819	7.5199	7.5368	7.128	7.0458	8.2959
ENSG00000164050	PLXNB1	7.5251	6.7412	8.2808	7.738	5.8819	7.2613	7.3696	6.239	6.1767	6.9184	5.9456	7.8318	6.9011	6.5656	4.1651	6.3759
ENSG00000141682	PMAIP1	10.2868	10.3448	9.7392	11.1427	8.2955	10.5308	11.1428	10.867	9.2	8.7253	9.3343	8.9595	9.9637	8.8683	8.7021	9.5051
ENSG00000109099	PMP22	3.4944	3.3533	2.7203	0	0	1.7181	3.5248	2.903	0	2.7049	1.926	1.0566	0	1.0909	1.3045	1.0496
ENSG00000064933	PMS1	9.0777	9.1521	8.733	9.0701	7.199	9.0323	9.8003	9.3965	8.9275	9.2664	8.9631	9.514	9.4558	8.9297	7.9226	9.4424
ENSG00000070501	POLB	7.2367	7.1451	7.3812	7.9446	6.8697	7.605	7.889	7.1439	7.627	8.218	8.1667	8.1551	8.286	7.7289	7.4681	7.6836
ENSG00000175482	POLD4	7.9416	7.6208	8.53	8.8083	6.0365	8.2621	7.3612	7.0174	7.4232	7.8838	8.7945	9.0476	9.0265	8.1171	6.371	6.9488
ENSG00000171453	POLR1C	9.8885	9.4846	8.2721	8.9104	7.9042	8.9611	10.0919	9.681	9.5583	10.34	8.3261	8.8422	9.2277	8.5884	9.2233	9.7426
ENSG00000181222	POLR2A	11.4602	12.3178	11.4609	12.6916	9.7369	12.5567	11.9545	12.2674	10.8868	11.163	10.8501	11.9937	12.0289	11.6289	9.274	11.7498
ENSG00000148606	POLR3A	8.5675	9.1076	8.2765	9.1228	6.619	9.1032	9.4815	9.4927	8.6338	9.0079	8.3957	9.6424	9.626	9.2808	7.2689	9.8924
ENSG00000058600	POLR3E	9.5176	9.6796	8.3064	9.5072	8.0708	9.5002	10.2223	9.963	9.346	9.9886	8.8096	9.9151	9.6756	9.3697	8.6099	9.8859
ENSG00000204531	POU5F1	5.757	5.4469	5.1473	5.8417	3.5435	5.2353	5.6907	5.3565	4.342	5.1906	4.4886	5.1522	5.1119	3.4803	4.4828	4.6112
ENSG00000127125	PPCS	8.9487	8.4843	8.9386	9.0511	8.0346	8.7315	8.6592	8.1553	8.8269	9.52	9.4543	9.4329	9.7553	8.8753	8.0919	8.7098
ENSG00000110841	PPFIBP1	6.3633	7.1333	6.8165	7.9786	4.9537	7.7348	6.8775	7.4144	3.5361	3.591	3.9928	5.466	5.5972	4.6915	3.1859	5.2184
ENSG00000158615	PPP1R15B	9.6664	10.1243	9.3399	10.5213	7.9477	10.4253	10.6384	10.6021	9.048	9.2737	9.1641	9.8874	10.2109	9.4742	8.3612	9.9464
ENSG00000173281	PPP1R3B	3.9588	4.9519	4.9001	6.4765	2.3219	5.6185	4.6921	4.4276	5.9704	6.6727	7.2131	7.6183	7.5969	6.9811	4.9973	5.845
ENSG00000011485	PPP5C	10.7338	10.7643	10.5736	11.2157	8.8707	11.2078	11.7366	11.3961	10.3136	10.7126	9.7929	10.6208	10.7553	10.35	9.291	11.1896
ENSG00000057657	PRDM1	10.9371	11.6655	11.2907	12.4159	9.9404	11.7464	10.9728	11.3079	10.5481	11.2036	11.343	11.7879	12.0438	11.2072	9.9432	10.8746
ENSG00000019485	PRDM11	5.1391	5.0557	4.7586	5.6859	4.2972	5.2353	4.6921	4.4983	4.4731	5.8339	4.9026	5.7814	4.9828	4.9241	3.591	5.5157
ENSG00000152784	PRDM8	1.4383	3.3533	0.9486	3.2048	0	2.4803	1.9523	2.4114	0	2.7049	0.9486	2.4114	1.5753	1.0909	0	2.0704
ENSG00000131791	PRKAB2	8.7819	9.5121	8.6418	9.8422	6.7227	9.4632	8.8683	9.097	7.9304	8.2742	8.1945	9.0911	9.1916	8.5454	6.6073	8.4445
ENSG00000126457	PRMT1	12.2519	11.4177	10.8611	11.0523	10.8219	11.4546	13.2489	12.3434	11.7606	12.5873	11.2005	11.5389	12.0626	11.5344	11.8184	12.6836
ENSG00000108671	PSMD11	10.32	10.3714	9.7564	10.4049	9.3458	10.5071	11.3359	11.0398	10.0881	11.0553	9.7884	10.3988	10.6554	10.0577	10.0433	11.0186
ENSG00000092010	PSME1	11.4919	11.0866	11.0604	11.3747	11.3749	11.4475	12.0801	11.4852	10.9592	12.1871	11.0198	11.0455	11.476	10.828	11.3734	11.5299

Ensembl Gene ID	Gene Symbol	LY10 24HR A a Log2 (TMM Counts+ 1)	LY10 24HR A b Log2 (TMM Counts+ 1)	LY10 24HR C a Log2 (TMM Counts+ 1)	LY10 24HR C b Log2 (TMM Counts+ 1)	LY10 24HR I a Log2 (TMM Counts+ 1)	LY10 24HR I b Log2 (TMM Counts+ 1)	LY10 24HR V a Log2 (TMM Counts+ 1)	LY10 24HR V b Log2 (TMM Counts+ 1)	TMD8 24HR A a Log2 (TMM Counts+ 1)	TMD8 24HR A b Log2 (TMM Counts+ 1)	TMD8 24HR C a Log2 (TMM Counts+ 1)	TMD8 24HR C b Log2 (TMM Counts+ 1)	TMD8 24HR I a Log2 (TMM Counts+ 1)	TMD8 24HR I b Log2 (TMM Counts+ 1)	TMD8 24HR V a Log2 (TMM Counts+ 1)	TMD8 24HR V b Log2 (TMM Counts+ 1)
ENSG00000171522	PTGER4	10.3267	9.7699	7.7174	8.9069	6.4534	8.0663	10.2315	9.7712	9.0026	9.3297	7.6944	7.6651	7.9422	7.2614	8.295	9.3792
ENSG00000148334	PTGES2	9.3288	8.6382	8.4516	8.6779	8.6706	8.9677	10.1342	9.3685	9.0295	10.1141	8.7824	9.3422	9.2348	8.7139	8.6315	9.5261
ENSG00000110958	PTGES3	12.7337	12.5875	11.8179	12.4799	11.9645	12.6747	13.6731	13.4229	11.6853	12.6867	11.3279	11.4414	11.7248	11.1088	11.8345	12.269
ENSG00000073756	PTGS2	0	0	0	0	0	0	0	1.0566	0	0	0	0	0	0	0	0
ENSG00000143851	PTPN7	9.7419	10.7118	9.3274	11.1583	7.6599	11.2734	10.7281	11.3304	8.382	9.2884	8.1335	9.2607	8.9737	8.7022	7.5498	9.6209
ENSG00000163661	PTX3	0	1.1043	0	0	0	0	0	0	0	0	0	0	0	0	0	0
ENSG00000110400	PVRL1	9.913	10.6562	9.3706	11.061	8.3288	11.1923	10.6625	10.8943	8.9303	9.6376	8.4195	9.654	9.8276	9.4441	7.7208	9.2788
ENSG00000151576	QTRTD1	9.4661	9.9934	9.1448	9.9194	7.8045	10.1109	10.3333	10.3109	9.5857	9.9268	8.9523	9.9706	10.1331	9.7044	8.6712	10.3976
ENSG00000117280	RAB7L1	12.1669	11.9066	11.2114	11.3431	9.4608	10.9942	12.4865	11.6344	11.587	11.8058	11.0417	11.4697	11.7639	10.8634	10.3804	11.7373
ENSG00000144134	RABL2A	6.2863	6.5416	6.2378	6.5141	4.4803	6.6207	5.4594	5.7816	4.7043	5.6418	6.0097	6.3192	6.0485	5.4931	2.7845	4.1739
ENSG00000079974	RABL2B	8.1164	7.978	8.0213	8.7661	6.8449	7.8962	7.4894	7.673	6.9588	7.9452	7.4158	8.0355	7.6404	7.2401	5.6542	6.6605
ENSG00000128340	RAC2	12.0856	11.7918	12.2824	12.4	8.7284	12.0476	12.2167	11.5707	11.7104	11.4796	11.8624	12.1852	12.3963	11.575	9.6364	11.5089
ENSG00000172575	RASGRP1	10.1081	9.7642	9.0793	8.9791	7.1592	8.7887	10.1622	9.3942	8.6268	8.7334	6.3925	7.7105	7.9363	7.2825	6.9544	8.6688
ENSG00000107551	RASSF4	8.5009	9.1374	9.6111	10.3489	7.6975	9.6661	8.5821	8.2556	9.2488	9.7882	9.255	10.1527	10.105	9.578	8.215	9.8811
ENSG00000139687	RB1	12.3345	12.3253	12.3628	12.6592	10.4182	12.544	12.6985	12.5664	11.9546	12.0439	12.0841	12.3785	12.859	12.0324	11.0012	12.6152
ENSG00000023287	RB1CC1	8.3349	8.6506	8.7612	9.3025	6.0217	8.7237	8.5383	8.3637	9.2624	8.999	9.7373	10.2506	10.3968	9.5801	7.6109	9.5261
ENSG00000162521	RBBP4	11.4991	11.4644	10.9882	11.8083	8.3377	11.6994	11.9686	11.7452	10.0906	9.9431	9.9372	10.6159	10.7716	10.1087	8.412	10.4655
ENSG00000104856	RELB	9.9156	9.4938	8.7362	9.1588	8.1026	8.766	9.9707	9.3614	9.7482	11.0644	9.0238	9.3541	9.4147	8.7969	8.9414	9.5072
ENSG00000131378	RFTN1	11.9164	11.367	10.6391	10.3319	8.5398	9.5228	11.2372	10.4343	11.9595	12.6591	12.3914	13.0575	12.826	12.1745	10.6254	11.8795
ENSG00000064490	RFXANK	8.1429	7.7188	8.0057	8.1434	6.9103	8.1148	8.4185	7.8388	8.884	9.9103	9.3591	9.6694	9.77	9.0727	8.2848	9.1494
ENSG00000143344	RGL1	8.1646	8.2033	6.7681	7.3591	5.101	7.0899	8.3818	8.3964	10.4669	10.9243	9.5668	10.5695	10.3723	9.9046	8.8384	10.7737
ENSG00000090104	RGS1	9.8164	10.1302	8.8998	10.0545	7.1859	9.2624	9.4757	9.3567	6.8784	8.0563	6.4856	7.2084	5.1526	4.8694	7.0296	6.5826
ENSG00000143333	RGS16	6.755	6.9968	7.1263	8.076	6.5385	8.1207	8.0485	7.6882	5.6761	6.6615	4.5478	4.9146	6.2677	6.1727	6.2506	7.1463
ENSG00000167550	RHEBL1	6.9772	6.5416	6.0275	7.207	5.709	6.1711	7.1829	6.5195	5.6488	5.8537	5.9885	5.8369	5.537	4.4886	4.8207	5.7336
ENSG00000172602	RND1	7.6098	6.8887	7.1735	7.4395	4.437	6.8095	6.9463	6.3954	4.4731	4.7148	4.153	4.7506	4.5065	4.627	2.6229	3.669
ENSG00000137522	RNF121	9.631	9.3227	8.9054	9.1852	7.6599	8.9105	9.5158	8.9249	8.8632	9.2866	8.3007	9.0911	9.1088	8.626	7.5555	8.9556
ENSG00000165188	RNF183	7.3317	6.201	4.9444	4.7458	1.8758	4.3561	7.5787	6.4499	2.7613	3.7687	1.926	0	0.9928	0	4.6616	4.843
ENSG00000063978	RNF4	9.7098	9.8314	9.3378	10.0369	7.9242	10.0482	10.1789	10.1934	8.8869	9.0727	8.7039	9.1931	9.5595	8.9854	7.6902	9.5072
ENSG00000166441	RPL27A	15.1782	14.3623	14.982	14.8346	15.2427	14.3215	14.6867	13.89	14.1342	15.7926	14.6203	14.3862	14.5751	13.8451	14.3056	13.8792
ENSG00000198918	RPL39	12.5152	11.1791	12.153	11.6769	12.6205	11.0967	11.841	10.7995	11.3352	12.6251	12.0931	11.2111	11.721	10.5534	11.5589	10.6346
ENSG00000163923	RPL39L	7.8589	6.846	6.5702	6.8142	7.3888	6.6375	8.2656	7.2086	7.4232	8.5572	7.6347	6.6646	7.2951	6.0551	7.8554	7.1678
ENSG00000146223	RPL7L1	10.8647	11.0097	10.1972	10.9524	9.3765	11.0838	11.673	11.5217	10.9159	11.4152	10.3353	11.1341	11.4225	10.7852	10.1631	11.5325
ENSG00000140988	RPS2	15.0964	14.3721	14.9743	14.8044	15.6268	14.2753	14.9132	13.9488	14.1303	16.0542	14.5004	14.1973	14.398	13.5423	14.5691	14.0702
ENSG00000233927	RPS28	13.5415	12.5223	13.4052	13.0851	14.2018	12.56	13.3661	12.2031	12.4288	14.4318	13.0381	12.6364	12.9814	11.9586	12.9736	12.1557
ENSG00000175634	RPS6KB2	8.4167	8.0298	8.34	8.6101	6.4642	8.0785	9.4022	8.6267	8.7352	9.1963	8.6311	9.1194	9.3615	8.601	7.8736	9.6595
ENSG00000142937	RPS8	15.7295	14.9895	15.4796	15.4089	15.2992	14.9	15.3162	14.5177	14.9412	16.2772	15.3703	15.2239	15.3835	14.5786	14.8209	14.624
ENSG00000132275	RRP8	8.6832	8.6001	8.0671	8.9313	7.467	8.684	9.0869	8.7147	8.1173	9.2079	7.86	8.5579	8.7798	8.4226	7.6747	8.7988
ENSG00000102104	RS1	0	0	0	0	1.8758	0	0	0	0	0	0	1.0566	0	1.7049	0	0
ENSG00000163221	S100A12	0	0	0	0	0	0	0	0	0	0	0	0	0	0	0	0
ENSG00000188015	S100A3	0	0	0	0	0	0	0	1.6599	0	0	0	0	0	0	0	1.0496
ENSG00000196154	S100A4	8.4594	8.3683	7.4365	8.1847	8.673	8.1148	9.1991	8.1277	6.3572	7.6824	5.1502	5.4303	6.4901	5.3406	7.169	5.9229
ENSG00000163220	S100A9	0	0	0	0	0	0	0	1.0566	0.9709	1.3674	0	0	0	0	0	0
ENSG00000171643	S100Z	0	0	3.079	2.7803	2.8074	2.4803	0	2.0841	0	3.0179	3.8135	3.9107	3.9837	3.3262	1.3045	1.0496
ENSG00000177409	SAMD9L	11.7118	12.159	12.2366	13.0841	9.7221	12.5231	10.9734	11.1364	9.5435	9.6014	10.3332	11.1355	11.7407	10.8244	7.8415	10.0384
ENSG00000155307	SAMSN1	9.531	9.7507	9.344	10.2438	7.8676	10.0653	9.7816	9.6829	9.7188	9.845	9.2484	8.9085	9.6563	8.7062	8.7249	9.7081
ENSG00000073060	SCARB1	8.8822	8.663	7.9084	8.7739	7.4778	9.2489	10.4264	9.8374	8.6127	9.5703	7.6879	8.9085	8.9623	8.7932	8.215	10.0457
ENSG00000132330	SCLY	6.3782	6.2673	5.706	6.3368	4.8575	5.8654	7.1541	6.4499	5.5922	6.9647	5.2596	6.0161	5.5972	5.3406	5.5217	6.5168
ENSG00000128228	SDF2L1	6.5329	5.9284	6.5125	6.2054	7.1389	6.384	7.649	7.198	6.5624	8.5049	7.05	6.9046	6.9364	6.4584	7.3297	7.4487
ENSG00000188404	SELL	11.8854	10.987	10.0351	9.6447	9.7503	9.6517	11.9267	10.7433	10.7179	11.4293	8.7976	9.0853	9.552	8.8543	10.5345	11.048
ENSG00000110876	SELPLG	6.2544	6.201	7.1454	7.9308	6.4087	7.5443	6.1252	6.154	4.9932	5.6418	6.4705	7.0051	7.0046	6.2813	3.4995	5.2184
ENSG00000138623	SEMA7A	8.4418	8.5027	6.8517	7.1244	5.0144	7.3451	9.0535	8.486	10.1071	10.3653	9.0341	10.2759	10.236	10.1408	8.5516	10.7026
ENSG00000178980	SEPW1	7.5778	6.846	7.1828	7.5247	6.9805	6.7339	7.5422	6.9305	6.9018	8.6565	7.6482	7.6183	7.5521	6.8741	7.0538	7.261
ENSG00000197632	SERPINB2	0	0	0	0	0	0	0	0	0	0	0	0	0	0	0	0
ENSG00000106366	SERPINE1	1.4383	2.9837	0.9486	4.218	1.585	4.0909	0.9709	3.269	0	0	0	0	0	0	0	0
ENSG00000118515	SGK1	4.1102	6.0081	3.6053	6.1348	1.2203	6.0007	6.2207	6.5696	7.9304	8.5205	5.8555	6.1755	6.3916	6.3429	8.0363	9.3536

Ensembl Gene ID	Gene Symbol	LY10 24HR A a Log2 (TMM Counts+ 1)	LY10 24HR A b Log2 (TMM Counts+ 1)	LY10 24HR C a Log2 (TMM Counts+ 1)	LY10 24HR C b Log2 (TMM Counts+ 1)	LY10 24HR I a Log2 (TMM Counts+ 1)	LY10 24HR I b Log2 (TMM Counts+ 1)	LY10 24HR V a Log2 (TMM Counts+ 1)	LY10 24HR V b Log2 (TMM Counts+ 1)	TMD8 24HR A a Log2 (TMM Counts+ 1)	TMD8 24HR A b Log2 (TMM Counts+ 1)	TMD8 24HR C a Log2 (TMM Counts+ 1)	TMD8 24HR C b Log2 (TMM Counts+ 1)	TMD8 24HR I a Log2 (TMM Counts+ 1)	TMD8 24HR I b Log2 (TMM Counts+ 1)	TMD8 24HR V a Log2 (TMM Counts+ 1)	TMD8 24HR V b Log2 (TMM Counts+ 1)
ENSG00000163082	SGPP2	8.7565	8.9248	7.2549	7.8302	3.0583	7.2396	8.3054	8.6618	7.5474	6.7803	6.0507	7.1764	6.913	6.3226	5.3757	7.8394
ENSG00000189410	SH2D5	3.4944	2.488	0	1.7442	0	2.4803	4.9364	4.6915	4.0383	4	3.7148	1.0566	1.9855	2.9579	4.2795	5.5157
ENSG00000198053	SIRPA	9.1556	9.1724	7.5977	8.4353	6.3152	8.6678	9.4083	9.595	8.5547	9.4792	7.7266	8.2706	8.5135	7.9558	7.5611	9.0103
ENSG00000101307	SIRPB1	0.8953	0	0	2.1763	0	0	1.9523	1.0566	0.9709	0	3.2311	2.0841	2.5705	2.4647	0	2.6622
ENSG00000068903	SIRT2	8.4664	8.14	8.7108	8.8747	7.6067	8.7698	8.4425	8.0057	8.8177	9.9127	9.3612	9.9392	9.7219	9.2383	8.2328	9.231
ENSG00000158714	SLAMF8	0	0	0	0	0	0	0	0	0	0	0	0	0	0	0	0
ENSG00000165449	SLC16A9	9.2519	9.0985	5.5429	6.8586	5.1285	7.0152	9.6727	9.3543	7.1275	7.6879	1.926	2.6781	3.568	4.0712	5.9404	7.2099
ENSG00000079215	SLC1A3	3.4944	3.3533	0	3.0072	2.5008	2.4803	4.3971	3.4222	3.5361	2.7049	0	1.0566	2.3074	1.7049	2.2265	3.9928
ENSG00000115840	SLC25A12	8.9412	8.4609	8.5835	8.7267	6.4642	8.4815	8.998	8.3778	8.9217	9.5583	8.767	9.5887	9.4899	8.9463	7.9443	9.46
ENSG00000162695	SLC30A7	8.4382	9.3175	9.0768	10.2284	6.8364	9.6517	8.929	8.912	8.5803	8.9743	9.1357	9.753	9.9465	9.0422	7.5779	9.0281
ENSG00000017483	SLC38A5	8.9285	8.5567	7.5339	7.9515	7.4002	8.9173	10.3492	10.0741	8.5948	9.1449	5.7342	6.9046	7.8151	7.8326	8.7148	10.1763
ENSG00000141873	SLC39A3	6.3482	6.1079	4.9873	6.5325	5.7452	6.2163	7.5569	6.7827	7.7353	8.8589	7.7948	8.0937	8.2206	7.7135	7.0933	8.5792
ENSG00000141424	SLC39A6	9.3384	9.2344	8.7612	9.3934	7.5616	9.3512	10.0638	9.8254	9.8971	10.2409	9.5308	9.7567	10.3153	9.6262	9.367	10.8186
ENSG00000139514	SLC7A1	9.8662	10.2053	7.9525	9.067	7.6165	9.664	11.1311	11.388	9.2647	9.5107	6.3436	8.0116	7.9537	7.8468	8.5431	10.2998
ENSG00000080503	SMARCA2	10.1402	9.9869	9.4302	10.1245	7.9477	9.7022	10.4163	9.6867	11.423	11.8916	11.3646	12.2035	12.0994	11.4571	10.0513	11.888
ENSG00000166311	SMPD1	6.188	6.5941	7.1454	7.738	6.2662	7.0899	7.0539	6.3388	6.1179	7.3677	6.9447	7.6104	7.3916	6.9682	5.2133	6.1127
ENSG00000125835	SNRPB	11.6296	10.7402	10.4086	10.3961	11.3215	10.802	12.616	11.628	11.1975	12.5717	10.4789	10.4386	11.0052	10.2966	11.7285	11.8945
ENSG00000100028	SNRPD3	10.8981	10.1361	10.0145	10.024	10.5291	10.2945	11.4722	10.9465	10.5247	11.8626	10.3994	10.2722	10.7453	10.0969	11.0386	11.0838
ENSG00000147164	SNX12	8.4418	8.5299	7.8041	8.7307	7.3539	8.7158	9.0844	8.7905	8.4432	9.2002	7.9767	8.7903	8.8214	8.3294	7.9006	9.1359
ENSG00000178996	SNX18	11.0648	10.8251	10.1063	10.5644	9.2369	10.2368	11.6453	10.7739	11.2011	11.7166	10.8625	10.9853	11.376	10.6789	10.6527	11.8937
ENSG00000120833	SOCS2	3.8777	2.488	0	1.7442	0	2.1506	4.3298	3.8032	4.4731	4.7148	2.9126	3.269	4.153	3.8629	4.7824	4.4094
ENSG00000184557	SOCS3	7.7541	5.1048	4.7586	3.5323	3.3688	2.4803	7.8296	5.8642	8.5767	9.9569	7.3431	8.1876	8.4899	8.6944	9.3213	10.6452
ENSG00000005513	SOX8	0	0	0	0	0	0	0	0	0	0	0	0	0	0	0	0
ENSG00000158480	SPATA2	7.5049	7.578	6.5272	8.0633	6.4965	8.0032	8.1196	8.0709	6.7931	7.6879	7.2665	7.4592	7.5521	6.8879	6.9459	7.6147
ENSG00000101222	SPEF1	1.4383	2.157	0	1.7442	0	1.1043	0.9709	1.6599	0.9709	0	0	2.0841	0	2.7334	0	0
ENSG00000066336	SPI1	6.8221	6.8887	7.5626	7.8451	6.6286	7.6551	7.4267	6.9305	6.7159	7.6488	7.8772	8.0236	8.3915	7.8255	6.3187	7.2202
ENSG00000183018	SPNS2	5.2407	5.1981	2.2388	4.5429	0	5.3208	5.4906	6.132	3.7581	0	1.926	3.269	2.7929	3.4803	0.7991	4.7887
ENSG00000161011	SQSTM1	10.5275	10.8711	10.6575	11.668	7.8552	11.4522	11.1549	11.0943	9.2894	8.933	9.8596	10.1554	10.1229	9.2437	7.2481	8.918
ENSG00000198911	SREBF2	12.0828	11.8546	12.2455	12.3142	10.5727	12.348	12.5058	11.9319	11.4431	11.836	11.7089	12.6693	12.7718	12.0347	9.9668	11.9658
ENSG00000112658	SRF	9.739	9.6468	9.1233	9.6864	8.5552	9.8934	10.3193	10.0928	8.916	9.8973	8.3135	9.0178	9.1867	8.7635	8.5172	9.7099
ENSG00000174780	SRP72	11.2189	10.8361	10.8862	11.2852	9.9857	11.23	11.7587	11.2856	10.6979	10.8367	10.802	10.9511	11.3888	10.5642	10.0382	11.1942
ENSG00000173465	SSSCA1	8.1986	6.9705	6.7557	7.3999	8.3198	7.3246	8.9487	8.2142	7.6479	8.5049	7.1856	7.0171	7.172	6.5996	7.4193	7.5749
ENSG00000180616	SSTR2	1.4383	1.1043	0	1.1177	0	1.1043	0	0	3.1227	1.3674	2.7225	4.4983	3.8904	4.2532	1.6826	4.2563
ENSG00000106610	STAG3L4	7.5318	7.4882	7.192	7.4874	6.4311	7.7503	7.8174	7.4052	6.0354	7.0097	6.5444	6.5857	7.0157	6.3628	6.1643	6.6298
ENSG00000214530	STARD10	5.6381	4.8972	4.7586	5.8417	5.0144	5.0976	6.044	4.915	4.9491	5.8138	5.7342	5.9165	6.0697	5.0764	4.8938	5.2987
ENSG00000115415	STAT1	10.4996	11.1955	10.0904	11.5538	8.4353	11.3678	10.5176	11.273	10.2635	10.4327	10.0795	10.5827	11.7834	10.9945	9.3328	11.0501
ENSG00000168610	STAT3	12.3273	12.1068	11.4085	11.703	9.5078	11.3864	12.7617	12.1316	11.7578	12.2632	11.293	12.4123	12.088	11.561	10.5537	12.6876
ENSG00000126561	STAT5A	9.2087	8.7658	8.1732	8.812	6.4423	7.9903	9.6228	7.9374	4.1977	4.5298	0	3.6871	3.4436	2.7334	2.4383	4.1739
ENSG00000166888	STAT6	12.966	12.9352	12.6771	13.0855	11.1081	12.9406	12.9855	12.7256	12.0022	12.361	11.8857	12.9144	12.7678	12.2339	10.2424	12.1614
ENSG00000125834	STK35	8.1385	8.3633	8.0057	8.8419	7.018	8.8072	8.6106	8.6885	7.7548	8.486	7.9112	8.8757	8.7798	8.0814	7.0538	8.4837
ENSG00000135604	STX11	4.1102	3.3533	0	2.511	0	1.1043	4.3298	4.1043	3.4114	4.135	4.2965	2.0841	3.4436	1.7049	3.4033	3.5435
ENSG00000116266	STXBP3	9.4661	9.401	8.8856	9.7028	6.8779	9.3049	9.6454	9.2301	9.1543	9.1669	9.8377	9.762	10.1114	9.1115	7.9357	9.4534
ENSG00000197321	SVIL	6.6605	6.6113	6.2557	6.6039	5.4912	6.364	6.8658	6.9558	5.4718	5.5954	4.5478	5.5348	5.4087	5.0277	4.5753	6.5984
ENSG00000171992	SYNPO	8.3614	8.3171	4.3611	6.9709	5.3334	6.7493	7.8714	6.7541	5.306	6.4303	5.3937	5.0144	5.7374	5.2145	3.9069	5.2987
ENSG00000158710	TAGLN2	12.6644	11.9797	11.411	11.3753	10.9494	11.3451	13.0887	12.2364	12.0531	12.9341	11.109	11.2891	11.5894	10.8607	11.4541	11.9142
ENSG00000231925	TAPBP	7.5517	7.3927	7.1263	7.5699	5.101	7.4529	7.4347	6.7101	7.7867	7.3608	7.4925	8.5371	8.651	7.9293	4.1052	7.5989
ENSG00000157014	TATDN2	8.7678	8.6382	8.4895	9.0638	8.0346	9.0697	9.3567	9.1055	7.7992	8.9515	7.7517	8.5987	8.486	8.1461	7.323	8.3721
ENSG00000109680	TBC1D19	5.6381	6.0336	5.68	6.2943	4.437	5.9748	6.0016	5.5683	4.342	5.9672	5.2596	5.6005	5.3402	4.7533	4.3327	4.7317
ENSG00000011007	TCEB3	9.4556	9.6343	8.6249	9.8495	7.5045	9.5693	10.1032	10.0985	9.4999	9.8338	8.5687	9.3987	9.7943	9.2782	8.7449	10.0915
ENSG00000100207	TCF20	9.6557	9.9852	9.3378	10.1678	7.0974	10.1766	10.1943	10.0999	8.5547	8.4342	8.4772	9.7239	9.7846	9.0266	6.4338	9.3323
ENSG00000070814	TCOF1	10.2252	10.3216	9.3336	10.1678	9.126	10.3361	11.4561	11.1481	9.9738	10.8129	8.6713	9.9798	9.8245	9.6487	9.1048	11.0392
ENSG00000137203	TFAP2A	2.9727	3.1795	1.9222	3.0072	2.8074	2.7506	2.7506	2.903	0.9709	0	0	1.0566	0	0	0	0
ENSG00000183434	TFDP3	0	0	0	0	0	0	0.9709	0	0	0	0	0	0	0	0	0
ENSG00000068323	TFE3	8.5009	8.8183	8.5409	9.3238	7.445	9.1447	8.9459	8.7072	7.8603	8.6621	8.0648	9.0505	8.6686	8.0934	6.6291	8.2762
ENSG00000119699	TGFB3	5.4809	5.4845	5.257	6.1348	0.7398	5.5497	5.2215	4.915	4.4094	5.2215	3.9059	6.3766	5.537	5.2998	2.9317	5.2184

Ensembl Gene ID	Gene Symbol	LY10 24HR A a Log2 (TMM Counts+ 1)	LY10 24HR A b Log2 (TMM Counts+ 1)	LY10 24HR C a Log2 (TMM Counts+ 1)	LY10 24HR C b Log2 (TMM Counts+ 1)	LY10 24HR I a Log2 (TMM Counts+ 1)	LY10 24HR I b Log2 (TMM Counts+ 1)	LY10 24HR V a Log2 (TMM Counts+ 1)	LY10 24HR V b Log2 (TMM Counts+ 1)	TMD8 24HR A a Log2 (TMM Counts+ 1)	TMD8 24HR A b Log2 (TMM Counts+ 1)	TMD8 24HR C a Log2 (TMM Counts+ 1)	TMD8 24HR C b Log2 (TMM Counts+ 1)	TMD8 24HR I a Log2 (TMM Counts+ 1)	TMD8 24HR I b Log2 (TMM Counts+ 1)	TMD8 24HR V a Log2 (TMM Counts+ 1)	TMD8 24HR V b Log2 (TMM Counts+ 1)
ENSG00000092295	TGM1	3.6993	1.7225	3.2296	3.2048	0	3.6392	2.7506	3.4222	3.5361	4.5298	3.2311	5.1953	5.2679	4.0712	1.9819	4.0858
ENSG00000102265	TIMP1	0	0	0	1.1177	2.1144	1.1043	0	1.0566	0	0.84	0	0	0	0	0	0
ENSG00000119139	TJP2	7.6957	9.2344	9.7871	11.0697	7.6456	10.1766	7.4584	8.3684	5.7811	6.0032	7.9112	8.214	8.0374	6.9939	4.0418	5.8714
ENSG00000137076	TLN1	11.7377	12.1091	11.7209	12.5055	10.4034	12.5841	12.4413	12.2601	11.5852	11.9426	11.5115	12.8914	13.044	12.5491	9.9857	12.6598
ENSG00000137462	TLR2	7.4495	7.7653	6.6921	8.057	5.1558	7.4243	7.1541	7.2086	7.3399	7.9951	6.7334	7.5862	7.6896	7.1739	5.4259	6.911
ENSG00000145107	TM4SF19	0	0	0	1.7442	0	0	0	1.0566	0.9709	0	2.5008	2.0841	1.9855	0	0.7991	0
ENSG00000110108	TMEM109	10.0024	9.8019	9.2741	9.6553	9.0519	9.6476	10.5015	10.196	9.9317	10.7822	9.2877	9.6636	10.0455	9.43	9.7144	10.6249
ENSG00000189077	TMEM120A	8.3652	7.8398	7.7174	7.992	6.5692	7.4529	7.7292	7.1766	6.9134	8.0901	7.4079	7.4503	7.3659	6.5483	6.3187	6.4656
ENSG00000244187	TMEM141	8.7507	7.2026	7.373	7.1954	8.0383	7.3349	9.3419	7.859	7.6956	8.7334	7.6074	7.4592	7.7101	7.0442	7.7358	7.7422
ENSG00000126106	TMEM53	5.4809	5.1522	5.5429	5.435	5.4256	5.921	5.9129	5.318	5.2338	5.3937	5.7594	6.1538	6.7896	5.9269	5.4009	5.5157
ENSG00000174529	TMEM81	4.6421	4.4423	5.6536	5.6859	5.26	5.6845	5.3625	5.2784	5.1185	6.7382	6.327	5.7236	5.5674	5.3406	5.4259	5.7623
ENSG00000187045	TMPRSS6	6.1363	5.6259	5.1473	4.9236	2.9373	4.5801	7.3696	5.8642	7.9304	9.2627	7.4925	8.1768	7.5895	7.4221	7.005	8.1466
ENSG00000205542	TMSB4X	13.544	12.4895	13.1869	12.9516	12.9104	12.9813	13.6233	12.7939	14.6652	15.5797	14.9462	14.5567	15.2232	14.3174	14.9291	14.8123
ENSG00000232810	TNF	4.1795	3.8895	1.516	3.0072	0	3.1731	4.0259	3.9107	4.4094	4.2593	3.6076	4.1043	3.6837	4.3363	1.3045	5.3374
ENSG00000185215	TNFAIP2	0.8953	0	2.4983	0	0.7398	1.1043	3.5248	1.0566	8.2374	8.7678	8.2836	9.0208	9.2882	8.8151	7.7992	9.2911
ENSG00000118503	TNFAIP3	10.6715	10.6878	8.8366	9.9709	7.0473	9.8222	10.8667	10.7204	10.1084	10.1376	8.4234	9.4685	9.6133	9.2193	8.8079	10.2375
ENSG00000123610	TNFAIP6	5.1391	5.2426	3.4906	5.3547	0	3.991	3.846	4.4983	2.281	2.3074	0	0	0	0	0.7991	0
ENSG00000067182	TNFRSF1A	0	2.157	0	2.511	0	2.1506	0	1.6599	0	0	0	0	0.9928	1.0909	0	0
ENSG00000146072	TNFRSF21	8.8717	9.3812	10.4282	10.8542	8.7642	10.1594	8.7833	8.5288	8.0869	8.5328	10.0029	10.4904	10.5913	9.7353	6.8477	8.1574
ENSG00000161955	TNFSF13	6.1538	5.8148	5.3909	6.3983	2.6622	6.2815	5.8425	5.5011	3.5361	4.757	4.153	5.568	5.4087	4.8694	2.6229	4.9467
ENSG00000102524	TNFSF13B	5.2079	5.7546	5.2215	6.8291	4.3448	6.0514	4.1859	5.6008	1.9598	1.7527	2.5008	3.269	3.6837	2.4647	2.2265	2.3951
ENSG00000125735	TNFSF14	4.6421	4.4423	4.4861	4.7458	1.2203	4.5801	5.2581	5.1526	2.9542	1.7527	2.5008	5.278	4.3052	4.9241	1.6826	4.0858
ENSG00000050730	TNIP3	6.9375	5.1522	4.2242	2.7803	1.8758	2.7506	7.5859	4.3533	4.903	5.5478	2.2418	2.6781	3.6837	2.1342	4.5298	6.8323
ENSG00000186283	TOR3A	9.521	9.6571	9.6782	10.0514	8.5372	10.4992	10.4522	10.4765	8.3903	9.2384	8.5863	9.1416	9.4395	8.6905	7.9572	9.1548
ENSG00000056558	TRAF1	10.6065	10.0447	8.9138	9.4766	6.9418	9.1122	10.5784	9.5767	7.7023	7.9951	6.642	8.1714	7.5821	7.1626	6.2506	8.3249
ENSG00000009790	TRAF3IP3	8.8316	9.0833	9.5134	10.0125	8.2924	9.9261	9.5802	8.8792	5.7296	6.0725	4.99	5.9919	6.3916	5.5289	5.4506	5.9229
ENSG00000054116	TRAPPC3	9.274	9.1315	9.1543	9.6926	8.6776	9.5517	9.7925	9.5582	7.8422	8.933	8.024	8.3587	8.5671	7.9227	7.8507	8.2309
ENSG00000204713	TRIM27	7.8482	7.8897	7.7174	7.9098	4.8237	8.1384	8.4385	8.2402	7.7023	6.6838	7.1949	7.918	8.2206	7.441	5.2968	7.8726
ENSG00000204616	TRIM31	0	0	0	1.1177	0	0	0	0	0	0	0	0	0	0	0	0
ENSG00000142185	TRPM2	8.9336	8.8618	9.0262	9.3264	7.4059	9.2543	9.3096	8.6695	7.5913	8.069	8.351	9.3082	9.472	9.0907	6.0244	8.1357
ENSG00000196428	TSC22D2	7.1348	7.3217	6.5842	7.738	5.2087	7.5174	7.4427	7.198	7.0137	7.4676	7.4547	7.8041	7.8024	6.9016	6.0079	7.5005
ENSG00000135452	TSPAN31	8.587	8.5699	8.5659	9.4719	6.2662	9.165	8.5567	8.0475	7.6339	7.7789	8.0343	8.7798	8.8214	8.051	6.0244	7.4748
ENSG00000158526	TSR2	8.6029	8.6382	8.145	9.0889	7.9709	9.0697	9.1095	9.1307	7.9808	9.2774	7.9979	8.5621	8.3745	8.0754	7.7308	8.6306
ENSG00000052841	TTC17	9.922	10.4836	10.0805	11.0947	8.3407	10.6332	9.8873	9.9457	9.839	10.4629	10.3467	11.6204	11.1146	10.5706	8.3383	10.066
ENSG00000168234	TTC39C	7.7992	7.3423	7.0371	7.0497	6.1491	6.8241	8.2062	7.4594	8.4392	9.0896	8.2836	9.0476	8.895	8.2568	8.0564	8.7638
ENSG00000186591	UBE2H	10.2065	10.5438	10.8059	11.6183	9.1243	11.2681	10.4113	10.2132	9.7368	10.0339	10.5353	11.3114	11.3397	10.5282	8.4088	9.5894
ENSG00000156587	UBE2L6	10.616	10.7256	9.6799	10.7301	9.6934	10.7309	10.9054	10.8983	10.1321	11.1734	10.1212	10.4341	10.8972	10.3082	10.0791	10.5744
ENSG00000109103	UNC119	8.8424	8.7427	8.2851	8.7931	7.3598	8.3304	8.8325	8.0238	10.0387	10.9178	9.1018	9.9119	9.9565	9.6548	9.1067	9.8006
ENSG00000167118	URM1	9.4218	9.0924	8.9248	9.2971	8.6706	9.2944	9.9018	9.5311	9.4864	10.672	9.4446	9.654	9.9961	9.3423	9.4161	10.0282
ENSG00000184979	USP18	4.9919	6.3512	4.6554	6.6552	2.1144	6.4421	5.5798	6.7827	5.3409	5.8138	5.4253	6.5694	7.6826	6.8741	3.757	7.0682
ENSG00000036672	USP2	1.4383	2.157	0.9486	1.1177	0	0	0.9709	1.6599	3.275	2.8699	1.926	4.0108	3.7908	4.2532	2.7845	4.3349
ENSG00000155313	USP25	9.1707	9.1492	8.8742	9.649	7.5968	9.1935	9.5856	9.2378	8.6233	8.7118	8.5182	9.0148	9.3353	8.6865	8.0524	9.2361
ENSG00000161133	USP41	0	0	0	0	0	0	0	0	0	0	0	0	1.5753	1.0909	0	1.0496
ENSG00000125753	VASP	11.3805	10.9689	10.8312	11.0939	9.9137	10.9245	11.7048	11.1987	11.2349	12.0271	10.9122	11.2032	11.5486	10.954	10.7929	11.4871
ENSG00000141968	VAV1	9.4361	9.0013	10.0879	9.9642	8.2708	10.0791	9.5748	9.2039	9.8192	10.2763	10.4064	11.0855	11.3046	10.7676	8.4761	10.8237
ENSG00000141252	VPS53	8.2482	8.4935	7.7427	9.0024	7.069	9.0418	8.8888	8.6385	8.1073	8.628	7.8943	9.0593	9.2348	8.5926	6.9883	9.0281
ENSG00000155659	VSIG4	0	0	0	0	0	0	0	0	0	0	0	0	0	0	0	0
ENSG00000120688	WBP4	8.4203	8.5829	7.4823	8.5883	6.1218	8.5885	8.929	8.7657	7.9642	8.5174	7.6347	8.0473	8.552	7.7668	7.486	8.526
ENSG00000227057	WDR46	7.6777	7.6376	6.127	7.4098	5.3092	7.6468	8.5121	8.1498	8.6127	9.1267	7.9927	8.4595	8.7863	8.6094	7.7158	9.3559
ENSG00000137207	YIPF3	9.3853	9.4302	9.5226	10.0256	8.8728	9.6762	9.4679	9.2275	9.0348	10.2578	9.7005	10.3033	10.0722	9.4394	8.5114	9.2
ENSG00000136758	YME1L1	10.8949	11.1139	10.4629	11.3161	9.4305	11.3501	11.7769	11.4743	10.6485	10.797	10.9115	11.2131	11.732	10.8616	9.9071	11.315
ENSG00000166913	YWHAB	11.5703	11.7266	11.4831	11.9174	10.9547	12.0842	12.2228	12.1106	11.6925	12.5166	11.8893	12.1728	12.5137	11.736	11.3216	12.2109
ENSG00000214717	ZBED1	0	0	0	0	0	0	0	0	0	0	0	0	0	0	0	0
ENSG00000066422	ZBTB11	9.6356	9.8833	9.4341	10.3306	7.2441	9.8689	10.1379	9.6772	9.2211	9.0958	9.4543	9.9264	10.0696	9.43	7.7606	9.5282
ENSG00000168228	ZCCHC4	7.324	8.0234	6.9956	7.738	5.7452	7.8962	8.2656	8.2142	6.4869	6.2172	6.6819	7.0876	7.1621	6.697	5.4744	7.1678

Ensembl Gene ID	Gene Symbol	LY10 24HR A a Log2 (TMM Counts+ 1)	LY10 24HR A b Log2 (TMM Counts+ 1)	LY10 24HR C a Log2 (TMM Counts+ 1)	LY10 24HR C b Log2 (TMM Counts+ 1)	LY10 24HR I a Log2 (TMM Counts+ 1)	LY10 24HR I b Log2 (TMM Counts+ 1)	LY10 24HR V a Log2 (TMM Counts+ 1)	LY10 24HR V b Log2 (TMM Counts+ 1)	TMD8 24HR A a Log2 (TMM Counts+ 1)	TMD8 24HR A b Log2 (TMM Counts+ 1)	TMD8 24HR C a Log2 (TMM Counts+ 1)	TMD8 24HR C b Log2 (TMM Counts+ 1)	TMD8 24HR I a Log2 (TMM Counts+ 1)	TMD8 24HR I b Log2 (TMM Counts+ 1)	TMD8 24HR V a Log2 (TMM Counts+ 1)	TMD8 24HR V b Log2 (TMM Counts+ 1)
ENSG00000023041	ZDHHC6	9.7346	9.7888	9.2479	10.1062	8.1026	9.6863	10.0444	9.7942	9.0894	9.8768	8.9332	9.7852	9.7879	9.3499	8.6764	9.8228
ENSG00000136367	ZFHX2	4.0365	5.4469	4.2242	5.5113	3.0583	5.5847	5.0657	4.6915	0.9709	1.3674	3.8135	2.6781	3.1538	2.4647	1.3045	3.8924
ENSG00000185650	ZFP36L1	11.059	11.323	10.478	10.6876	8.8206	10.0134	10.4153	10.2849	9.9724	10.8881	9.4291	10.2261	9.7337	9.5844	8.4244	9.5814
ENSG00000122515	ZMIZ2	11.1504	10.592	11.0891	10.9618	9.0895	10.6601	11.3258	10.5573	10.6258	11.0305	10.2919	11.7603	10.9665	10.9614	8.6659	10.7885
ENSG00000171940	ZNF217	9.6754	10.0525	9.4106	10.2879	7.225	10.3142	10.3085	10.188	10.1773	9.6561	9.9495	10.8668	11.3739	10.3185	8.679	10.5864
ENSG00000159905	ZNF221	0.8953	2.157	2.2388	3.6713	0	2.1506	1.541	0	0	0	0	0	0	0	0	0
ENSG00000167637	ZNF283	5.7339	6.1079	5.7315	7.0239	3.6993	6.2163	5.717	5.394	5.9923	6.5322	6.3102	6.7394	7.172	6.9419	4.6616	6.6906
ENSG00000198026	ZNF335	8.9562	9.1016	8.8512	9.5553	7.199	9.3886	9.4022	9.1853	8.3093	9.1569	8.4582	9.6694	9.3745	9.1145	6.8842	9.1869
ENSG00000186918	ZNF395	8.4699	8.8293	8.7108	9.4184	7.6116	9.4748	9.3981	9.1986	7.709	8.5297	8.1335	8.5159	9.0265	8.3193	7.8229	8.4577
ENSG00000168813	ZNF507	8.9954	9.503	8.8188	9.7838	7.3182	9.6661	9.6454	9.6347	9.2141	9.3226	8.9109	10.0403	10.3604	9.6262	7.8736	10.1536
ENSG00000183647	ZNF530	0	0	0	0	0	0	0	0	8.6268	9.0854	8.0084	9.1416	8.7337	8.3294	7.7556	8.9116
ENSG00000171425	ZNF581	9.631	9.3356	9.3543	10.1781	9.5052	9.2435	9.5382	9.1223	10.3954	11.7818	10.9233	10.9273	10.8478	10.2263	10.2256	10.3827
ENSG00000065029	ZNF76	7.0254	7.0851	7.2983	7.6731	5.8657	7.3143	7.2568	6.9683	6.7931	6.7275	7.4392	8.3202	7.8339	7.5836	5.4259	7.1678
ENSG00000142409	ZNF787	9.0708	8.2855	9.2053	9.1996	9.2177	8.6098	9.5999	8.6847	9.3777	11.0299	9.5453	9.1904	9.5155	8.5056	9.6053	9.5814
ENSG00000106400	ZNHIT1	9.4397	8.781	9.3767	9.6864	10.0166	9.5025	9.8126	9.1195	8.3986	9.5658	9.1357	9.6482	9.3264	8.7673	8.2814	8.6987
